# Transcriptome analyses of mouse and human mammary cell subpopulations reveal multiple conserved genes and pathways

**DOI:** 10.1186/bcr2560

**Published:** 2010-03-26

**Authors:** Elgene Lim, Di Wu, Bhupinder Pal, Toula Bouras, Marie-Liesse Asselin-Labat, François Vaillant, Hideo Yagita, Geoffrey J Lindeman, Gordon K Smyth, Jane E Visvader

**Affiliations:** 1 The Walter and Eliza Hall Institute of Medical Research, 1G Royal Parade, Parkville, VIC 3052, Australia; 2 Department of Medical Biology, The University of Melbourne, Parkville, VIC 3010, Australia; 3 Department of Immunology, Juntendo University School of Medicine, 2-1-1 Hongo, Bunkyo-ku, Tokyo 113-8421, Japan; 4 Department of Medical Oncology, The Royal Melbourne Hospital, Grattan St, Parkville, VIC 3050, Australia; 5 Department of Medicine, The University of Melbourne, The Royal Melbourne Hospital, Clinical Sciences Building, Parkville, VIC 3050, Australia

## Abstract

**Introduction:**

Molecular characterization of the normal epithelial cell types that reside in the mammary gland is an important step toward understanding pathways that regulate self-renewal, lineage commitment, and differentiation along the hierarchy. Here we determined the gene expression signatures of four distinct subpopulations isolated from the mouse mammary gland. The epithelial cell signatures were used to interrogate mouse models of mammary tumorigenesis and to compare with their normal human counterpart subsets to identify conserved genes and networks.

**Methods:**

RNA was prepared from freshly sorted mouse mammary cell subpopulations (mammary stem cell (MaSC)-enriched, committed luminal progenitor, mature luminal and stromal cell) and used for gene expression profiling analysis on the Illumina platform. Gene signatures were derived and compared with those previously reported for the analogous normal human mammary cell subpopulations. The mouse and human epithelial subset signatures were then subjected to Ingenuity Pathway Analysis (IPA) to identify conserved pathways.

**Results:**

The four mouse mammary cell subpopulations exhibited distinct gene signatures. Comparison of these signatures with the molecular profiles of different mouse models of mammary tumorigenesis revealed that tumors arising in *MMTV*-*Wnt-1 *and *p53*^-/- ^mice were enriched for MaSC-subset genes, whereas the gene profiles of *MMTV*-*Neu *and *MMTV*-*PyMT *tumors were most concordant with the luminal progenitor cell signature. Comparison of the mouse mammary epithelial cell signatures with their human counterparts revealed substantial conservation of genes, whereas IPA highlighted a number of conserved pathways in the three epithelial subsets.

**Conclusions:**

The conservation of genes and pathways across species further validates the use of the mouse as a model to study mammary gland development and highlights pathways that are likely to govern cell-fate decisions and differentiation. It is noteworthy that many of the conserved genes in the MaSC population have been considered as epithelial-mesenchymal transition (EMT) signature genes. Therefore, the expression of these genes in tumor cells may reflect basal epithelial cell characteristics and not necessarily cells that have undergone an EMT. Comparative analyses of normal mouse epithelial subsets with murine tumor models have implicated distinct cell types in contributing to tumorigenesis in the different models.

## Introduction

The mammary gland comprises a ductal epithelial network embedded in a stromal matrix. The ducts are composed of an inner layer of luminal cells and an outer layer of myoepithelial cells. Pregnancy is accompanied by the expansion and differentiation of alveolar luminal cells, resulting in secretory cells that produce and secrete milk. Although the function of the mammary gland is preserved across species, marked anatomic differences exist between human and mouse mammary tissue. The human mammary gland is characterised by a branching network of ducts that terminate in clusters of small ductules that constitute the terminal ductal lobular units (TDLUs). In contrast, the mouse mammary epithelial tree does not contain TDLUs, although small alveolar buds are formed during each estrous cycle. Moreover, the human breast parenchyma is significantly more fibrous than the mouse stroma, which contains predominantly adipocytes. Despite these architectural differences, accumulating evidence suggests that remarkable parallels are found between the hierarchy of epithelial cells that exist in the mammary glands of humans and mice [1].

Distinct epithelial subtypes have been prospectively isolated from both mouse [2-5] and human mammary glands [6-10]. Functionally analogous subpopulations have been identified: the MaSC-enriched/bipotent progenitor, committed luminal progenitor and mature luminal cell subsets. In the mouse, MaSCs are found within the basal CD49f^hi^CD29^hi^CD24^+^Sca1^- ^subset (referred to as MaSC-enriched), whereas committed luminal progenitor cells exhibit a CD29^lo^CD24^+^CD61^+ ^(or Sca-1^-^CD24^+^) phenotype, and mature luminal cells display a CD29^lo^CD24^+^CD61^-^phenotype [2,3]. In human mammary tissue, the CD49f^hi^EpCAM^-/lo ^subpopulation has been demonstrated to be enriched for MaSCs, based on *in vivo *transplantation either into the mouse mammary fat pad [7] or under the renal capsule [6]. Luminal progenitor and differentiated cells prospectively isolated from human breast tissue are characterized by CD49f^hi^EpCAM^+ ^and CD49f^-^EpCAM^+ ^phenotypes, respectively.

There are similarities as well as species-specific differences in the expression of cell-surface markers on the epithelial subsets. Both the mouse and human MaSC-enriched populations express high levels of CD49f. However, CD24 is a marker of epithelial cells in the mouse mammary gland, but not in human breast tissue, where it specifically marks luminal cells [3-5,7,11]. Significantly, both the human and mammary MaSC-enriched populations lack expression of the steroid hormone receptors ERα and PR [7,12]. Moreover, these MaSCs do not express detectable levels of ERBB2/HER2, reminiscent of the triple-negative receptor phenotype that characterizes many basal cancers [13].

Understanding the relation between normal epithelial cell types and the different molecular subtypes of breast cancer is fundamental to gaining insight into cell types predisposed to carcinogenesis. At least six distinct subtypes of breast tumors have been defined on the basis of gene expression profiling. These include the luminal A and B, basal-like, claudin-low, HER2/ERBB2-overexpressing, and normal breast-like subtypes [14]. We recently used the emerging human mammary hierarchy as a framework for understanding aberrant cell subsets that may arise during breast oncogenesis [7]. The claudin-low subtype was found to be most closely associated with the gene signature of the MaSC-enriched population, whereas the molecular profiles of the basal-like subtype of breast cancer showed remarkable concordance with the luminal progenitor gene signature. Not surprisingly, the expression profiles of the luminal A and B subtypes were closest to that of mature luminal epithelial cells. Interestingly, the molecular portrait of premalignant tissue from *BRCA1 *mutation carriers, who usually develop basal-like breast cancers, showed striking similarity to the luminal progenitor signature [7].

In the context of the mouse mammary gland, transcriptome analyses of epithelial cells have highlighted the differences between basal and luminal cells and revealed a number of potential regulators [5,15]. Here we performed genome-wide transcriptome analyses of three different mouse epithelial subpopulations and established pathways that are conserved in functionally equivalent subsets in humans by using specific gene signatures. We further used these signatures to interrogate mouse models of mammary tumors, providing insight into cell types that contribute to breast oncogenesis.

## Materials and methods

### Mice and mammary cell preparations

A single cell suspension of mammary cells was prepared from freshly harvested mammary glands and sorted by flow cytometry, as previously described [3]. Mice were on a pure FVB/N background. All experiments were approved by the WEHI Animal Ethics Committee, and the care of animals was in accordance with institutional guidelines. Experiments using human tissue obtained from the Royal Melbourne Hospital Tissue Bank were approved by the Human Research Ethics Committees of The Walter and Eliza Hall Institute of Medical Research and Melbourne Health.

### Antibodies, staining and cell sorting

Unless otherwise specified, antibodies for flow cytometry were obtained from BD Pharmingen. Antibodies against mouse antigens were PE-conjugated antibody to CD24, FITC-conjugated antibody to CD29 (clone HMbeta1-1 from H. Yagita) [16], biotin-conjugated antibodies to CD31, CD45, and TER119, and APC-conjugated antibody to CD61 (Caltag). Antibodies used for human antigens have previously been described [7]. The Alexa Fluor 647 anti-human CD24 antibody (Biolegend) was used for analysis of human breast epithelial subsets. Antibody staining and cell sorting was as previously described [3]. Data were analyzed by using WEASEL software [17].

### RNA preparation and quantitative RT-PCR analysis

Total RNA was isolated from primary mammary cell subpopulations with the RNeasy Micro kit (Qiagen). Reverse transcription by using oligo(dT) primer and Moloney murine leukemia virus reverse transcriptase (Invitrogen) was according to the manufacturer's protocol. Quantitative RT-PCR was carried out by using a Rotorgene RG-6000 (Corbett Research) and SensiMix (dT) DNA kit (Quantace) under the following conditions: 10 min at 95°C followed by 35 cycles consisting of 15 seconds at 95°C, 20 seconds at 62°C, and 20 seconds at 72°C. Gene expression was determined with the Rotor-Gene software (version 1.7). The primer sequences used are listed in Supplementary Methods in Additional file 1.

### Microarray hybridizations

Total RNA was purified from sorted cell populations by using the RNeasy Micro kit (Qiagen). RNA quality was assessed with the Agilent Bioanalyzer 2100 (Agilent Technologies) by using the Agilent RNA 6000 Nanokit (Agilent Technologies) according to the manufacturer's protocol. Up to 500 ng of RNA was labeled with the standard Total Prep RNA amplification kit (Ambion), and complementary RNA (1.5 μg) was hybridized to Illumina MouseWG-6 v2.0 BeadChips. After washing, the chips were coupled with Cy3 and scanned by using an Illumina BeadArray Reader. Unnormalized summary probe profiles, with associated probe annotation, were output from BeadStudio.

### Statistical analyses

Microarray data were analyzed by using the limma package of the Bioconductor open-source software project [18,19], as described in more detail later.

#### Microarray data analysis: normal cell subpopulations

Raw intensities were normalized by using the neqc function, which performs normexp background correction and quantile normalization by using control probes [20]. Probes were filtered if not detected in any sample (detection *p *value, 0.01). The mouse data are deposited as GEO series GSE19446, and the human, as GSE16997.

#### Microarray data analysis: mouse model tumors

Expression profiles of mouse tumors were downloaded from GEO series GSE3165 [21]. Fifty-six Agilent arrays (Agilent-011978 Mouse Microarray G4121A) profiling mouse tumor models of interest were included in the analysis. The samples and arrays are described by Herschkowitz *et al*. [14]. Data analysis used the raw Agilent Feature Extraction data files and probe annotation from GEO. Control probes were filtered, and then expression values were normexp background corrected with offset 16 [20], and then log-ratios were global loess normalized [22]. Two *MMTV*-*Wnt-1 *samples and one *MMTV*-*Neu *sample were removed as outliers on the basis of unsupervised clustering.

#### Subpopulation expression signatures

Pairwise comparisons were made between the three epithelial cell populations by using empiric Bayes-moderated *t *statistics [19] and array quality weights [23]. Allowance was made for possible correlations between RNA samples drawn from the same pool of mice [24]. The false discovery rate (FDR) was controlled by using the Benjamini and Hochberg algorithm. Probes with FDR < 0.05 and fold-change > 1.5 were judged to be differentially expressed. For each subpopulation (MaSC-enriched, luminal progenitor, and mature luminal), signature probes were defined as those that were significantly differentially expressed in the same direction versus both of the other two cell subpopulations. For stromal cells, the signature probes were defined relative to the three epithelial cell populations.

For each target sample (mouse tumor or normal mouse mammary cell subpopulation), a set of signature scores was computed to measure the transcriptional activity of each mouse cell subpopulation in that sample, by using a method previously described [7]. The signature score is essentially the average log-expression of the signature genes in the target sample, weighted by the direction and magnitude of change of those genes in the mouse subpopulation used to define the signature. Higher scores indicate that the transcriptional signature of the mouse cell subpopulation is found in the target sample.

#### Conserved signature genes

A larger set of mouse signature genes were defined by using the "nestedF" multiple-testing option of limma with FDR < 0.1. The 1.5 fold-change threshold was maintained. Mouse and human probes were matched by gene symbol by using the Jackson Laboratory orthology report of 13 November 2009 [25]. If multiple probes mapped to the same symbol, the probe with the highest average log-expression was used. Human signature genes were defined as for mouse, with the multiple testing step repeated only for those human genes with orthologues among the mouse signature genes.

#### Ingenuity pathway analysis

Ingenuity Pathway Analysis (IPA) [26] was conducted on conserved signature genes. For the MaSC-enriched subpopulation, only the top 300 signature genes were used, to make the numbers comparable for the different subpopulations. The signature sets were overlaid with canonic pathways. Canonic pathways were selected based on known biologic significance of the most highly overlapping pathways, and were displayed by using "subcellular layout". Direct associations between signature genes were drawn by using the "connect" option. The luminal progenitor signature set was too small to generate connections, so direct associations were drawn from the *KIT *and *CYP24A1 *genes by using the "grow" option. Genes without connections to other signature genes were removed from the final figures.

## Results

### Derivation of distinct gene signatures for mouse mammary cell subpopulations

**Figure 1:**
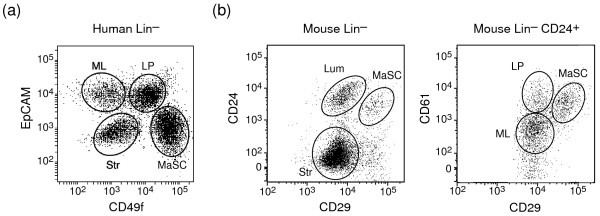
**Comparison of analogous human and mouse mammary cell subsets**. Representative FACS dotplots of **(a) **Lin^- ^human mammary cells isolated from the reduction mastectomy specimen of a 27-year-old woman labeled with CD49f and EpCAM antibodies and **(b) **Lin^- ^mouse mammary cells isolated from 8-week-old virgin FVB/N mice labeled with CD24, CD29, and CD61 antibodies. The four analogous mammary subsets correspond to the MaSC-enriched (human CD49f^hi^EpCAM^-^; mouse CD29^hi^CD24^+^CD61^+^), luminal progenitor (human CD49f^+^EpCAM^+^; mouse CD29^lo^CD24^+^CD61^+^), mature luminal (human CD49f^-^EpCAM^+^; mouse CD29^lo^CD24^+^CD61^-^), and stromal (human CD49f^-^EpCAM^-^; mouse CD29^lo^CD24^-^) mammary cell subpopulations. LP: luminal progenitor; Lum: luminal; ML: mature luminal; MS: MaSC-enriched;   Str: stromal.

Freshly sorted cell subpopulations (> 90% purity) were prepared from mouse mammary glands for gene profiling analysis. These subpopulations corresponded to the MaSC-enriched (CD29^hi^CD24^lo^CD61^+^), luminal progenitor (CD29^lo^CD24^+^CD61^+^), mature luminal (CD29^lo^CD24^+^CD61^-^), and stromal cell (CD29^lo^CD24^-^) fractions. Representative FACS dot plots depicting the four mouse cellular subsets and comparison with the analogous subpopulations found in human breast tissue [7] are shown in Figure 1. For human mammary cells, the subpopulations include the MaSC-enriched (CD49f^hi^EpCAM^-^), luminal progenitor (CD49f^+^EpCAM^+^), mature luminal (CD49f^-^EpCAM^+^), and stromal cell (CD49f^-^EpCAM^-^) fractions. Although CD24 is expressed in all epithelial subsets in mouse mammary epithelium [3-5], within human breast tissue, it marks luminal progenitor and mature luminal cells (Figure S1 in Additional file 2).

The Illumina mouse WG-6 v2.0 platform was used for arraying the four murine cell subpopulations, incorporating five biologic replicates for the three epithelial populations and three replicates for the stromal subset. Importantly, the RNA was not subjected to an amplification step before preparation of labeled cRNA, to avoid potential skewing of expression data. Unsupervised clustering revealed that the four subpopulations had distinct gene expression profiles (Figure S2 in Additional file 3). Gene expression signatures were derived for the four murine cell subpopulations, by using a method we applied previously to the analogous human subpopulations [7]. In brief, signature genes were chosen that were consistently up- or downregulated in that subpopulation (with fold-change at least 1.5 and FDR < 0.05) versus each of the other populations (Table 1). This selects a set of signature genes that strongly characterize each subpopulation by their unusually high or low transcriptional activity.

**Table 1:** Number of signature probes and genes for each mouse cell subpopulation (stringent criteria)

Mammary epithelial cell subsets	Mouse probes	Unique mouse genes	Mouse genes with human orthologues
**MaSC-enriched**			
Upregulated	2,616	1,790	1,438
Downregulated	2,305	1,620	1,316
**Luminal progenitor**			
Upregulated	521	373	286
Downregulated	170	132	111
**Mature luminal**			
Upregulated	1,026	733	569
Downregulated	1,034	740	624
**Stroma**			
Upregulated	1,933	1,273	998
Downregulated	1,264	911	773

Stringent signature probes were chosen by using fold-change > 1.5 and global FDR < 0.05.

### Mouse gene signatures correlate with specific mouse models of breast cancer

**Figure 2:**
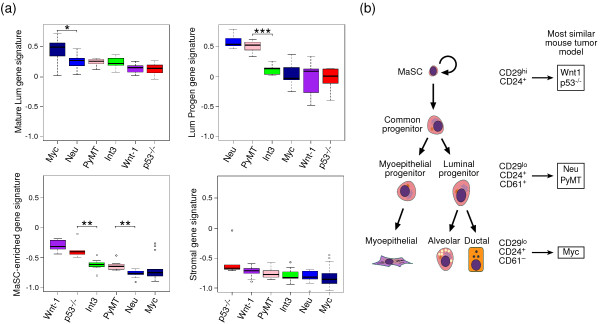
**Comparison of gene expression profiles of normal mouse mammary cell subsets with mammary tumor models**. **(a) **Box plots of signature expression scores by the mouse mammary tumor model for each subset. The MaSC-enriched signature scores are highest in the *MMTV*-*Wnt-1 *and *p53*^-/- ^tumors, whereas the luminal progenitor signature scores are highest in the *MMTV*-*Neu *and *MMTV*-*PyMT *models. *P < 0.05, **P < 0.01, ***P < 0.001. Mammary tumor datasets are from Herschkowitz *et al*. [14]. **(b) **Schematic model of the mouse mammary epithelial hierarchy and possible relations with mouse mammary tumor models. Subpopulations containing MaSCs, luminal progenitor, and mature luminal cells are defined by differential expression of CD24, CD29, and CD61. Gene expression profiling of these subpopulations revealed similarities to specific mouse mammary tumor models, depicted on the right side.

First, we used the signature genes to identify relations between tumor cells and normal epithelial cell types. Genetically engineered mouse models of mammary tumorigenesis have been previously described and include the mouse mammary tumor virus *(MMTV)-Wnt-1*, *MMTV*-*Neu*, *MMTV*-*PyMT*, *WAP*-*Myc*, *WAP*-*Int3 *(*Notch-1*) transgenic, and *p53*-null mouse models [27]. We interrogated the expression profiles of whole mammary tumors isolated from these mouse models [14] for the expression signatures characteristic of our mouse MaSC-enriched, luminal progenitor, mature luminal, and stromal subpopulations. These analyses were carried in an analogous manner to that described for comparison of human mammary cells with the different breast cancer subtypes [7]. In brief, the signature genes for each subpopulation were used to construct an index of transcriptional activity characteristic of that subpopulation. These indices, or expression signatures, were then computed for each tumor sample. The MaSC transcriptional signature was found to be highest in *MMTV*-*Wnt-1 *and *p53*^-/- ^tumors (Figure 2a). Robust results were obtained even though the *p53*^-/- ^tumors are on a different background compared with the other tumor types (BALB/c versus FVB/N). In contrast, the luminal progenitor signature was highest in *MMTV*-*Neu *and *MMTV*-*PyMT *tumors. The mature luminal signature was highest in *MMTV*-*Myc *tumors. The tumors arising in *MMTV*-*Int3 *mice did not correspond to a specific subset within the mammary epithelial hierarchy. As anticipated, the mouse stromal signature was not apparent in any of the mammary tumor profiles, thus reflecting the epithelial content of the tumors. Figure 2b summarizes potential relations between normal epithelial cell types and commonly used models of mammary tumorigenesis.

### Comparison of the expression profiles of human and mouse subpopulations

**Figure 3:**
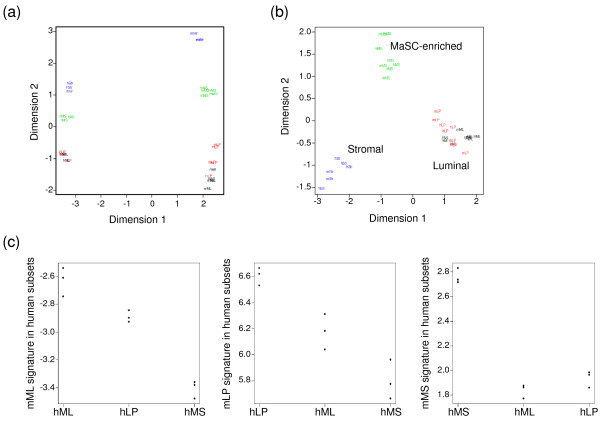
**Colocalization of gene signatures of corresponding human and mouse mammary cell subsets**. **(a) **Multidimension scaling (MDS) plot showing clear separation of the MaSC-enriched (MS), the two luminal (luminal progenitor, LP; and mature luminal, ML), and stromal (str) cell subpopulations in both human (h) and mouse (m) mammary glands. **(b) **MDS plot after normalization for dimension 1 in (a), representing the differences across species. This demonstrates colocalization of analogous human and mouse mammary cell subsets. **(c) **Dot plots demonstrating the highest colocalization of gene signatures between corresponding subsets compared with other subsets.

We previously reported the expression profiling of human mammary epithelial cell subpopulations, by using freshly sorted cells, unamplified material, and the Illumina platform [7]. The human and mouse gene expression profiles were first compared in a multidimension scaling (MDS) plot analysis, an unsupervised two-dimensional display of differences between profiles. As expected, samples separated clearly by species (Figure 3a). After normalizing for species differences, however, the samples clustered clearly by cell subtype (Figure 3b), showing that relative expression patterns across the cell subtypes are largely conserved between the two species. Dimension 1 distinguishes stromal cells from luminal cells, whereas dimension 2 distinguishes stem cells from others. The luminal progenitor and mature luminal subpopulations shared the greatest similarity, especially in the case of mouse.

To relate the two species more closely, we examined the transcriptional activity scores of the mouse subtype-specific signature genes in each of the human RNA samples. This demonstrated a conserved expression profile for each epithelial cell subtype across the two species. For each subpopulation, the mouse transcriptional signature was consistently highest in the corresponding human subtype for every biologic replicate (Figure 3c). The two luminal subtypes showed intermediate cross-over transcriptional activity with each other, whereas the transcriptional activity of the MaSC-enriched subset was relatively specific (Figure 3c).

### Conservation of signature genes between mouse and human mammary subpopulations

Next we looked for genes in common between the mouse and human signatures for each epithelial subpopulation. For this analysis, we used a more comprehensive set of signature genes, by loosening slightly the FDR criteria, as described in Methods (Tables S1-S3 in Additional files 4, 5, and 6). Of a total of 8,451 mouse probes that were signature probes for the three epithelial subpopulations, 4,758 unique mouse genes with human orthologues were found, of which 1,204 (25%) were found to be signature genes for the corresponding human subpopulation (Table 2). As expected, the MaSC-enriched subpopulation had the largest number of signature genes and the highest conservation rate between species, with 489 shared upregulated and 428 shared downregulated genes (Table 2), indicating strong conservation in basal lineage genes. The lower degree of conservation evident in the luminal progenitor and mature luminal cell signatures in part reflects the closer relation between these two subpopulations but also suggests that they may be more heterogeneous than previously anticipated.

**Table 2:** Number of mouse signature probes and genes conserved in human mammary epithelial cell subpopulations

Mammary epithelial cell subsets	Mouse probes	Unique mouse genes	Mouse genes with human orthologues	Mouse genes conserved in humans
**MaSC-enriched**				
Upregulated	2,811	1,928	1,551	489
Downregulated	2,474	1,729	1,395	428
**Luminal progenitor**				
Upregulated	604	435	333	58
Downregulated	203	152	128	14
**Mature luminal**				
Upregulated	1,112	784	606	116
Downregulated	1,247	889	745	99

Inclusive sets of signature probes were chosen by using "nestedF" with fold-change > 1.5 and FDR < 0.1.

The conserved upregulated genes in the MaSC-enriched population spanned diverse gene ontology groups, including transcription factors (for example, *Irx4, Mef2c, Slug, Egr2, Twist2, Tbx2, Id4, p63*, and *Sox11*), cytokeratins (*Krt5, 14, 16*), and plasma membrane proteins (for example, *Lgr6 *and the receptors for *Oxytocin, Oncostatin M*, and *Lif*). The *Notch *ligand *Jag2 *was highly expressed in this subpopulation, and its product may directly signal through Notch receptors expressed on adjacent luminal cells [28]. The Wnt/β-catenin pathway is anticipated to be active in self-renewing MaSCs, compatible with the observation that *Fzd8 *and *Tcf4 *are components of the conserved upregulated gene signature in the MaSC-enriched population. The Wnt-pathway inhibitors *Wif1 *and *Dkk3*, however, were also found to be abundantly expressed. These antagonists may be expressed and secreted by mature myoepithelial cells present within this population to attenuate Wnt signal transduction in the basally located MaSCs.

For the luminal progenitor signature, *Kit *(receptor tyrosine kinase), *Elf5 *(Ets transcription factor), *Cyp24A1 *(vitamin D metabolizing enzyme), *Lbp*, and *Cxcr4 *were highly expressed in both species. *Aldh1a3 *was also upregulated in luminal progenitor cells versus other cell types, although another isoform *Aldh5a1 *was identified in the luminal-restricted population isolated by Raouf *et al*. [11]. Interestingly, virtually all the ALDH activity in human breast epithelium resides within the luminal progenitor population (unpublished data) rather than the more primitive mammary stem cell subset [29]. In mature luminal cells, highly expressed genes included the transcription factors *Foxa1*, *Myb*, *estrogen receptor (ER)*, *progesterone receptor *(*PgR*), and *Tbx3*, as well as the *prolactin receptor *(*Prlr*) and *Rank ligand *(*Tnfsf11*).

**Figure 4:**
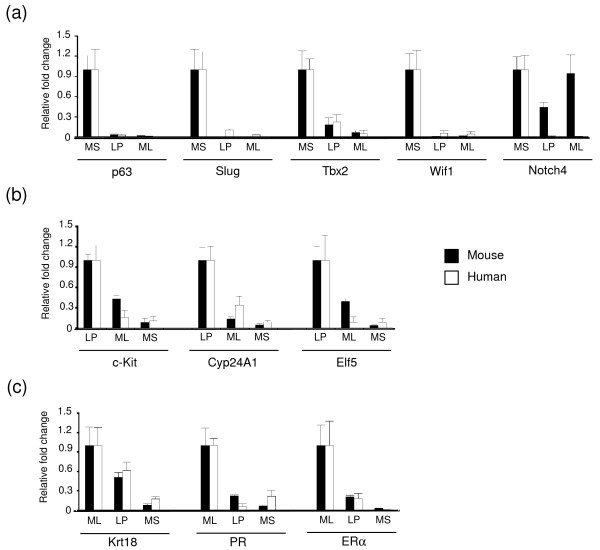
**Quantitative RT-PCR analysis of specific genes that define human and mouse mammary epithelial subsets**. Histograms depicting the relative fold difference in RNA expression between specific mammary epithelial cell subsets relative to other subsets in mouse and human mammary tissue. Expression analysis was relative to 18S rRNA. Examples of genes primarily expressed in the **(a) **MaSC-enriched subset, **(b) **luminal progenitor subset, and **(c) **mature luminal subset. At least three independent samples from either mouse or human mammary cell populations were evaluated for each gene. Data represent mean ± SEM. Statistically significant differences of *P *< 0.05 (*t *test) were observed between expression in the basal (MS) versus the two luminal subpopulations (ML and PL) for all genes except mouse *Notch4*.

Quantitative RT-PCR analysis was used to validate a number of genes in the conserved signatures, examples of which are shown in Figure 4. In the MaSC-enriched population, the Wnt inhibitory factor *Wif1 *and transcription factors *Δ Np63*, *Tbx2*, and *slug *(*snail2*), were preferentially expressed, thus validating the Illumina microarray data. Human *NOTCH-4 *was most highly expressed in the basal population, compatible with the findings of Raouf *et al*. [11] but differing from the mouse *Notch-4 *gene, which was expressed in all epithelial subsets at low levels [28]. *C-Kit*, *Cyp24A1*, and *Elf5 *were predominantly expressed in the luminal progenitor population in both species. Although low levels of *KIT *mRNA were evident in the human MaSC-enriched population, FACS analysis has shown that KIT protein is selectively expressed by human luminal progenitor cells (data not shown). As expected, *Krt18*, *ERα*, and *PgR *were preferentially expressed in mature luminal cells in both mouse and human, consistent with immunostaining of freshly sorted cells [7,12]. The differential expression of other genes, including *RankL*, *amphiregulin*, *Wnt4*, and *ErbB2 *was also confirmed in the mouse and human subsets (data not shown).

### Conservation of canonic pathways between mouse and human subpopulations

**Figure 5:**
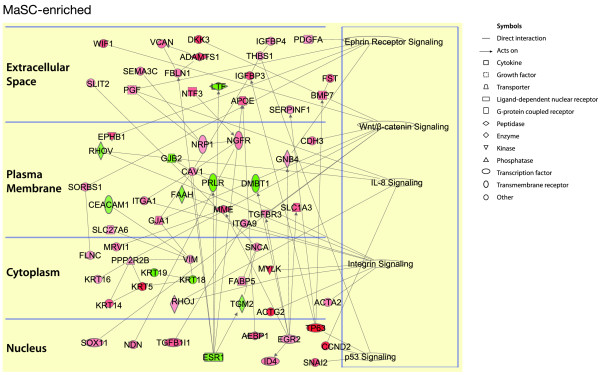
**Conserved genes and pathways across the human and mouse mammary stem cell-enriched subsets**. Ingenuity pathway analysis of conserved genes between mouse and human, and mouse genes with human orthologues, in the MaSC-enriched subset. The genes are divided according to the cellular distribution of the proteins for which they encode. The active pathways in each subset are represented on the right. Red represents upregulated genes, whereas green depicts downregulated genes. White symbols depict neighboring genes. The intensity of color represents the average log fold-change in a given population relative to the other epithelial subsets.

To identify pathways and gene networks active in both human and mouse, the conserved signature genes for each epithelial subpopulation were analyzed by using the Ingenuity Pathway Analysis (IPA) software [26]. For each subpopulation, canonic molecular pathways that had greatest overlap with the conserved signature genes were selected. The resulting pathways therefore center on conserved genes characteristic of the various epithelial populations. In the MaSC-enriched subset, several pathways were found to be conserved across species, forming a number of specific nodes that include the ephrin receptor, integrin, interleukin-8, p53, and Wnt/β-catenin signaling pathways (Figure 5). Interestingly, IL-8 has recently been implicated in the cancer stem cell signature of the ALDH^+ ^population in several breast cancer cell lines. IL-8 was also shown to enhance mammosphere formation and ALDH activity in these cell lines [30].

**Figure 6:**
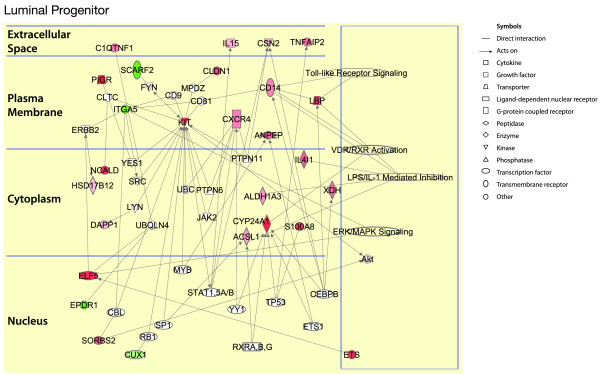
**Conserved genes and pathways across the human and mouse luminal progenitor cell subsets**. Ingenuity pathway analysis of conserved genes between mouse and human, and mouse genes with human orthologues, in the luminal progenitor subset. The genes are divided according to the cellular distribution of the proteins for which they encode. The active pathways in each subset are represented on the right. Red represents upregulated genes, whereas green depicts downregulated genes. White symbols depict neighboring genes. The intensity of color represents the average log fold-change in a given population relative to the other epithelial subsets.

**Figure 7:**
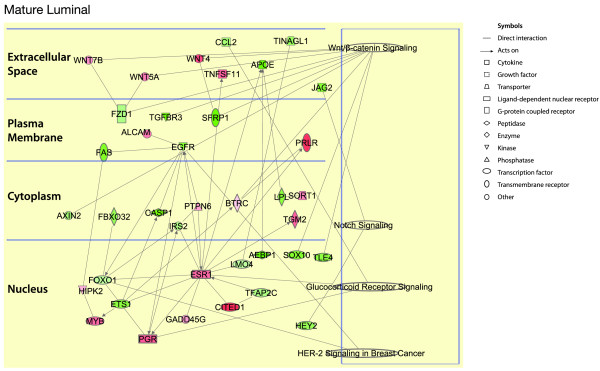
**Conserved genes and pathways across the human and mouse mature luminal cell subsets**. Ingenuity pathway analysis of conserved genes between mouse and human, and mouse genes with human orthologues, in the mature luminal epithelial cell subset. The genes are divided according to the cellular distribution of the proteins for which they encode. The active pathways in each subset are represented on the right. Red represents upregulated genes, whereas green depicts downregulated genes. White symbols depict neighboring genes. The intensity of color represents the average log fold-change in a given population relative to the other epithelial subsets.

For the luminal progenitor cell population, the network was expanded through the addition of neighboring genes (depicted in white, Figure 6), as few connections were evident. Conserved pathways include the Toll-like receptor, vitamin D receptor, and Erk/Mapk signaling pathways. Kit, Elf5, and Cyp24A1 represent highly differentially expressed genes that form key components of the luminal progenitor cell signature. In the mature luminal progenitor subset, the steroid hormone receptor, HER2/erbB2, and Notch signaling networks emerged as conserved pathways across species (Figure 7).

## Discussion

In this study, we describe a comparative transcriptome analysis of functionally analogous human and mouse mammary cell populations using an Illumina platform. Four prospectively isolated populations were evaluated, corresponding to those enriched for basal/mammary stem cells, committed luminal progenitor, mature luminal epithelial, and stromal cells. Distinct gene signatures were apparent for the mouse subpopulations, reminiscent of that found for human mammary cell subsets [7]. Comparison of the mammary epithelial signatures across human and mouse, combined with Ingenuity pathway analysis, revealed a number of conserved genes and pathways that are likely to regulate key processes during mammary ontogeny.

The MaSC-enriched subset exhibited the largest number of genes conserved across species. This subset comprises stem cells, likely basal progenitor cells, as well as mature myoepithelial cells. These cells share many common cell-surface markers that have impeded efforts to fractionate this population. Multiple transcriptional regulators (*Irx4, Mef2C, Slug, Egr2, Twist2, Tbx2*) were found to be highly expressed in this basal subset. Interestingly, the leucine-rich repeat-containing G protein-coupled receptor *Lgr6 *[31], which belongs to the same subgroup as Lgr5, a stem cell marker of small intestine, colon, and hair follicles [32], was identified as a component of the MaSC-gene signature. A prominent integrin network also emerged; these proteins play an important role in mediating interactions between basal cells (including MaSCs) and the underlying extracellular matrix. Of relevance, several genes attributed to cells that have undergone an EMT [33], such as slug, vimentin, and absence of E-cadherin expression, also characterize basal cells in the mammary gland. Therefore, the expression of these genes in breast tumor cells may indicate the acquisition of basal cell characteristics rather than an EMT. The recently described link between Wnt signaling and the EMT [34] may also reflect an active Wnt pathway in MaSCs or other cells in this basal population.

Kit, Cyp24A1, and Elf5 appear to be defining markers of committed luminal progenitor cells in both species. Interestingly, the tyrosine kinase KIT was reported to be overexpressed in basal breast cancers [35] and *BRCA1*-associated basal cancers [7], suggesting that it may serve as a useful prognostic marker or therapeutic target. Elf5 has been demonstrated to be important for driving alveolar cell differentiation during pregnancy [36] but may play an earlier role in regulating luminal cell-fate decisions. Interestingly, triple-negative breast cancer patients have been shown to have lower serum vitamin D levels, and Cyp24A1 is known to catabolize both 25-hydroxyvitamin D and 1,25-dihydroxyvitamin D. It is therefore tempting to speculate that higher levels of CYP24A1 might be linked to increased breast cancer risk [37]. Other interesting candidates include CXCR4, a receptor implicated in mediating metastasis of breast cancer cells through its ligand SDF-1 [38], and CD14 and lipopolysaccharide-binding protein (LBP), which are implicated in Toll-like receptor signaling and LPS-mediated inhibition. In the mature luminal population, active pathways identified by IPA include the Wnt ligands (Wnt4, 5A, 7B), which may act on MaSCs to enhance their self-renewal or proliferation. Expression of the transcriptional regulator *Lmo4 *was downregulated in the mature luminal subset, consistent with findings that this oncoprotein is important for promoting mammary epithelial cell proliferation and inhibiting differentiation [39,40]. Conversely, the expression of other transcriptional regulators (*ERα*, *Myb*, *PR*, and *Cited1*) was significantly upregulated in mature luminal cells.

A high degree of concordance was found between the expression profiles of the basal and mature luminal cell subsets in the mouse mammary gland described here and those previously reported [5,15], although the luminal progenitor expression profiles proved to be more divergent. For the basal/MaSC-enriched population, conserved pathways such as the Ephrin, Wnt, and extracellular matrix networks were also identified as nodes in interaction mapping of the basal subset by Kendrick *et al*. [15]. Similarly, the gene expression profiles of the mature luminal subset (reported here) shared substantial overlap with that of the ER^+ ^population described by Kendrick *et al*. [15], with the ER/glucocorticoid receptor signalling network emerging as one of the predominant nodes. The expression profile of the luminal progenitor subpopulation, however, exhibited substantial differences from that of the ER^- ^[15] and Ma-CFC subsets [5], indicating that they may represent distinct or heterogeneous cell populations. Nevertheless, the Kit and TLR signaling pathways identified here using Ingenuity Pathway analysis were also revealed as distinct modules in the ER^- ^network by ROCK analysis [15]. The gene profiles determined for the same three epithelial subsets isolated from human mammary tissue by Raouf *et al*. [11] show similarities but also differences that likely reflect short-term culture of their cells before gene expression studies [11].

Interrogation of breast cancer subtypes with the gene signatures of normal human epithelial cell subsets has revealed striking relations. Intriguingly, the luminal progenitor gene signature shared marked similarity with the basal subtype of breast cancer and preneoplastic breast tissue from *BRCA1 *mutation carriers [7]. Moreover, aberrant luminal progenitor cells were detected in *BRCA1 *mutation carriers, suggesting that they serve as a target population for further oncogenic events [7]. To extend these studies and identify candidate cell types that might contribute to oncogenesis in mouse models, we explored the link between the mouse mammary epithelial hierarchy and models of mammary tumorigenesis. The MaSC-enriched transcriptional signature was highest in *MMTV-Wnt-1 *and *p53*^-/- ^tumors, indicating that cells within these tumors exhibit similarities with MaSCs or basal progenitors. Although cancer stem cell populations have been identified in these tumors [41-43], one cannot conclude that these bear resemblance to MaSCs based on expression profiling studies. Rather, the molecular profiles may indicate a cell type that has been expanded during tumor progression. It is notable that preneoplastic tissue from *MMTV-Wnt-1 *transgenic mice has been shown to harbor an expanded mammary stem cell pool as well as aberrant bipotent progenitor cells, indicating that more than one cell of origin may exist in the *Wnt-1 *model [42].

The luminal progenitor signature was highest in *MMTV*-*Neu *and *MMTV-PyMT *tumors. Compatible with this observation for the *MMTV-Neu *model, FACS analyses of these tumors has indicated a homogeneous population of cells expressing high levels of the luminal progenitor marker CD61^+ ^[42]. Thus, luminal progenitor cells may have undergone expansion in these tumors. The *MMTV*-*Neu *strain, however, does not accurately recapitulate HER2-overexpressing cancers arising in women, because *MMTV-Neu *tumors do not show significant gene overlap with the HER2-positive subtype but are more similar to human "luminal" tumors [14]. Interestingly, the mature luminal signature was highest in *MMTV-Myc *tumors. The small progenitor subset in the 'mature' population [2,28], rather than the differentiated luminal cells, is likely to contribute to tumorigenesis in this model.

## Conclusions

The high degree of conservation between analogous epithelial subtypes across species supports the use of mice as a model system to study normal mammary gland development and oncogenesis. The conserved pathways pinpoint those that are likely to be involved in cell-fate decisions and lineage differentiation in the basal or luminal epithelial cell lineages. In the context of breast cancer, genes within the conserved signatures, such as those that characterize the more purified luminal progenitor subset (for example, *KIT*, *CYP24A1*, *ELF5*), have the potential to provide novel prognostic markers or therapeutic targets in breast cancer.

## Abbreviations

EMT: epithelial mesenchymal transition; IPA: Ingenuity Pathway Analysis; LP: luminal progenitor; MaSC: mammary stem cell; MDS: multidimension scaling; ML: mature luminal; MMTV: mouse mammary tumor virus; MS: MaSC-enriched; SEM: standard error of the mean; Str: stromal; TDLU: terminal ductal lobular unit.

## Competing interests

The authors declare that they have no competing interests.

## Authors' contributions

EL contributed to the conception and design, collection, assembly of data, and manuscript writing. DW and GS contributed to data analysis and interpretation and manuscript writing. BP, TB, MA, and FV contributed to the collection and assembly of data. HY provided clone HMbeta1-1 hybridoma to CD29 and advice. JEV and GJL contributed to the study conception, provision of study materials, data analysis, and manuscript writing. All authors read and approved the final manuscript.

## Supplementary Material

Additional file 1 **Supplementary methods**. This file contains primer sequences. Click here for file

Additional file 2 **Expression of CD24**. A figure demonstrating that CD24 is expressed in the luminal progenitor and mature luminal populations from human breast tissue. Click here for file

Additional file 3 **A heat map and multidimension scaling plot of mouse mammary populations**. Includes the MaSC-enriched (CD29^hi^CD24^+^), luminal progenitor (CD29^lo^CD24^+^CD61^+^), mature luminal (CD29^lo^CD24^+^CD61^-^), and stromal (CD29^lo^CD24^-^) subpopulations. Click here for file

Additional file 4 Supplementary Table 1 containing conserved genes in the MaSC-enriched subset. The table gives the 489 genes which are up-regulated and the 428 genes which are down-regulated in the MaSC-enriched subset in both species. Click here for file

Additional file 5 Supplementary Table 2 containing conserved genes in the luminal progenitor subset. The table gives the 58 genes which are up-regulated and the 14 genes which are down-regulated in the luminal progenitor subset in both species. Click here for file

Additional file 6 Supplementary Table 3 containing conserved genes in the mature luminal subset. The table gives the 116 genes which are up-regulated and the 99 genes which are down-regulated in the mature luminal subset in both species. Click here for file
