# Supplementary material for: Transcriptome analyses of mouse and human mammary cell subpopulations reveal multiple conserved genes and pathways
Source: Breast Cancer Res. 2010 Mar 26;12(2):R21. doi: 10.1186/bcr2560 (PMC2879567; doi:10.1186/bcr2560)
Supplement: Additional file 1 — Supplementary methods. This file contains primer sequences. [file bcr2560-S1.DOC]

**Supplementary Materials and Methods**

human TRP63 **F** 5’-ATTGCATCACTGTATCATTTTCT-3’

human TRP63 **R** 5’-TGCTCTGTGGGGACCTTTCA-3’

human SLUG **F** 5’-ATGAGGAATCTGGCTGCTGT-3’

human SLUG **R** 5’-CAGGAGAAAATGCCTTTGGA-3’

human TBX2 **F** 5’-GGCCGGCGGGAGAAAAGGAAG-3’

human TBX2 **R** 5’-GCACGACGAGGCGTCTGACT-3’

human WIF-1 **F** 5’-CATCCTCCTGTGCCTGCT-3’

human WIF-1 **R** 5’-TGGCATTCTCTGTTGTGCTT-3’

human NOTCH4 **F** 5’-GACCTACTTGCCACCTTCCA-3’

human NOTCH4 **R** 5’-AGGGAGAGCTTCTGCACTCA-3’

human KIT **F** 5’-CCACACCCTGTTCACTCCTT-3’

human KIT **R** 5’-TTCTGGGAAACTCCCATTTG-3’

human CYP24A-1 **F** 5’-GGTGACATCTACGGCGTACAC-3’

human CYP24A-1 **R** 5’-CTTGAGACCCCCTTTCCAGAG-3’

human ELF5 **F** 5’-ATGTTGGACTCGGTGACACA-3’

human ELF5 **R** 5’-CTTGTACTGGTCGCAGCAGA-3’

human KRT18 **F** 5’-ggcgaggactttaatcttgg-3’

human KRT18 **R** 5’-tgcctcagaactttggtgtc-3’

human PR **F** 5’-ggtccttggaggtcgaaaat-3’

human PR **R** 5’-tagggcttggctttcatttg-3’

human ERα **F** 5’-AAGAGCTGCCAGGCCTGCC-3’

human ERα **F** 5’-TTGGCAGCTCTCATGTCTCC-3’

mouse Trp63 **F** 5’-GGATGATTTGGCAAGTCTGA-3’

mouse Trp63 **R** 5’-ACTTGGGGTCCTCAGGAGAT-3’

mouse Slug **F** 5’-CACATTCGAACCCACACATT-3’

mouse Slug **R** 5’-TATTGCAGTGAGGGCAAGAG-3’

mouse Tbx2 **F** 5’-CTTCATCGCTGTCACTGCCTA-3’

mouse Tbx2 **R** 5’-GCCATGTCGCTCCGGCTTACA-3’

mouse Wif1 **F** 5’-CCACCTGAGGAGAGCTTGTACC-3’

mouse Wif1 **R** 5’-TGGCATTCTTTGTTGGGCTTTCC-3’

mouse Notch4 **F** 5’-GAGGACCTGGTTGAAGAATTGATC-3’

mouse Notch4 **R** 5’-TGCAGTTTTTCCCCTTTTATCC-3’

mouse c-Kit **F** 5’-atcccgactttgtcagatgg-3’

mouse c-Kit **R** 5’-tctttgccaaacaagggaag-3’

mouse Cyp24a-1 **F** 5’-CTCCCTATGGATGCAGTATGTATAGTG-3’

mouse Cyp24a-1 **R** 5’-TTTAAAAACGTTGTCAGTAGGTCATAACT-3’

mouse Elf5 **F** 5’-ccctgaatactggaccaagc-3’

mouse Elf5 **R** 5’-gctgcctcaatgaactcctc-3’

mouse Krt18 **F** 5’-GTCATACTGGGCAGGATGT-3’

mouse Krt18 **R** 5’-CAAGATCGAAGACCTGAGG-3’

mouse PR **F** 5’-GCTTGCATGATCTTGTGAAACAGC-3’

mouse PR **R** 5’-GGAAATTCCACAGCCAGTGTCC-3’

mouse ERα **F** 5’-ATGAAAGGCGGCATACGGAAAG-3’

mouse ERα **R** 5’-CACCCATTTCATTTCGGCCTTC-3’

18S **F** 5’-GTAACCCGTTGAACCCCATT-3’

18S **R** 5’-CCATCCAATCGGTAGTAGCG-3’
