# Supplementary material for: Transcriptome analyses of mouse and human mammary cell subpopulations reveal multiple conserved genes and pathways
Source: Breast Cancer Res. 2010 Mar 26;12(2):R21. doi: 10.1186/bcr2560 (PMC2879567; doi:10.1186/bcr2560)
Supplement: Additional file 2 — Expression of CD24. A figure demonstrating that CD24 is expressed in the luminal progenitor and mature luminal populations from human breast tissue. [file bcr2560-S2.PDF]

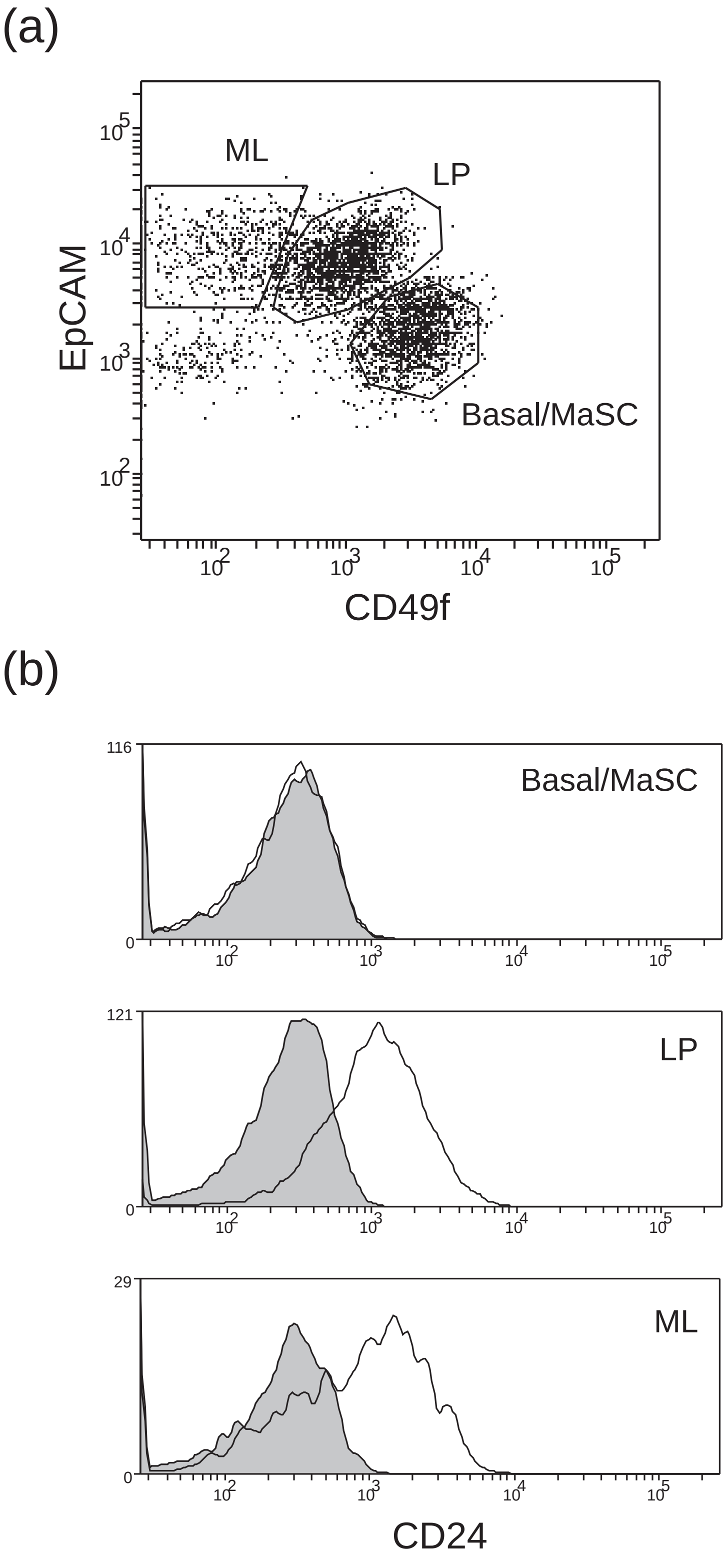

Supplementary Figure 1. **CD24 is expressed in the luminal progenitor and mature luminal populations. (a)** The Lin^{-} population ($CD31^{-}CD45^{-}CD235a^{-}$) was further depleted of stroma using the fibroblast-specific marker CD140b, as described by Lim et al [7]. A dot plot of the resultant human mammary epithelial subpopulations defined by CD49f and EpCAM is shown. **(b)** CD24 expression for each subpopulation (white histogram) compared to control (grey histogram), detected using an Alexa Fluor 647-conjugated anti-CD24 monoclonal antibody (Biolegend). Basal/MaSC, MaSC-enriched; LP, luminal progenitor; ML, mature luminal. Breast tissue was from the reduction mammoplasty specimen of a 33 year-old woman.
