# Supplementary material for: Transcriptome analyses of mouse and human mammary cell subpopulations reveal multiple conserved genes and pathways
Source: Breast Cancer Res. 2010 Mar 26;12(2):R21. doi: 10.1186/bcr2560 (PMC2879567; doi:10.1186/bcr2560)
Supplement: Additional file 3 — A heat map and multidimension scaling plot of mouse mammary populations. Includes the MaSC-enriched (CD29hiCD24+), luminal progenitor (CD29loCD24+CD61+), mature luminal (CD29loCD24+CD61-), and stromal (CD29loCD24-) subpopulations. [file bcr2560-S3.PDF]

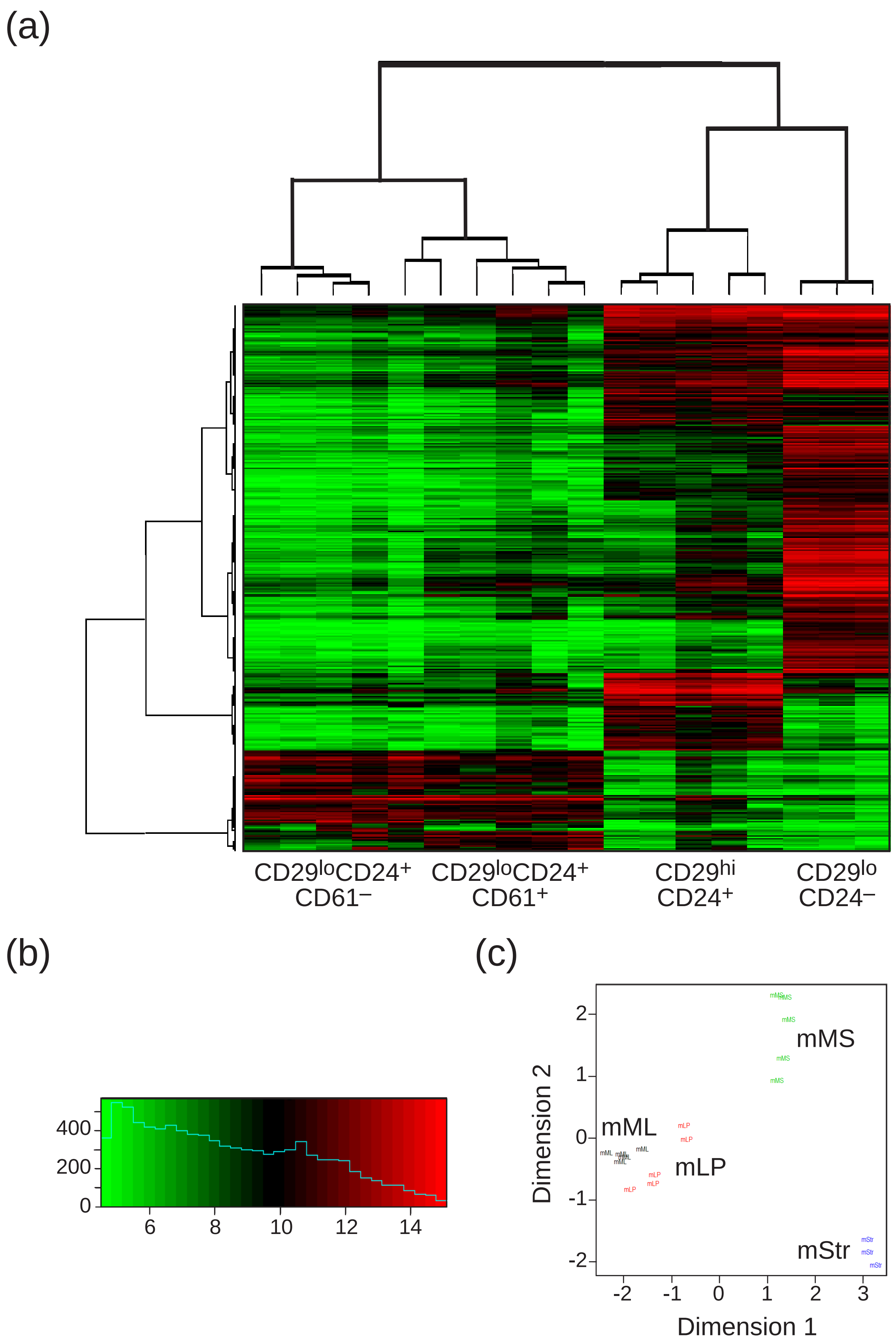

Supplementary Figure 2. The mouse mammary cell subpopulations defined by CD24, CD29 and CD61 have distinct gene expression profiles. (a) Hierarchical clustering and heatmap of the 500 genes most variable between the MaSC-enriched ($CD29^{hi}CD24^{+}$), luminal progenitor ($CD29^{lo}CD24^{+}CD61^{+}$), mature luminal ($CD29^{lo}CD24^{+}CD61^{-}$), and stromal ($CD29^{lo}CD24^{-}$) mammary cell subpopulations. **(b)** Color key and histogram of log-expression of genes in the heatmap. **(c)** Multidimension scaling plot showing clear separation of the MaSC-enriched (mMS), luminal progenitor (mLP), mature luminal (mML) and stromal (mStr) cell subpopulations.
