# Supplementary material for: Transcriptome analyses of mouse and human mammary cell subpopulations reveal multiple conserved genes and pathways
Source: Breast Cancer Res. 2010 Mar 26;12(2):R21. doi: 10.1186/bcr2560 (PMC2879567; doi:10.1186/bcr2560)
Supplement: Additional file 4 — Supplementary Table 1 containing conserved genes in the MaSC-enriched subset. The table gives the 489 genes which are up-regulated and the 428 genes which are down-regulated in the MaSC-enriched subset in both species. [file bcr2560-S4.DOC]

**Supplementary Table 1: Conserved genes in the MaSC-enriched subset**

| **Up-regulated in the MaSC-enriched subset** | | | | | | | |
| --- | --- | --- | --- | --- | --- | --- | --- |
| ID human | symbol | | log Fold Change | ID mouse | symbol | log Fold Change | average  log Fold  Change |
| ILMN_1651282 | COL17A1 | | 6.53 | ILMN_2879995 | Col17a1 | 5.16 | 5.84 |
| ILMN_2138801 | TP63 | | 6.00 | ILMN_1216742 | Trp63 | 5.20 | 5.60 |
| ILMN_1676822 | C2orf40 | | 6.03 | ILMN_1249000 | 1500015O10Rik | 4.90 | 5.47 |
| ILMN_2067656 | CCND2 | | 5.95 | ILMN_2700166 | Ccnd2 | 4.18 | 5.07 |
| ILMN_1676728 | DLK2 | | 5.26 | ILMN_2699621 | Dlk2 | 4.82 | 5.04 |
| ILMN_1801632 | KRT5 | | 4.47 | ILMN_2740939 | Krt5 | 5.60 | 5.04 |
| ILMN_1795325 | ACTG2 | | 4.28 | ILMN_2839313 | Actg2 | 5.79 | 5.03 |
| ILMN_1660086 | MYH11 | | 4.37 | ILMN_2622217 | Myh11 | 5.56 | 4.97 |
| ILMN_1772869 | ISM1 | | 5.35 | ILMN_2728134 | Ism1 | 4.44 | 4.90 |
| ILMN_1691476 | MYLK | | 5.14 | ILMN_1218347 | Mylk | 4.30 | 4.72 |
| ILMN_1725193 | IGFBP2 | | 3.91 | ILMN_2930897 | Igfbp2 | 5.45 | 4.68 |
| ILMN_1666502 | SOBP | | 4.13 | ILMN_2489448 | Sobp | 5.08 | 4.61 |
| ILMN_1670490 | PDPN | | 4.87 | ILMN_2654754 | Pdpn | 4.23 | 4.55 |
| ILMN_1696434 | LAMA1 | | 4.47 | ILMN_2973288 | Lama1 | 4.39 | 4.43 |
| ILMN_2398159 | DKK3 | | 4.68 | ILMN_2852957 | Dkk3 | 4.16 | 4.42 |
| ILMN_1652065 | KCNMB1 | | 4.67 | ILMN_1214965 | Kcnmb1 | 4.16 | 4.41 |
| ILMN_1766707 | IL17B | | 4.21 | ILMN_1252489 | Il17b | 4.50 | 4.36 |
| ILMN_1656248 | ASAM | | 5.50 | ILMN_2724194 | 9030425E11Rik | 2.98 | 4.24 |
| ILMN_1673566 | ADAMTS1 | | 4.66 | ILMN_2761082 | Adamts1 | 3.80 | 4.23 |
| ILMN_1684108 | IRX4 | | 3.42 | ILMN_2739872 | Irx4 | 4.92 | 4.17 |
| ILMN_1743373 | DLL1 | | 3.99 | ILMN_2721188 | Dll1 | 4.24 | 4.12 |
| ILMN_1675062 | MYL9 | | 3.15 | ILMN_2718662 | Myl9 | 5.02 | 4.08 |
| ILMN_1671928 | PROS1 | | 5.24 | ILMN_1235499 | Pros1 | 2.90 | 4.07 |
| ILMN_1748323 | CXCL14 | | 2.69 | ILMN_2760800 | Cxcl14 | 5.42 | 4.06 |
| ILMN_1751161 | COL7A1 | | 3.10 | ILMN_2901944 | Col7a1 | 4.97 | 4.04 |
| ILMN_1746085 | IGFBP3 | | 3.04 | ILMN_1219335 | Igfbp3 | 4.96 | 4.00 |
| ILMN_1692261 | EPHB1 | | 3.66 | ILMN_2672903 | Ephb1 | 4.30 | 3.98 |
| ILMN_1676088 | MSRB3 | | 5.72 | ILMN_1249215 | Msrb3 | 2.18 | 3.95 |
| ILMN_2082585 | SNAI2 | | 3.16 | ILMN_2647563 | Snai2 | 4.66 | 3.91 |
| ILMN_1789196 | TPM2 | | 3.16 | ILMN_2487170 | Tpm2 | 4.59 | 3.88 |
| ILMN_1796734 | SPARC | | 4.52 | ILMN_3136561 | Sparc | 3.16 | 3.84 |
| ILMN_2169761 | CPNE8 | | 4.08 | ILMN_3151149 | Cpne8 | 3.58 | 3.83 |
| ILMN_1808245 | C8orf84 | | 2.95 | ILMN_1244310 | Gm106 | 4.70 | 3.83 |
| ILMN_1721541 | WIF1 | | 3.23 | ILMN_2857748 | Wif1 | 4.42 | 3.83 |
| ILMN_1778668 | TAGLN | | 2.52 | ILMN_1243652 | Tagln | 5.13 | 3.82 |
| ILMN_2406035 | LAMA3 | | 3.62 | ILMN_2752545 | Lama3 | 4.01 | 3.81 |
| ILMN_1678170 | MME | | 5.13 | ILMN_2863362 | Mme | 2.45 | 3.79 |
| ILMN_1761322 | FHOD3 | | 4.30 | ILMN_2672221 | Fhod3 | 3.26 | 3.78 |
| ILMN_1658356 | PAMR1 | | 4.87 | ILMN_2774507 | Pamr1 | 2.60 | 3.73 |
| ILMN_1702363 | SULF1 | | 4.66 | ILMN_1222489 | Sulf1 | 2.70 | 3.68 |
| ILMN_1665035 | KRT14 | | 4.82 | ILMN_2722616 | Krt14 | 2.53 | 3.68 |
| ILMN_1736178 | AEBP1 | | 3.50 | ILMN_2873822 | Aebp1 | 3.80 | 3.65 |
| ILMN_1711120 | ARC | | 2.59 | ILMN_2597827 | Arc | 4.67 | 3.63 |
| ILMN_1724994 | COL4A2 | | 3.25 | ILMN_2822579 | Col4a2 | 3.94 | 3.59 |
| ILMN_1738552 | SLC1A3 | | 2.99 | ILMN_2634317 | Slc1a3 | 4.19 | 3.59 |
| ILMN_1700081 | FST | | 3.04 | ILMN_1228958 | Fst | 4.09 | 3.56 |
| ILMN_1656927 | SEMA5A | | 3.14 | ILMN_2604226 | Sema5a | 3.99 | 3.56 |
| ILMN_2389876 | TGFB1I1 | | 2.68 | ILMN_1227722 | Tgfb1i1 | 4.44 | 3.56 |
| ILMN_1740938 | APOE | | 3.67 | ILMN_1216042 | Apoe | 3.39 | 3.53 |
| ILMN_1777998 | ARHGAP25 | | 4.53 | ILMN_3155245 | Arhgap25 | 2.53 | 3.53 |
| ILMN_1786598 | COL14A1 | | 2.17 | ILMN_1229714 | Col14a1 | 4.89 | 3.53 |
| ILMN_1809364 | NTF3 | | 5.04 | ILMN_2760161 | Ntf3 | 1.98 | 3.51 |
| ILMN_1772612 | ANGPTL2 | | 3.79 | ILMN_1254653 | Angptl2 | 3.14 | 3.46 |
| ILMN_1737965 | ELOVL4 | | 2.05 | ILMN_1250507 | Elovl4 | 4.85 | 3.45 |
| ILMN_1810054 | CNN1 | | 5.49 | ILMN_1221148 | Cnn1 | 1.38 | 3.44 |
| ILMN_1746465 | FJX1 | | 2.95 | ILMN_2689998 | Fjx1 | 3.90 | 3.42 |
| ILMN_1804929 | OXTR | | 5.01 | ILMN_2543061 | Oxtr | 1.82 | 3.41 |
| ILMN_1655611 | TSHZ2 | | 3.50 | ILMN_1256408 | Tshz2 | 3.28 | 3.39 |
| ILMN_1668039 | GYPC | | 4.09 | ILMN_1218358 | Gypc | 2.64 | 3.36 |
| ILMN_1806733 | COL18A1 | | 2.86 | ILMN_2735184 | Col18a1 | 3.85 | 3.35 |
| ILMN_2189668 | NUDT11 | | 3.79 | ILMN_2615810 | Nudt11 | 2.89 | 3.34 |
| ILMN_1761968 | PPP1R14A | | 2.58 | ILMN_2659824 | Ppp1r14a | 4.06 | 3.32 |
| ILMN_2409642 | TRO | | 2.53 | ILMN_2510474 | Tro | 4.11 | 3.32 |
| ILMN_1653028 | COL4A1 | | 3.11 | ILMN_2621643 | Col4a1 | 3.50 | 3.30 |
| ILMN_1790761 | POSTN | | 4.13 | ILMN_2766028 | Postn | 2.47 | 3.30 |
| ILMN_1679262 | DPYSL3 | | 2.07 | ILMN_1250075 | Dpysl3 | 4.52 | 3.30 |
| ILMN_1692056 | HS3ST3A1 | | 2.54 | ILMN_2627193 | Hs3st3a1 | 4.05 | 3.30 |
| ILMN_1767448 | LHFP | | 2.61 | ILMN_2879588 | Lhfp | 3.98 | 3.29 |
| ILMN_1716370 | TNS4 | | 3.82 | ILMN_1236029 | Tns4 | 2.77 | 3.29 |
| ILMN_2324002 | CALD1 | | 2.75 | ILMN_2483771 | Cald1 | 3.81 | 3.28 |
| ILMN_1737252 | NRG1 | | 1.92 | ILMN_2971688 | Nrg1 | 4.60 | 3.26 |
| ILMN_1712075 | SYNM | | 2.64 | ILMN_1214880 | Synm | 3.87 | 3.26 |
| ILMN_1794598 | SCHIP1 | | 2.57 | ILMN_2937320 | Schip1 | 3.93 | 3.25 |
| ILMN_2094396 | LRRC8C | | 3.23 | ILMN_1248696 | Lrrc8c | 3.27 | 3.25 |
| ILMN_2076600 | ITM2A | | 4.38 | ILMN_2607377 | Itm2a | 2.09 | 3.24 |
| ILMN_2061435 | MEG3 | | 5.02 | ILMN_2523012 | Meg3 | 1.41 | 3.21 |
| ILMN_1755657 | RASIP1 | | 4.31 | ILMN_2740217 | Rasip1 | 2.12 | 3.21 |
| ILMN_1687301 | VCAN | | 2.32 | ILMN_2669627 | Vcan | 4.08 | 3.20 |
| ILMN_1679391 | MAMDC2 | | 3.85 | ILMN_2750558 | Mamdc2 | 2.53 | 3.19 |
| ILMN_1701461 | TIMP3 | | 2.79 | ILMN_2741096 | Timp3 | 3.53 | 3.16 |
| ILMN_1704294 | CDH3 | | 1.42 | ILMN_3156246 | Cdh3 | 4.89 | 3.15 |
| ILMN_1741566 | BMP7 | | 1.78 | ILMN_2589662 | Bmp7 | 4.53 | 3.15 |
| ILMN_1800642 | RELN | | 1.11 | ILMN_2704257 | Reln | 5.17 | 3.14 |
| ILMN_1662963 | PSD2 | | 1.72 | ILMN_2678366 | Psd2 | 4.51 | 3.11 |
| ILMN_2197128 | OSR1 | | 4.43 | ILMN_1259787 | Osr1 | 1.77 | 3.10 |
| ILMN_2336094 | ODZ3 | | 3.56 | ILMN_2741231 | Odz3 | 2.61 | 3.09 |
| ILMN_1789639 | FMOD | | 2.29 | ILMN_2999762 | Fmod | 3.86 | 3.07 |
| ILMN_1779875 | THY1 | | 4.13 | ILMN_2644350 | Thy1 | 1.97 | 3.05 |
| ILMN_1718295 | STAC2 | | 2.79 | ILMN_2703563 | Stac2 | 3.25 | 3.02 |
| ILMN_1801833 | ARHGAP24 | | 2.82 | ILMN_2728038 | Arhgap24 | 3.21 | 3.02 |
| ILMN_1658709 | LAMB1 | | 3.48 | ILMN_1253659 | Lamb1-1 | 2.52 | 3.00 |
| ILMN_1779147 | ENC1 | | 1.79 | ILMN_1237886 | Enc1 | 4.16 | 2.97 |
| ILMN_1651950 | TPST1 | | 2.58 | ILMN_1230765 | Tpst1 | 3.36 | 2.97 |
| ILMN_1752658 | NGFR | | 2.61 | ILMN_2851288 | Ngfr | 3.32 | 2.97 |
| ILMN_1721758 | ID4 | | 0.85 | ILMN_2678714 | Id4 | 5.06 | 2.96 |
| ILMN_1692058 | NDN | | 2.94 | ILMN_2622374 | Ndn | 2.96 | 2.95 |
| ILMN_1705066 | BTBD11 | | 3.69 | ILMN_3139253 | Btbd11 | 2.21 | 2.95 |
| ILMN_1727087 | GJA1 | | 3.65 | ILMN_1244291 | Gja1 | 2.23 | 2.94 |
| ILMN_1802411 | ITGA1 | | 2.90 | ILMN_2967445 | Itga1 | 2.97 | 2.93 |
| ILMN_1680948 | LMOD1 | | 2.66 | ILMN_2604885 | Lmod1 | 3.20 | 2.93 |
| ILMN_1672536 | FBLN1 | | 2.91 | ILMN_2870672 | Fbln1 | 2.89 | 2.90 |
| ILMN_1655405 | SCARF2 | | 4.33 | ILMN_2676127 | Scarf2 | 1.46 | 2.90 |
| ILMN_1695475 | SEMA3C | | 2.34 | ILMN_1238568 | Sema3c | 3.45 | 2.90 |
| ILMN_1766157 | MRVI1 | | 0.93 | ILMN_2746283 | Mrvi1 | 4.86 | 2.89 |
| ILMN_2104295 | TMEM178 | | 5.11 | ILMN_2650475 | Tmem178 | 0.67 | 2.89 |
| ILMN_1743933 | TSHZ3 | | 3.17 | ILMN_1253593 | Tshz3 | 2.57 | 2.87 |
| ILMN_1736670 | PPP1R3C | | 4.72 | ILMN_2667091 | Ppp1r3c | 1.02 | 2.87 |
| ILMN_1766925 | CDH13 | | 2.69 | ILMN_2592321 | Cdh13 | 3.02 | 2.85 |
| ILMN_1784287 | TGFBR3 | | 2.44 | ILMN_2789239 | Tgfbr3 | 3.24 | 2.84 |
| ILMN_1782419 | GNG11 | | 3.75 | ILMN_1251233 | Gng11 | 1.90 | 2.83 |
| ILMN_1780799 | ENPP2 | | 2.56 | ILMN_2954474 | Enpp2 | 3.02 | 2.79 |
| ILMN_1701877 | AXL | | 2.24 | ILMN_2651715 | Axl | 3.33 | 2.78 |
| ILMN_1676563 | HTRA1 | | 2.20 | ILMN_2746738 | Htra1 | 3.36 | 2.78 |
| ILMN_1671703 | ACTA2 | | 3.80 | ILMN_2693895 | Acta2 | 1.75 | 2.78 |
| ILMN_1672350 | JAM2 | | 3.07 | ILMN_1227559 | Jam2 | 2.48 | 2.77 |
| ILMN_1661708 | LGALS7 | | 1.95 | ILMN_2703182 | Lgals7 | 3.59 | 2.77 |
| ILMN_1773459 | SOX11 | | 2.28 | ILMN_1235647 | Sox11 | 3.25 | 2.77 |
| ILMN_1760247 | CD70 | | 2.20 | ILMN_2996338 | Cd70 | 3.29 | 2.74 |
| ILMN_1806403 | RASL12 | | 2.17 | ILMN_2836749 | Rasl12 | 3.31 | 2.74 |
| ILMN_1727360 | MAOB | | 2.47 | ILMN_2719069 | Maob | 2.99 | 2.73 |
| ILMN_1769575 | JAM3 | | 4.38 | ILMN_2633439 | Jam3 | 1.08 | 2.73 |
| ILMN_1701933 | SNCA | | 4.82 | ILMN_3136638 | Snca | 0.63 | 2.73 |
| ILMN_2188521 | PVRL3 | | 1.99 | ILMN_1250766 | Pvrl3 | 3.42 | 2.70 |
| ILMN_1670379 | ANTXR1 | | 1.73 | ILMN_1226183 | Antxr1 | 3.67 | 2.70 |
| ILMN_1742044 | GNAI1 | | 1.58 | ILMN_2592815 | Gnai1 | 3.80 | 2.69 |
| ILMN_2146761 | FABP5 | | 3.29 | ILMN_1235908 | Fabp5 | 2.08 | 2.68 |
| ILMN_1813117 | ITGA9 | | 1.85 | ILMN_2749529 | Itga9 | 3.50 | 2.68 |
| ILMN_1676413 | VSNL1 | | 2.09 | ILMN_2438724 | Vsnl1 | 3.26 | 2.68 |
| ILMN_1652413 | UCN2 | | 1.66 | ILMN_2425790 | Ucn2 | 3.69 | 2.67 |
| ILMN_1809813 | PGF | | 2.06 | ILMN_2768533 | Pgf | 3.29 | 2.67 |
| ILMN_1663446 | SORBS1 | | 1.64 | ILMN_3027751 | Sorbs1 | 3.69 | 2.67 |
| ILMN_1782761 | ARHGAP20 | | 2.27 | ILMN_2959729 | Arhgap20 | 3.03 | 2.65 |
| ILMN_1802109 | KLHL29 | | 3.61 | ILMN_1250099 | Klhl29 | 1.69 | 2.65 |
| ILMN_2149226 | CAV1 | | 3.93 | ILMN_2632665 | Cav1 | 1.34 | 2.63 |
| ILMN_2051972 | GPC3 | | 2.62 | ILMN_2832979 | Gpc3 | 2.64 | 2.63 |
| ILMN_1715068 | AQP9 | | 1.39 | ILMN_1214634 | Aqp9 | 3.78 | 2.59 |
| ILMN_1674344 | PARD6G | | 2.33 | ILMN_1237631 | Pard6g | 2.82 | 2.57 |
| ILMN_1723978 | LGALS1 | | 1.94 | ILMN_2619107 | Lgals1 | 3.20 | 2.57 |
| ILMN_2243036 | HAS2 | | 1.44 | ILMN_1254975 | Has2 | 3.69 | 2.57 |
| ILMN_1715991 | SDPR | | 2.41 | ILMN_2687547 | Sdpr | 2.72 | 2.57 |
| ILMN_1665865 | IGFBP4 | | 2.39 | ILMN_1258988 | Igfbp4 | 2.70 | 2.55 |
| ILMN_1792256 | TBX2 | | 2.11 | ILMN_2773918 | Tbx2 | 2.96 | 2.53 |
| ILMN_1742544 | MEF2C | | 1.78 | ILMN_2732465 | Mef2c | 3.29 | 2.53 |
| ILMN_1760493 | LIMS2 | | 2.23 | ILMN_2738345 | Lims2 | 2.84 | 2.53 |
| ILMN_1751465 | BNC1 | | 3.03 | ILMN_2910258 | Bnc1 | 2.03 | 2.53 |
| ILMN_1694840 | MATN2 | | 3.35 | ILMN_1230447 | Matn2 | 1.69 | 2.52 |
| ILMN_1764577 | MFNG | | 2.98 | ILMN_2687661 | Mfng | 2.05 | 2.51 |
| ILMN_1703913 | DST | | 4.13 | ILMN_2721385 | Dst | 0.89 | 2.51 |
| ILMN_1733157 | THSD1 | | 3.26 | ILMN_2759285 | Thsd1 | 1.76 | 2.51 |
| ILMN_1660282 | POPDC2 | | 0.96 | ILMN_3128351 | Popdc2 | 4.05 | 2.50 |
| ILMN_1751559 | TMEM204 | | 2.31 | ILMN_3162005 | Tmem204 | 2.65 | 2.48 |
| ILMN_1714586 | VGLL3 | | 2.59 | ILMN_1220029 | Vgll3 | 2.35 | 2.47 |
| ILMN_1721247 | KRT75 | | 3.28 | ILMN_2679759 | Krt75 | 1.65 | 2.47 |
| ILMN_1774685 | IL24 | | 3.24 | ILMN_1214841 | Il24 | 1.66 | 2.45 |
| ILMN_1685122 | COL9A2 | | 1.79 | ILMN_2945030 | Col9a2 | 3.10 | 2.44 |
| ILMN_1676449 | SLIT2 | | 1.12 | ILMN_1253797 | Slit2 | 3.74 | 2.43 |
| ILMN_2072101 | C4orf49 | | 3.57 | ILMN_1250001 | 4930583H14Rik | 1.23 | 2.40 |
| ILMN_1739496 | PRRX1 | | 3.06 | ILMN_2678094 | Prrx1 | 1.74 | 2.40 |
| ILMN_2052891 | PKD2 | | 2.82 | ILMN_2866327 | Pkd2 | 1.98 | 2.40 |
| ILMN_1657373 | LEPREL1 | | 3.14 | ILMN_1249635 | Leprel1 | 1.65 | 2.40 |
| ILMN_1756595 | SH3TC1 | | 3.00 | ILMN_2847906 | Sh3tc1 | 1.80 | 2.40 |
| ILMN_1745077 | MIA | | 3.33 | ILMN_2777087 | Mia1 | 1.43 | 2.38 |
| ILMN_1716264 | ANKRD1 | | 2.52 | ILMN_2950286 | Ankrd1 | 2.22 | 2.37 |
| ILMN_2315979 | LBH | | 3.27 | ILMN_2816180 | Lbh | 1.46 | 2.36 |
| ILMN_1671295 | CCDC3 | | 1.85 | ILMN_2611180 | Ccdc3 | 2.87 | 2.36 |
| ILMN_1733756 | COL12A1 | | 1.98 | ILMN_2862538 | Col12a1 | 2.74 | 2.36 |
| ILMN_2377199 | SLC27A6 | | 2.08 | ILMN_1230314 | Slc27a6 | 2.63 | 2.36 |
| ILMN_1678215 | RHOJ | | 2.56 | ILMN_2653567 | Rhoj | 2.14 | 2.35 |
| ILMN_1669362 | IGFBP6 | | 2.64 | ILMN_2689790 | Igfbp6 | 2.07 | 2.35 |
| ILMN_1775708 | SLC2A3 | | 1.57 | ILMN_2616565 | Slc2a3 | 3.12 | 2.35 |
| ILMN_1699574 | NRP1 | | 2.89 | ILMN_1237197 | Nrp1 | 1.77 | 2.33 |
| ILMN_1743199 | EGR2 | | 2.51 | ILMN_2623983 | Egr2 | 2.15 | 2.33 |
| ILMN_1763433 | TRIM9 | | 3.25 | ILMN_1221546 | Trim9 | 1.41 | 2.33 |
| ILMN_1769615 | FLRT2 | | 3.45 | ILMN_2926842 | Flrt2 | 1.19 | 2.32 |
| ILMN_2343278 | PPAP2A | | 2.05 | ILMN_2759079 | Ppap2a | 2.58 | 2.32 |
| ILMN_1755173 | PLEKHA4 | | 1.28 | ILMN_1243826 | Plekha4 | 3.35 | 2.31 |
| ILMN_1696048 | C13orf33 | | 1.96 | ILMN_1213736 | 6330406I15Rik | 2.66 | 2.31 |
| ILMN_2141482 | SERPINF1 | | 1.94 | ILMN_2639239 | Serpinf1 | 2.68 | 2.31 |
| ILMN_1807919 | TNS1 | | 1.36 | ILMN_1229315 | Tns1 | 3.25 | 2.31 |
| ILMN_1666665 | COL23A1 | | 1.35 | ILMN_2650447 | Col23a1 | 3.26 | 2.30 |
| ILMN_1699562 | EFCAB1 | | 2.00 | ILMN_2917280 | Efcab1 | 2.60 | 2.30 |
| ILMN_1796628 | ADAMTS2 | | 1.63 | ILMN_2729103 | Adamts2 | 2.93 | 2.28 |
| ILMN_1739222 | ETV5 | | 2.32 | ILMN_1235329 | Etv5 | 2.24 | 2.28 |
| ILMN_1844408 | PLXNA2 | | 2.18 | ILMN_2701815 | Plxna2 | 2.37 | 2.28 |
| ILMN_1681515 | CRLF1 | | 1.58 | ILMN_2747811 | Crlf1 | 2.97 | 2.28 |
| ILMN_2342695 | PDGFA | | 2.13 | ILMN_2424721 | Pdgfa | 2.39 | 2.26 |
| ILMN_1715748 | FLNC | | 3.41 | ILMN_2618364 | Flnc | 1.11 | 2.26 |
| ILMN_1695058 | SLC38A5 | | 0.79 | ILMN_2649773 | Slc38a5 | 3.72 | 2.26 |
| ILMN_2129545 | GNB4 | | 2.43 | ILMN_2726905 | Gnb4 | 2.08 | 2.26 |
| ILMN_2383611 | PTPRE | | 1.50 | ILMN_2826916 | Ptpre | 3.01 | 2.25 |
| ILMN_1665219 | LTBP4 | | 1.64 | ILMN_2829262 | Ltbp4 | 2.87 | 2.25 |
| ILMN_1714418 | FAM101B | | 2.22 | ILMN_1259764 | Fam101b | 2.27 | 2.24 |
| ILMN_1807689 | PKNOX2 | | 1.17 | ILMN_3135037 | Pknox2 | 3.32 | 2.24 |
| ILMN_1686116 | THBS1 | | 2.01 | ILMN_2659151 | Thbs1 | 2.46 | 2.24 |
| ILMN_1730504 | AGPAT4 | | 1.91 | ILMN_2960714 | Agpat4 | 2.56 | 2.24 |
| ILMN_1675268 | LRP4 | | 3.21 | ILMN_1244134 | Lrp4 | 1.26 | 2.24 |
| ILMN_1782538 | VIM | | 1.56 | ILMN_2451022 | Vim | 2.90 | 2.23 |
| ILMN_1685608 | NPTX2 | | 2.10 | ILMN_2620406 | Nptx2 | 2.36 | 2.23 |
| ILMN_1675507 | AKAP2 | | 2.62 | ILMN_1249197 | Akap2 | 1.83 | 2.23 |
| ILMN_1721901 | CTNNAL1 | | 1.92 | ILMN_1232261 | Ctnnal1 | 2.53 | 2.22 |
| ILMN_1702973 | FAM176A | | 2.39 | ILMN_2813577 | Fam176a | 2.04 | 2.21 |
| ILMN_1776936 | KANK4 | | 2.44 | ILMN_2655586 | Kank4 | 1.97 | 2.21 |
| ILMN_1660732 | PPP2R2B | | 1.14 | ILMN_1229427 | Ppp2r2b | 3.26 | 2.20 |
| ILMN_1732831 | CHST7 | | 2.48 | ILMN_1216374 | Chst7 | 1.92 | 2.20 |
| ILMN_1675797 | EPDR1 | | 2.95 | ILMN_1225494 | Epdr1 | 1.45 | 2.20 |
| ILMN_2228162 | KRT16 | | 2.48 | ILMN_1229813 | Krt16 | 1.92 | 2.20 |
| ILMN_1755822 | SYDE1 | | 2.37 | ILMN_2596183 | Syde1 | 2.00 | 2.18 |
| ILMN_1807169 | TINAGL1 | | 2.13 | ILMN_2976129 | Tinagl1 | 2.23 | 2.18 |
| ILMN_1666122 | HEG1 | | 3.41 | ILMN_2600022 | Heg1 | 0.94 | 2.17 |
| ILMN_2112638 | SVEP1 | | 3.13 | ILMN_2691951 | Svep1 | 1.21 | 2.17 |
| ILMN_1702301 | DOCK10 | | 2.44 | ILMN_2593143 | Dock10 | 1.89 | 2.16 |
| ILMN_2205963 | C10orf54 | | 2.35 | ILMN_2651297 | 4632428N05Rik | 1.97 | 2.16 |
| ILMN_1677092 | GEM | | 2.70 | ILMN_2667181 | Gem | 1.62 | 2.16 |
| ILMN_1809850 | RCN3 | | 0.93 | ILMN_2723718 | Rcn3 | 3.39 | 2.16 |
| ILMN_1805842 | FHL1 | | 2.50 | ILMN_2713285 | Fhl1 | 1.82 | 2.16 |
| ILMN_1660806 | CSRP2 | | 2.84 | ILMN_2721439 | Csrp2 | 1.46 | 2.15 |
| ILMN_1697220 | NT5E | | 0.78 | ILMN_2813830 | Nt5e | 3.50 | 2.14 |
| ILMN_1785141 | MICAL2 | | 1.90 | ILMN_2636004 | Mical2 | 2.36 | 2.13 |
| ILMN_1684554 | COL16A1 | | 2.06 | ILMN_1248099 | Col16a1 | 2.19 | 2.13 |
| ILMN_1789733 | CLIP3 | | 3.50 | ILMN_1222626 | Clip3 | 0.75 | 2.13 |
| ILMN_1775501 | IL1B | | 3.54 | ILMN_2777498 | Il1b | 0.71 | 2.13 |
| ILMN_1810844 | RARRES2 | | 2.05 | ILMN_3003130 | Rarres2 | 2.19 | 2.12 |
| ILMN_1666819 | PHLDB1 | | 2.09 | ILMN_2724371 | Phldb1 | 2.12 | 2.10 |
| ILMN_1709026 | C6orf145 | | 1.24 | ILMN_1219591 | 1300014I06Rik | 2.96 | 2.10 |
| ILMN_1679797 | ADARB1 | | 2.40 | ILMN_3105343 | Adarb1 | 1.78 | 2.09 |
| ILMN_1706505 | COL5A1 | | 1.36 | ILMN_2748402 | Col5a1 | 2.82 | 2.09 |
| ILMN_1709486 | SRPX | | 2.15 | ILMN_2629486 | Srpx | 2.02 | 2.09 |
| ILMN_2129234 | TMEM47 | | 2.22 | ILMN_3124787 | Tmem47 | 1.94 | 2.08 |
| ILMN_1813338 | LAG3 | | 1.03 | ILMN_2719811 | Lag3 | 3.13 | 2.08 |
| ILMN_1685275 | MCAM | | 1.68 | ILMN_2955919 | Mcam | 2.45 | 2.06 |
| ILMN_1760347 | SRGN | | 2.54 | ILMN_2751527 | Srgn | 1.58 | 2.06 |
| ILMN_1680251 | SORCS1 | | 1.62 | ILMN_2827617 | Sorcs1 | 2.50 | 2.06 |
| ILMN_1704753 | EPAS1 | | 2.00 | ILMN_3052632 | Epas1 | 2.11 | 2.05 |
| ILMN_1733333 | CALML3 | | 2.46 | ILMN_2592901 | Calml3 | 1.64 | 2.05 |
| ILMN_1791226 | NXN | | 2.60 | ILMN_2474011 | Nxn | 1.51 | 2.05 |
| ILMN_1683148 | PRICKLE2 | | 2.09 | ILMN_2497575 | Prickle2 | 2.01 | 2.05 |
| ILMN_1754126 | SH2D5 | | 3.22 | ILMN_2697719 | Sh2d5 | 0.87 | 2.04 |
| ILMN_1806787 | CSDC2 | | 2.01 | ILMN_2736847 | Csdc2 | 2.05 | 2.03 |
| ILMN_1762106 | MMP2 | | 0.70 | ILMN_2678218 | Mmp2 | 3.34 | 2.02 |
| ILMN_2115125 | CTGF | | 1.07 | ILMN_2909150 | Ctgf | 2.98 | 2.02 |
| ILMN_1663454 | PKP1 | | 2.06 | ILMN_2762109 | Pkp1 | 1.98 | 2.02 |
| ILMN_1814629 | ZC3H12B | | 1.23 | ILMN_2516710 | Zc3h12b | 2.81 | 2.02 |
| ILMN_1716608 | NGF | | 2.93 | ILMN_2937596 | Ngf | 1.09 | 2.01 |
| ILMN_1659106 | PHLDA3 | | 1.23 | ILMN_2923607 | Phlda3 | 2.79 | 2.01 |
| ILMN_1675331 | PEG3 | | 3.23 | ILMN_1245246 | Peg3 | 0.76 | 2.00 |
| ILMN_1727671 | SSH1 | | 3.09 | ILMN_2445100 | Ssh1 | 0.90 | 2.00 |
| ILMN_1787548 | HSPG2 | | 1.02 | ILMN_2685329 | Hspg2 | 2.97 | 2.00 |
| ILMN_2122103 | ETS1 | | 2.19 | ILMN_3157483 | Ets1 | 1.79 | 1.99 |
| ILMN_2150402 | TMEM64 | | 2.42 | ILMN_2517060 | Tmem64 | 1.57 | 1.99 |
| ILMN_2274306 | NRP2 | | 2.45 | ILMN_2480957 | Nrp2 | 1.53 | 1.99 |
| ILMN_1774127 | STAC | | 1.75 | ILMN_1245844 | Stac | 2.21 | 1.98 |
| ILMN_1698885 | PTPRT | | 3.00 | ILMN_1239565 | Ptprt | 0.97 | 1.98 |
| ILMN_1699651 | IL6 | | 1.75 | ILMN_1243601 | Il6 | 2.17 | 1.96 |
| ILMN_2133996 | SHE | | 2.55 | ILMN_2757019 | She | 1.36 | 1.96 |
| ILMN_1785618 | SMTN | | 0.84 | ILMN_2695181 | Smtn | 3.06 | 1.95 |
| ILMN_1725791 | PTPLA | | 1.25 | ILMN_2734712 | Ptpla | 2.61 | 1.93 |
| ILMN_1741356 | PRICKLE1 | | 1.92 | ILMN_1228245 | Prickle1 | 1.94 | 1.93 |
| ILMN_1739640 | DCHS1 | | 1.55 | ILMN_1216341 | Dchs1 | 2.30 | 1.92 |
| ILMN_1748890 | GPR176 | | 1.81 | ILMN_2742912 | Gpr176 | 2.03 | 1.92 |
| ILMN_1695947 | SCN4B | | 1.54 | ILMN_2813547 | Scn4b | 2.31 | 1.92 |
| ILMN_1691376 | JAG1 | | 2.78 | ILMN_1257077 | Jag1 | 1.06 | 1.92 |
| ILMN_1792389 | RNF165 | | 2.79 | ILMN_2590554 | Rnf165 | 1.04 | 1.92 |
| ILMN_1654563 | EFNB1 | | 2.43 | ILMN_2698443 | Efnb1 | 1.40 | 1.92 |
| ILMN_1760849 | NETO2 | | 2.74 | ILMN_1245531 | Neto2 | 1.10 | 1.92 |
| ILMN_1674629 | C9orf3 | | 2.00 | ILMN_2838372 | 2010111I01Rik | 1.82 | 1.91 |
| ILMN_1710544 | PCDH7 | | 1.14 | ILMN_2881857 | Pcdh7 | 2.68 | 1.91 |
| ILMN_1723481 | CHST3 | | 1.58 | ILMN_2864172 | Chst3 | 2.22 | 1.90 |
| ILMN_2075603 | MRGPRF | | 1.22 | ILMN_2760254 | Mrgprf | 2.57 | 1.90 |
| ILMN_2167426 | ASPHD2 | | 1.84 | ILMN_3162247 | Asphd2 | 1.94 | 1.89 |
| ILMN_1798256 | UPP1 | | 1.90 | ILMN_2959291 | Upp1 | 1.88 | 1.89 |
| ILMN_1729117 | COL5A2 | | 1.63 | ILMN_2802687 | Col5a2 | 2.13 | 1.88 |
| ILMN_1670305 | SERPING1 | | 1.75 | ILMN_2913166 | Serping1 | 2.00 | 1.88 |
| ILMN_2323087 | RBPMS | | 0.85 | ILMN_3026557 | Rbpms | 2.90 | 1.88 |
| ILMN_2061565 | PLCH2 | | 2.05 | ILMN_2925433 | Plch2 | 1.70 | 1.87 |
| ILMN_1797372 | C3orf58 | | 1.67 | ILMN_2524691 | 1190002N15Rik | 2.06 | 1.87 |
| ILMN_1703695 | C19orf12 | | 0.72 | ILMN_2773909 | 1600014C10Rik | 2.99 | 1.86 |
| ILMN_1669772 | LRP1 | | 1.48 | ILMN_1237723 | Lrp1 | 2.23 | 1.85 |
| ILMN_2357855 | NTRK2 | | 2.15 | ILMN_3138904 | Ntrk2 | 1.55 | 1.85 |
| ILMN_1720511 | LRRN1 | | 1.39 | ILMN_2759736 | Lrrn1 | 2.27 | 1.83 |
| ILMN_1751028 | SERPINH1 | | 1.91 | ILMN_2777359 | Serpinh1 | 1.74 | 1.83 |
| ILMN_1772627 | D4S234E | | 2.89 | ILMN_2626345 | Nsg1 | 0.75 | 1.82 |
| ILMN_1729216 | CRYAB | | 2.51 | ILMN_2840213 | Cryab | 1.12 | 1.81 |
| ILMN_1695829 | TTYH2 | | 1.33 | ILMN_2491859 | Ttyh2 | 2.29 | 1.81 |
| ILMN_1691413 | NNAT | | 2.69 | ILMN_1241164 | Nnat | 0.93 | 1.81 |
| ILMN_1696699 | FAM184A | | 2.56 | ILMN_1232026 | Fam184a | 1.05 | 1.81 |
| ILMN_1749006 | RCSD1 | | 0.81 | ILMN_1250704 | Rcsd1 | 2.80 | 1.81 |
| ILMN_1814194 | TCF4 | | 2.35 | ILMN_2636463 | Tcf4 | 1.26 | 1.80 |
| ILMN_1664362 | SGIP1 | | 2.26 | ILMN_2631934 | Sgip1 | 1.35 | 1.80 |
| ILMN_1715508 | NNMT | | 1.80 | ILMN_2885277 | Nnmt | 1.79 | 1.80 |
| ILMN_1693004 | C20orf117 | | 1.18 | ILMN_2627140 | 9830001H06Rik | 2.41 | 1.79 |
| ILMN_1741755 | TRIM29 | | 1.20 | ILMN_1218207 | Trim29 | 2.39 | 1.79 |
| ILMN_1720606 | LRCH2 | | 2.07 | ILMN_1215518 | Lrch2 | 1.51 | 1.79 |
| ILMN_1801223 | WTIP | | 2.10 | ILMN_2424268 | Wtip | 1.47 | 1.79 |
| ILMN_1707649 | MPDZ | | 1.30 | ILMN_2662776 | Mpdz | 2.27 | 1.78 |
| ILMN_2207505 | LEP | | 0.85 | ILMN_2695964 | Lep | 2.70 | 1.77 |
| ILMN_1727156 | ARSI | | 1.33 | ILMN_2472093 | Arsi | 2.20 | 1.76 |
| ILMN_1722809 | NRCAM | | 2.59 | ILMN_1245780 | Nrcam | 0.93 | 1.76 |
| ILMN_1693009 | FGL2 | | 1.64 | ILMN_2654554 | Fgl2 | 1.86 | 1.75 |
| ILMN_2359287 | ITGA6 | | 1.84 | ILMN_1231492 | Itga6 | 1.64 | 1.74 |
| ILMN_1796377 | C14orf37 | | 1.20 | ILMN_2753589 | 3632451O06Rik | 2.28 | 1.74 |
| ILMN_1729368 | FZD8 | | 2.40 | ILMN_2597578 | Fzd8 | 1.07 | 1.74 |
| ILMN_1777397 | MSX1 | | 1.90 | ILMN_1246173 | Msx1 | 1.55 | 1.72 |
| ILMN_1687023 | GJC1 | | 1.02 | ILMN_2752275 | Gjc1 | 2.41 | 1.72 |
| ILMN_1713550 | LGR6 | | 1.43 | ILMN_2609504 | Lgr6 | 1.99 | 1.71 |
| ILMN_1680054 | LAMB3 | | 0.78 | ILMN_2605512 | Lamb3 | 2.64 | 1.71 |
| ILMN_1751904 | EDNRB | | 1.45 | ILMN_2589640 | Ednrb | 1.96 | 1.70 |
| ILMN_1688630 | RECK | | 1.42 | ILMN_2812614 | Reck | 1.98 | 1.70 |
| ILMN_1663976 | PDLIM4 | | 2.33 | ILMN_2606162 | Pdlim4 | 1.07 | 1.70 |
| ILMN_1761820 | EDARADD | | 1.60 | ILMN_2507540 | Edaradd | 1.79 | 1.70 |
| ILMN_2041788 | PLS3 | | 2.25 | ILMN_2764781 | Pls3 | 1.12 | 1.68 |
| ILMN_2182148 | CNRIP1 | | 1.73 | ILMN_1231506 | Cnrip1 | 1.61 | 1.67 |
| ILMN_1676709 | SDK2 | | 1.58 | ILMN_1221064 | Sdk2 | 1.75 | 1.66 |
| ILMN_1808132 | FAS | | 1.60 | ILMN_2902979 | Fas | 1.71 | 1.66 |
| ILMN_1799600 | STARD8 | | 1.50 | ILMN_1257855 | Stard8 | 1.80 | 1.65 |
| ILMN_2129927 | EXT1 | | 1.45 | ILMN_2731989 | Ext1 | 1.85 | 1.65 |
| ILMN_1814985 | PDLIM7 | | 0.91 | ILMN_2430813 | Pdlim7 | 2.39 | 1.65 |
| ILMN_1806349 | SLC6A8 | | 1.78 | ILMN_2605212 | Slc6a8 | 1.51 | 1.65 |
| ILMN_1707070 | PCOLCE | | 1.48 | ILMN_1253741 | Pcolce | 1.81 | 1.65 |
| ILMN_1655077 | PRDM1 | | 2.18 | ILMN_2597923 | Prdm1 | 1.10 | 1.64 |
| ILMN_1808374 | SNTB2 | | 1.97 | ILMN_1248791 | Sntb2 | 1.29 | 1.63 |
| ILMN_1654735 | SLCO3A1 | | 1.63 | ILMN_2663230 | Slco3a1 | 1.61 | 1.62 |
| ILMN_1753910 | CRISPLD1 | | 1.44 | ILMN_1230529 | Crispld1 | 1.80 | 1.62 |
| ILMN_1705210 | GRASP | | 1.70 | ILMN_2596396 | Grasp | 1.54 | 1.62 |
| ILMN_1758128 | CYGB | | 1.52 | ILMN_2801891 | Cygb | 1.71 | 1.62 |
| ILMN_1725271 | GPR3 | | 1.78 | ILMN_2678530 | Gpr3 | 1.45 | 1.61 |
| ILMN_1744403 | KCNIP3 | | 0.97 | ILMN_2911729 | Kcnip3 | 2.26 | 1.61 |
| ILMN_1788886 | TOX | | 0.94 | ILMN_1257547 | Tox | 2.25 | 1.60 |
| ILMN_1769891 | CDH4 | | 2.07 | ILMN_1260036 | Cdh4 | 1.12 | 1.60 |
| ILMN_1736539 | ALDH1L2 | | 0.79 | ILMN_2898319 | Aldh1l2 | 2.38 | 1.59 |
| ILMN_1792014 | FAM70B | | 1.30 | ILMN_2533140 | Fam70b | 1.87 | 1.59 |
| ILMN_1704369 | LIMA1 | | 2.14 | ILMN_1228374 | Lima1 | 1.03 | 1.58 |
| ILMN_1677429 | TWIST2 | | 1.42 | ILMN_1230558 | Twist2 | 1.73 | 1.57 |
| ILMN_1782581 | DUSP7 | | 2.22 | ILMN_2804523 | Dusp7 | 0.92 | 1.57 |
| ILMN_2086981 | POU3F1 | | 1.26 | ILMN_1233172 | Pou3f1 | 1.87 | 1.56 |
| ILMN_1674380 | TRPC1 | | 1.44 | ILMN_1257053 | Trpc1 | 1.68 | 1.56 |
| ILMN_1680132 | CADM1 | | 1.24 | ILMN_2505841 | Cadm1 | 1.88 | 1.56 |
| ILMN_1673639 | ABI3BP | | 1.70 | ILMN_3132588 | Abi3bp | 1.42 | 1.56 |
| ILMN_2360415 | PRNP | | 1.18 | ILMN_2619316 | Prnp | 1.94 | 1.56 |
| ILMN_1709307 | GPSM1 | | 0.92 | ILMN_1243154 | Gpsm1 | 2.20 | 1.56 |
| ILMN_1689237 | BVES | | 2.05 | ILMN_1222934 | Bves | 1.07 | 1.56 |
| ILMN_1707720 | SLC1A5 | | 1.96 | ILMN_2776585 | Slc1a5 | 1.14 | 1.55 |
| ILMN_1811313 | SLIT3 | | 1.18 | ILMN_2643545 | Slit3 | 1.91 | 1.54 |
| ILMN_1770084 | TACC1 | | 2.17 | ILMN_3126933 | Tacc1 | 0.91 | 1.54 |
| ILMN_1740508 | KCNMA1 | | 1.62 | ILMN_1256814 | Kcnma1 | 1.43 | 1.53 |
| ILMN_1712046 | CPXM1 | | 1.34 | ILMN_2645816 | Cpxm1 | 1.69 | 1.51 |
| ILMN_1701195 | PLA2G7 | | 1.13 | ILMN_2692696 | Pla2g7 | 1.87 | 1.50 |
| ILMN_1699489 | TUBB6 | | 1.77 | ILMN_2718217 | Tubb6 | 1.23 | 1.50 |
| ILMN_2229877 | PCDH18 | | 1.78 | ILMN_2729113 | Pcdh18 | 1.20 | 1.49 |
| ILMN_2278590 | MEST | | 0.67 | ILMN_2846904 | Mest | 2.30 | 1.49 |
| ILMN_1690454 | C3orf54 | | 2.10 | ILMN_2661799 | 6230427J02Rik | 0.86 | 1.48 |
| ILMN_1671046 | HSPB2 | | 1.72 | ILMN_2613904 | Hspb2 | 1.24 | 1.48 |
| ILMN_2195462 | C1QTNF4 | | 1.97 | ILMN_1252584 | C1qtnf4 | 0.98 | 1.48 |
| ILMN_2180371 | C12orf24 | | 1.97 | ILMN_2973024 | 1500011H22Rik | 0.96 | 1.47 |
| ILMN_1719627 | SLC27A3 | | 0.95 | ILMN_2747857 | Slc27a3 | 1.97 | 1.46 |
| ILMN_1727098 | PPP1R16B | | 1.71 | ILMN_2522495 | Ppp1r16b | 1.21 | 1.46 |
| ILMN_1664434 | TCF3 | | 1.04 | ILMN_3109289 | Tcf3 | 1.88 | 1.46 |
| ILMN_1756935 | OSBPL6 | | 1.36 | ILMN_1221789 | Osbpl6 | 1.55 | 1.46 |
| ILMN_1813175 | LPHN1 | | 1.60 | ILMN_1228782 | Lphn1 | 1.31 | 1.45 |
| ILMN_1754795 | CD36 | | 1.55 | ILMN_1232470 | Cd36 | 1.33 | 1.44 |
| ILMN_1764158 | NLGN2 | | 1.62 | ILMN_2972478 | Nlgn2 | 1.27 | 1.44 |
| ILMN_1662949 | FAM132A | | 1.29 | ILMN_2645295 | Fam132a | 1.58 | 1.44 |
| ILMN_1728858 | EGFR | | 1.01 | ILMN_2693922 | Egfr | 1.86 | 1.44 |
| ILMN_1743714 | CARD10 | | 1.64 | ILMN_2606660 | Card10 | 1.23 | 1.44 |
| ILMN_1773485 | QKI | | 1.10 | ILMN_1259609 | Qk | 1.74 | 1.42 |
| ILMN_2405009 | NBL1 | | 0.86 | ILMN_2886618 | Nbl1 | 1.98 | 1.42 |
| ILMN_1722781 | EGR3 | | 1.16 | ILMN_1227494 | Egr3 | 1.67 | 1.41 |
| ILMN_1709094 | LIFR | | 1.84 | ILMN_2829008 | Lifr | 0.98 | 1.41 |
| ILMN_1727134 | KLHDC5 | | 1.33 | ILMN_2522542 | Klhdc5 | 1.46 | 1.40 |
| ILMN_2357134 | SPHK1 | | 1.86 | ILMN_1232884 | Sphk1 | 0.93 | 1.40 |
| ILMN_2136455 | C3orf64 | | 1.36 | ILMN_2650183 | A130022J15Rik | 1.42 | 1.39 |
| ILMN_2339028 | PKD1 | | 1.14 | ILMN_2827036 | Pkd1 | 1.63 | 1.39 |
| ILMN_1812226 | ICAM1 | | 1.31 | ILMN_2896601 | Icam1 | 1.46 | 1.38 |
| ILMN_1735743 | FBLN7 | | 1.34 | ILMN_2637357 | Fbln7 | 1.42 | 1.38 |
| ILMN_1664294 | LEPRE1 | | 0.98 | ILMN_3150634 | Lepre1 | 1.77 | 1.38 |
| ILMN_1815102 | LCAT | | 1.17 | ILMN_2798402 | Lcat | 1.58 | 1.38 |
| ILMN_1795344 | GOLIM4 | | 1.69 | ILMN_1258813 | Golim4 | 1.06 | 1.37 |
| ILMN_1795865 | FGFRL1 | | 1.15 | ILMN_2621433 | Fgfrl1 | 1.60 | 1.37 |
| ILMN_1707232 | EBF3 | | 0.89 | ILMN_2422615 | Ebf3 | 1.86 | 1.37 |
| ILMN_1692335 | ELK3 | | 0.98 | ILMN_3119014 | Elk3 | 1.77 | 1.37 |
| ILMN_2334210 | ITGB4 | | 2.07 | ILMN_2634689 | Itgb4 | 0.67 | 1.37 |
| ILMN_1791006 | AHI1 | | 1.46 | ILMN_2813712 | Ahi1 | 1.26 | 1.36 |
| ILMN_1738420 | TMEM201 | | 2.03 | ILMN_2606973 | Tmem201 | 0.69 | 1.36 |
| ILMN_1666894 | CSPG4 | | 1.84 | ILMN_2659224 | Cspg4 | 0.87 | 1.36 |
| ILMN_2359627 | BCL2L11 | | 1.72 | ILMN_3157568 | Bcl2l11 | 1.00 | 1.36 |
| ILMN_1786648 | PRX | | 1.52 | ILMN_3154269 | Prx | 1.19 | 1.35 |
| ILMN_1743836 | MXRA7 | | 1.30 | ILMN_2702193 | Mxra7 | 1.40 | 1.35 |
| ILMN_1810852 | LAMC1 | | 1.14 | ILMN_2774596 | Lamc1 | 1.56 | 1.35 |
| ILMN_1811758 | CNP | | 1.47 | ILMN_2802263 | Cnp | 1.22 | 1.35 |
| ILMN_1723145 | TMEM121 | | 1.07 | ILMN_1231884 | Tmem121 | 1.60 | 1.34 |
| ILMN_1711608 | SSBP2 | | 1.16 | ILMN_2693461 | Ssbp2 | 1.52 | 1.34 |
| ILMN_1657332 | CCDC85B | | 0.76 | ILMN_1220846 | Ccdc85b | 1.90 | 1.33 |
| ILMN_1714159 | LUZP1 | | 1.78 | ILMN_2942276 | Luzp1 | 0.88 | 1.33 |
| ILMN_1720941 | EVC | | 0.81 | ILMN_2706562 | Evc | 1.82 | 1.31 |
| ILMN_2396287 | RFX2 | | 1.97 | ILMN_1217353 | Rfx2 | 0.66 | 1.31 |
| ILMN_2054297 | PTGS2 | | 1.63 | ILMN_1231600 | Ptgs2 | 1.00 | 1.31 |
| ILMN_2152711 | ACVR2A | | 1.37 | ILMN_2617775 | Acvr2a | 1.24 | 1.31 |
| ILMN_1688295 | ZNF219 | | 1.91 | ILMN_2896229 | Zfp219 | 0.70 | 1.31 |
| ILMN_1672608 | PPAP2B | | 1.00 | ILMN_2630993 | Ppap2b | 1.61 | 1.30 |
| ILMN_1653274 | NUDT10 | | 1.24 | ILMN_2974709 | Nudt10 | 1.36 | 1.30 |
| ILMN_1737360 | TSPYL3 | | 1.66 | ILMN_2965132 | Tspyl3 | 0.94 | 1.30 |
| ILMN_1668417 | WIPF1 | | 1.18 | ILMN_1257209 | Wipf1 | 1.41 | 1.29 |
| ILMN_2110908 | MYC | | 1.15 | ILMN_2623526 | Myc | 1.42 | 1.29 |
| ILMN_1740407 | CHSY3 | | 1.83 | ILMN_1216568 | Chsy3 | 0.73 | 1.28 |
| ILMN_1815690 | TIE1 | | 1.00 | ILMN_2928560 | Tie1 | 1.55 | 1.28 |
| ILMN_1656422 | GPR87 | | 0.81 | ILMN_2802874 | Gpr87 | 1.72 | 1.27 |
| ILMN_1776000 | EID3 | | 1.31 | ILMN_1254327 | Eid3 | 1.23 | 1.27 |
| ILMN_1797861 | IL6ST | | 1.24 | ILMN_2608184 | Il6st | 1.29 | 1.26 |
| ILMN_1763326 | C5orf25 | | 1.48 | ILMN_2599392 | 4732471D19Rik | 1.03 | 1.26 |
| ILMN_1787212 | CDKN1A | | 1.33 | ILMN_2846776 | Cdkn1a | 1.17 | 1.25 |
| ILMN_2053281 | C14orf149 | | 1.42 | ILMN_1230505 | 2810055F11Rik | 1.08 | 1.25 |
| ILMN_1787518 | GSN | | 0.80 | ILMN_2679386 | Gsn | 1.69 | 1.25 |
| ILMN_1697916 | GYLTL1B | | 1.41 | ILMN_1235131 | Gyltl1b | 1.08 | 1.25 |
| ILMN_2396020 | DUSP6 | | 1.43 | ILMN_1248537 | Dusp6 | 1.06 | 1.24 |
| ILMN_1709434 | VIT | | 0.74 | ILMN_2477870 | Vit | 1.72 | 1.23 |
| ILMN_2311796 | TES | | 1.22 | ILMN_1218592 | Tes | 1.21 | 1.21 |
| ILMN_1764043 | TTL | | 1.37 | ILMN_2961165 | Ttl | 1.05 | 1.21 |
| ILMN_2194467 | SGCB | | 0.73 | ILMN_1222716 | Sgcb | 1.68 | 1.21 |
| ILMN_1657554 | TSPYL2 | | 1.64 | ILMN_1216663 | Tspyl2 | 0.75 | 1.19 |
| ILMN_1699071 | C21orf7 | | 1.10 | ILMN_1260025 | ORF63 | 1.27 | 1.19 |
| ILMN_1676980 | MTSS1 | | 1.67 | ILMN_1255439 | Mtss1 | 0.68 | 1.18 |
| ILMN_1801443 | TSKU | | 1.51 | ILMN_2673332 | Tsku | 0.84 | 1.18 |
| ILMN_2078466 | DZIP1L | | 1.18 | ILMN_2761422 | Dzip1l | 1.16 | 1.17 |
| ILMN_1709860 | UNC45A | | 1.62 | ILMN_1253134 | Unc45a | 0.71 | 1.17 |
| ILMN_1692785 | KLHL21 | | 1.56 | ILMN_1229082 | Klhl21 | 0.78 | 1.17 |
| ILMN_1793630 | SLC4A3 | | 1.22 | ILMN_2675029 | Slc4a3 | 1.10 | 1.16 |
| ILMN_1656386 | SEC24D | | 0.82 | ILMN_1243179 | Sec24d | 1.49 | 1.16 |
| ILMN_2154950 | ZNF423 | | 1.44 | ILMN_3131693 | Zfp423 | 0.88 | 1.16 |
| ILMN_1700232 | FBXO30 | | 0.87 | ILMN_2431871 | Fbxo30 | 1.45 | 1.16 |
| ILMN_1810037 | RUSC2 | | 0.93 | ILMN_1214953 | Rusc2 | 1.39 | 1.16 |
| ILMN_1673503 | CACNB4 | | 0.91 | ILMN_3123491 | Cacnb4 | 1.39 | 1.15 |
| ILMN_1805132 | PCDH19 | | 1.23 | ILMN_2696829 | Pcdh19 | 1.06 | 1.15 |
| ILMN_1770800 | PODN | | 1.41 | ILMN_1229370 | Podn | 0.84 | 1.13 |
| ILMN_1748141 | AMOTL1 | | 1.25 | ILMN_1253634 | Amotl1 | 1.00 | 1.13 |
| ILMN_2052686 | LCA5 | | 0.93 | ILMN_3024416 | Lca5 | 1.31 | 1.12 |
| ILMN_2263054 | FEZ1 | | 0.91 | ILMN_1213056 | Fez1 | 1.32 | 1.12 |
| ILMN_1761000 | ACER2 | | 0.85 | ILMN_2629112 | Acer2 | 1.35 | 1.10 |
| ILMN_1695016 | QRICH2 | | 1.19 | ILMN_3136019 | Qrich2 | 1.00 | 1.09 |
| ILMN_1796130 | LOC221710 | | 1.49 | ILMN_1245259 | BC024659 | 0.68 | 1.08 |
| ILMN_1764396 | HDAC4 | | 1.18 | ILMN_1219682 | Hdac4 | 0.98 | 1.08 |
| ILMN_1684440 | PXN | | 1.33 | ILMN_3123303 | Pxn | 0.82 | 1.07 |
| ILMN_1759513 | RND3 | | 0.90 | ILMN_1230157 | Rnd3 | 1.24 | 1.07 |
| ILMN_1659766 | BAG3 | | 1.08 | ILMN_1226195 | Bag3 | 1.04 | 1.06 |
| ILMN_1652749 | ERF | | 1.32 | ILMN_2651005 | Erf | 0.80 | 1.06 |
| ILMN_1698191 | SCML2 | | 1.48 | ILMN_3089660 | Scml2 | 0.62 | 1.05 |
| ILMN_1672596 | BCAR1 | | 1.26 | ILMN_2743133 | Bcar1 | 0.83 | 1.04 |
| ILMN_1701468 | HGFAC | | 1.22 | ILMN_2994299 | Hgfac | 0.86 | 1.04 |
| ILMN_1773059 | GPR124 | | 0.93 | ILMN_1214180 | Gpr124 | 1.13 | 1.03 |
| ILMN_2211780 | SLC25A4 | | 1.28 | ILMN_1225312 | Slc25a4 | 0.78 | 1.03 |
| ILMN_1682495 | FOXP1 | | 0.75 | ILMN_1252078 | Foxp1 | 1.31 | 1.03 |
| ILMN_2407851 | IL17RD | | 0.92 | ILMN_1248657 | Il17rd | 1.14 | 1.03 |
| ILMN_2174127 | DCBLD2 | | 1.02 | ILMN_2430775 | Dcbld2 | 1.03 | 1.02 |
| ILMN_1680273 | MYOCD | | 0.81 | ILMN_2435360 | Myocd | 1.23 | 1.02 |
| ILMN_1652777 | CDC42EP2 | | 0.63 | ILMN_1239294 | Cdc42ep2 | 1.41 | 1.02 |
| ILMN_1807304 | MBNL1 | | 1.10 | ILMN_2766253 | Mbnl1 | 0.89 | 1.00 |
| ILMN_1810486 | RAB34 | | 1.27 | ILMN_3029294 | Rab34 | 0.72 | 0.99 |
| ILMN_1811616 | EEPD1 | | 0.75 | ILMN_2709179 | Eepd1 | 1.23 | 0.99 |
| ILMN_1712505 | KDELC1 | | 0.98 | ILMN_1249999 | Kdelc1 | 1.00 | 0.99 |
| ILMN_1730986 | MALT1 | | 0.83 | ILMN_1216818 | Malt1 | 1.15 | 0.99 |
| ILMN_1732967 | KIAA1949 | | 0.65 | ILMN_1252831 | 2310014H01Rik | 1.31 | 0.98 |
| ILMN_1727194 | CALU | | 1.12 | ILMN_2771956 | Calu | 0.84 | 0.98 |
| ILMN_1765606 | YAF2 | | 1.15 | ILMN_2420095 | Yaf2 | 0.79 | 0.97 |
| ILMN_1710598 | SKI | | 1.07 | ILMN_2488360 | Ski | 0.86 | 0.97 |
| ILMN_2325610 | AKT3 | | 0.93 | ILMN_2904321 | Akt3 | 1.01 | 0.97 |
| ILMN_1804277 | SPRED1 | | 1.02 | ILMN_2490252 | Spred1 | 0.91 | 0.97 |
| ILMN_2084059 | SLC12A4 | | 0.95 | ILMN_2629731 | Slc12a4 | 0.92 | 0.93 |
| ILMN_1667267 | PCBP4 | | 0.74 | ILMN_2607408 | Pcbp4 | 1.10 | 0.92 |
| ILMN_1685580 | CBLB | | 1.03 | ILMN_2702039 | Cblb | 0.80 | 0.91 |
| ILMN_1807181 | BACH1 | | 0.78 | ILMN_2741677 | Bach1 | 1.04 | 0.91 |
| ILMN_1801313 | SIAH2 | | 0.86 | ILMN_1226332 | Siah2 | 0.95 | 0.91 |
| ILMN_1676241 | BCOR | | 1.03 | ILMN_1228091 | Bcor | 0.77 | 0.90 |
| ILMN_2388965 | GEFT | | 0.97 | ILMN_1259949 | D10Ertd610e | 0.83 | 0.90 |
| ILMN_1652609 | RGNEF | | 0.73 | ILMN_1229911 | Rgnef | 1.05 | 0.89 |
| ILMN_1695290 | FERMT2 | | 0.80 | ILMN_2670022 | Fermt2 | 0.97 | 0.89 |
| ILMN_1803995 | TM7SF3 | | 0.85 | ILMN_2491392 | Tm7sf3 | 0.92 | 0.88 |
| ILMN_1699723 | RARB | | 0.82 | ILMN_2886828 | Rarb | 0.92 | 0.87 |
| ILMN_2341690 | C17orf81 | | 0.75 | ILMN_1238130 | Rai12 | 0.98 | 0.86 |
| ILMN_1665455 | DCUN1D3 | | 1.03 | ILMN_2775022 | Dcun1d3 | 0.69 | 0.86 |
| ILMN_1773751 | HRAS | | 0.87 | ILMN_2481071 | Hras1 | 0.83 | 0.85 |
| ILMN_1800412 | BMP1 | | 0.78 | ILMN_2713538 | Bmp1 | 0.90 | 0.84 |
| ILMN_1770811 | PELO | | 0.91 | ILMN_2853658 | Pelo | 0.75 | 0.83 |
| ILMN_1815666 | ATP2A2 | | 0.73 | ILMN_1239742 | Atp2a2 | 0.92 | 0.82 |
| ILMN_1714352 | DMWD | | 0.73 | ILMN_2914010 | Dmwd | 0.90 | 0.81 |
| ILMN_2345292 | FXYD1 | | 0.75 | ILMN_3108783 | Fxyd1 | 0.79 | 0.77 |
| ILMN_1769412 | RAPGEF1 | | 0.69 | ILMN_3140516 | Rapgef1 | 0.85 | 0.77 |
| ILMN_2405305 | ARNTL | | 0.81 | ILMN_2707510 | Arntl | 0.72 | 0.77 |
| ILMN_2383934 | ITGB1 | | 0.84 | ILMN_2609304 | Itgb1 | 0.69 | 0.77 |
| ILMN_1707391 | STXBP4 | | 0.74 | ILMN_2742786 | Stxbp4 | 0.72 | 0.73 |
| ILMN_2345824 | PCDHGC3 | | 0.63 | ILMN_2890002 | Pcdhgc3 | 0.79 | 0.71 |
| ILMN_2331348 | TCOF1 | | 0.76 | ILMN_2608933 | Tcof1 | 0.62 | 0.69 |
| **Down-regulated in the MaSC-enriched subset** | | | | | | | |
| ID human | | symbol | log Fold Change | ID mouse | symbol | log Fold Change | average  log Fold  Change |
| ILMN_1730777 | | KRT19 | -5.34 | ILMN_2614462 | Krt19 | -2.84 | -4.09 |
| ILMN_1771482 | | KIAA1324 | -5.32 | ILMN_1258347 | 5330417C22Rik | -2.70 | -4.01 |
| ILMN_1791123 | | TMPRSS2 | -4.49 | ILMN_1223880 | Tmprss2 | -3.29 | -3.89 |
| ILMN_1766650 | | FOXA1 | -3.94 | ILMN_1237195 | Foxa1 | -3.80 | -3.87 |
| ILMN_1680757 | | LRRC26 | -4.18 | ILMN_2682268 | Lrrc26 | -3.23 | -3.71 |
| ILMN_1795342 | | MLPH | -4.84 | ILMN_3162347 | Mlph | -2.41 | -3.62 |
| ILMN_1746801 | | CGN | -3.96 | ILMN_2649148 | Cgn | -3.26 | -3.61 |
| ILMN_1808789 | | MYO5C | -3.93 | ILMN_2683244 | Myo5c | -3.16 | -3.54 |
| ILMN_2410612 | | DMBT1 | -3.76 | ILMN_2629239 | Dmbt1 | -3.26 | -3.51 |
| ILMN_2304495 | | PPP1R1B | -3.26 | ILMN_2954824 | Ppp1r1b | -3.44 | -3.35 |
| ILMN_1668822 | | BATF | -4.59 | ILMN_2761900 | Batf | -1.92 | -3.25 |
| ILMN_1756992 | | MUC1 | -3.94 | ILMN_1250138 | Muc1 | -2.57 | -3.25 |
| ILMN_2269256 | | DNAJC12 | -2.91 | ILMN_1229993 | Dnajc12 | -3.59 | -3.25 |
| ILMN_1723042 | | CLDN3 | -3.66 | ILMN_2634167 | Cldn3 | -2.82 | -3.24 |
| ILMN_1730977 | | SLC44A4 | -3.37 | ILMN_2970023 | Slc44a4 | -3.08 | -3.22 |
| ILMN_1708580 | | PDZK1IP1 | -3.99 | ILMN_2618935 | Pdzk1ip1 | -2.29 | -3.14 |
| ILMN_1677920 | | LTF | -4.32 | ILMN_2754364 | Ltf | -1.96 | -3.14 |
| ILMN_1770505 | | BIK | -3.51 | ILMN_2717011 | Bik | -2.72 | -3.12 |
| ILMN_1671154 | | TMPRSS13 | -3.16 | ILMN_1226348 | Tmprss13 | -2.97 | -3.07 |
| ILMN_1659610 | | TJP3 | -3.09 | ILMN_2723524 | Tjp3 | -3.04 | -3.07 |
| ILMN_1656310 | | IDO1 | -2.35 | ILMN_1223600 | Ido1 | -3.77 | -3.06 |
| ILMN_1686573 | | DEFB1 | -4.48 | ILMN_2804685 | Defb1 | -1.48 | -2.98 |
| ILMN_1803219 | | TMC4 | -3.65 | ILMN_2435822 | Tmc4 | -2.30 | -2.98 |
| ILMN_1669781 | | PRLR | -2.77 | ILMN_2617005 | Prlr | -3.18 | -2.97 |
| ILMN_1796337 | | CXCL17 | -2.46 | ILMN_1226394 | Cxcl17 | -3.47 | -2.97 |
| ILMN_1689046 | | RBM47 | -3.57 | ILMN_2720836 | Rbm47 | -2.32 | -2.95 |
| ILMN_1751346 | | ERBB3 | -4.68 | ILMN_1250597 | Erbb3 | -1.20 | -2.94 |
| ILMN_1689146 | | GABRP | -3.65 | ILMN_1251237 | Gabrp | -2.22 | -2.93 |
| ILMN_1803408 | | KRT18 | -3.30 | ILMN_2711267 | Krt18 | -2.46 | -2.88 |
| ILMN_1697460 | | REEP6 | -3.03 | ILMN_2661650 | Reep6 | -2.56 | -2.80 |
| ILMN_1727288 | | EVPL | -2.74 | ILMN_2435835 | Evpl | -2.83 | -2.79 |
| ILMN_1769388 | | GJB2 | -3.12 | ILMN_2999627 | Gjb2 | -2.45 | -2.79 |
| ILMN_1755649 | | SLC16A5 | -2.49 | ILMN_2461172 | Slc16a5 | -3.04 | -2.76 |
| ILMN_1695924 | | KLK11 | -3.24 | ILMN_2780280 | Klk11 | -2.25 | -2.75 |
| ILMN_1736527 | | TFCP2L1 | -3.02 | ILMN_2769832 | Tcfcp2l1 | -2.46 | -2.74 |
| ILMN_1764082 | | MBOAT1 | -1.97 | ILMN_1225029 | Mboat1 | -3.49 | -2.73 |
| ILMN_1753101 | | VTCN1 | -4.23 | ILMN_2673621 | Vtcn1 | -1.23 | -2.73 |
| ILMN_2341548 | | MYO5B | -2.82 | ILMN_2539489 | Myo5b | -2.57 | -2.69 |
| ILMN_1678535 | | ESR1 | -2.96 | ILMN_2726412 | Esr1 | -2.40 | -2.68 |
| ILMN_2059689 | | TMEM54 | -2.41 | ILMN_2919393 | Tmem54 | -2.94 | -2.67 |
| ILMN_1750674 | | SDSL | -3.35 | ILMN_1231627 | Sdsl | -2.00 | -2.67 |
| ILMN_2382505 | | SLC22A18 | -2.12 | ILMN_3152079 | Slc22a18 | -3.22 | -2.67 |
| ILMN_2143685 | | CLDN7 | -2.86 | ILMN_2595477 | Cldn7 | -2.48 | -2.67 |
| ILMN_1741014 | | SLC28A3 | -2.76 | ILMN_2634129 | Slc28a3 | -2.47 | -2.62 |
| ILMN_2072568 | | CLDN8 | -3.70 | ILMN_2623145 | Cldn8 | -1.47 | -2.59 |
| ILMN_1699989 | | BNIPL | -2.93 | ILMN_1213321 | Bnipl | -2.23 | -2.58 |
| ILMN_1746517 | | KYNU | -3.17 | ILMN_1214750 | Kynu | -1.98 | -2.57 |
| ILMN_2407824 | | ATP1B1 | -2.16 | ILMN_2767615 | Atp1b1 | -2.98 | -2.57 |
| ILMN_1727689 | | TNFAIP2 | -3.24 | ILMN_2841289 | Tnfaip2 | -1.89 | -2.57 |
| ILMN_1658639 | | SLC46A3 | -1.91 | ILMN_2627733 | Slc46a3 | -3.21 | -2.56 |
| ILMN_1729433 | | LIPH | -1.61 | ILMN_1217063 | Liph | -3.49 | -2.55 |
| ILMN_1810915 | | FAAH | -1.64 | ILMN_2657980 | Faah | -3.41 | -2.53 |
| ILMN_1685709 | | TMEM125 | -2.22 | ILMN_2703138 | Tmem125 | -2.79 | -2.51 |
| ILMN_1763666 | | ALDH3B2 | -3.66 | ILMN_2746937 | Aldh3b2 | -1.33 | -2.49 |
| ILMN_1749327 | | MAPK13 | -3.24 | ILMN_2955452 | Mapk13 | -1.72 | -2.48 |
| ILMN_1724686 | | CLDN1 | -3.16 | ILMN_2870295 | Cldn1 | -1.80 | -2.48 |
| ILMN_1753143 | | RHPN2 | -2.64 | ILMN_2608133 | Rhpn2 | -2.26 | -2.45 |
| ILMN_1716407 | | SORBS2 | -3.08 | ILMN_1235808 | Sorbs2 | -1.82 | -2.45 |
| ILMN_2413158 | | PODXL | -2.67 | ILMN_2604494 | Podxl | -2.16 | -2.42 |
| ILMN_1681526 | | SLC5A1 | -3.05 | ILMN_2715270 | Slc5a1 | -1.79 | -2.42 |
| ILMN_1719599 | | SYTL4 | -3.24 | ILMN_2751935 | Sytl4 | -1.58 | -2.41 |
| ILMN_1727589 | | SULT2B1 | -2.17 | ILMN_2686353 | Sult2b1 | -2.65 | -2.41 |
| ILMN_1698484 | | RHOV | -2.04 | ILMN_2859528 | Rhov | -2.77 | -2.40 |
| ILMN_1774265 | | C2orf82 | -2.94 | ILMN_2598181 | 3110079O15Rik | -1.86 | -2.40 |
| ILMN_2161330 | | SPDEF | -2.46 | ILMN_1258162 | Spdef | -2.34 | -2.40 |
| ILMN_1814787 | | ICA1 | -3.21 | ILMN_1245768 | Ica1 | -1.57 | -2.39 |
| ILMN_1745623 | | EFCAB4A | -3.03 | ILMN_2913222 | Efcab4a | -1.71 | -2.37 |
| ILMN_1769934 | | GRHL1 | -2.18 | ILMN_1246419 | Grhl1 | -2.45 | -2.32 |
| ILMN_2081070 | | BTC | -1.13 | ILMN_2734097 | Btc | -3.49 | -2.31 |
| ILMN_1782429 | | TMEM56 | -1.02 | ILMN_2966162 | Tmem56 | -3.60 | -2.31 |
| ILMN_2132458 | | CLDN4 | -1.75 | ILMN_1223949 | Cldn4 | -2.87 | -2.31 |
| ILMN_1667199 | | SQRDL | -2.90 | ILMN_1247947 | Sqrdl | -1.70 | -2.30 |
| ILMN_1769201 | | ELF3 | -3.08 | ILMN_2850233 | Elf3 | -1.50 | -2.29 |
| ILMN_1705750 | | TGM2 | -3.70 | ILMN_2692615 | Tgm2 | -0.81 | -2.25 |
| ILMN_1784467 | | NUP210 | -1.83 | ILMN_1257579 | Nup210 | -2.66 | -2.25 |
| ILMN_1716815 | | CEACAM1 | -1.79 | ILMN_3126609 | Ceacam1 | -2.65 | -2.22 |
| ILMN_1654013 | | C17orf28 | -2.13 | ILMN_3132223 | C630004H02Rik | -2.24 | -2.19 |
| ILMN_1701170 | | BARX2 | -2.62 | ILMN_2437728 | Barx2 | -1.73 | -2.18 |
| ILMN_1795118 | | SIDT1 | -1.58 | ILMN_1248211 | Sidt1 | -2.69 | -2.14 |
| ILMN_1802642 | | TOM1L1 | -2.18 | ILMN_1249959 | Tom1l1 | -2.09 | -2.13 |
| ILMN_1739605 | | LYPD3 | -2.93 | ILMN_1248916 | Lypd3 | -1.33 | -2.13 |
| ILMN_1671486 | | HOMER2 | -2.14 | ILMN_2588474 | Homer2 | -2.11 | -2.13 |
| ILMN_1811729 | | CBLC | -2.28 | ILMN_2738699 | Cblc | -1.96 | -2.12 |
| ILMN_2050790 | | C11orf52 | -3.15 | ILMN_2707595 | 2310030G06Rik | -1.08 | -2.11 |
| ILMN_1699887 | | ST14 | -2.48 | ILMN_2624209 | St14 | -1.73 | -2.11 |
| ILMN_1749403 | | TSPAN33 | -1.78 | ILMN_1219904 | Tspan33 | -2.41 | -2.09 |
| ILMN_1741054 | | SLC5A6 | -1.94 | ILMN_1225056 | Slc5a6 | -2.22 | -2.08 |
| ILMN_1706612 | | WFDC2 | -2.15 | ILMN_1236758 | Wfdc2 | -1.98 | -2.07 |
| ILMN_1813100 | | KIAA1244 | -1.28 | ILMN_2781181 | D10Bwg1379e | -2.82 | -2.05 |
| ILMN_2405254 | | GRB7 | -1.74 | ILMN_2773169 | Grb7 | -2.35 | -2.05 |
| ILMN_1728106 | | TNF | -2.01 | ILMN_2899863 | Tnf | -2.06 | -2.04 |
| ILMN_1742705 | | SLC39A11 | -2.42 | ILMN_2668706 | Slc39a11 | -1.64 | -2.03 |
| ILMN_1670064 | | C1orf210 | -1.17 | ILMN_1221084 | 2610528J11Rik | -2.88 | -2.03 |
| ILMN_1791826 | | RAB25 | -1.79 | ILMN_2776087 | Rab25 | -2.26 | -2.02 |
| ILMN_1800602 | | GCA | -1.26 | ILMN_2688888 | Gca | -2.78 | -2.02 |
| ILMN_1769433 | | IQGAP2 | -2.06 | ILMN_1218051 | Iqgap2 | -1.97 | -2.01 |
| ILMN_2060413 | | CD24 | -2.93 | ILMN_1237868 | Cd24a | -1.07 | -2.00 |
| ILMN_1813746 | | CORO2A | -1.61 | ILMN_2768936 | Coro2a | -2.33 | -1.97 |
| ILMN_1785095 | | ATP6V0E2 | -2.03 | ILMN_2832658 | Atp6v0e2 | -1.87 | -1.95 |
| ILMN_1799280 | | BDH1 | -1.32 | ILMN_1231553 | Bdh1 | -2.58 | -1.95 |
| ILMN_1692219 | | RAB11FIP1 | -3.17 | ILMN_1221771 | Rab11fip1 | -0.73 | -1.95 |
| ILMN_1664691 | | DAPP1 | -1.75 | ILMN_1253354 | Dapp1 | -2.13 | -1.94 |
| ILMN_1766405 | | GOLM1 | -3.01 | ILMN_2623536 | Golm1 | -0.86 | -1.94 |
| ILMN_1769259 | | ANO9 | -1.03 | ILMN_1242413 | Ano9 | -2.81 | -1.92 |
| ILMN_2352131 | | ERBB2 | -2.33 | ILMN_2739424 | Erbb2 | -1.51 | -1.92 |
| ILMN_1710644 | | MARVELD3 | -0.99 | ILMN_2632585 | Marveld3 | -2.80 | -1.89 |
| ILMN_1788416 | | FAM108C1 | -1.32 | ILMN_1217822 | Fam108c | -2.45 | -1.89 |
| ILMN_1813846 | | P2RX4 | -2.73 | ILMN_1237644 | P2rx4 | -0.99 | -1.86 |
| ILMN_1669114 | | WNK4 | -1.64 | ILMN_2518744 | Wnk4 | -2.05 | -1.85 |
| ILMN_1684205 | | CIB1 | -2.53 | ILMN_2621847 | Cib1 | -1.16 | -1.85 |
| ILMN_1722981 | | TLR5 | -2.60 | ILMN_2440602 | Tlr5 | -1.09 | -1.84 |
| ILMN_1687495 | | SLC37A1 | -1.72 | ILMN_1214066 | Slc37a1 | -1.95 | -1.84 |
| ILMN_1809613 | | NGEF | -1.11 | ILMN_1245037 | Ngef | -2.56 | -1.83 |
| ILMN_1652490 | | MANSC1 | -1.49 | ILMN_2848273 | Mansc1 | -2.17 | -1.83 |
| ILMN_1758523 | | ABCA3 | -2.02 | ILMN_2514655 | Abca3 | -1.64 | -1.83 |
| ILMN_1749044 | | PVRL4 | -2.58 | ILMN_1235878 | Pvrl4 | -1.06 | -1.82 |
| ILMN_1679614 | | SGSM3 | -1.49 | ILMN_2987294 | Sgsm3 | -2.12 | -1.81 |
| ILMN_1713846 | | PPM1H | -2.52 | ILMN_2484932 | Ppm1h | -1.08 | -1.80 |
| ILMN_1750497 | | NIACR1 | -2.60 | ILMN_2847115 | Niacr1 | -1.01 | -1.80 |
| ILMN_1805992 | | KIAA1598 | -2.43 | ILMN_2944657 | 4930506M07Rik | -1.17 | -1.80 |
| ILMN_2392352 | | CTPS2 | -2.38 | ILMN_2632076 | Ctps2 | -1.21 | -1.80 |
| ILMN_2170949 | | SNX10 | -2.69 | ILMN_2609614 | Snx10 | -0.90 | -1.79 |
| ILMN_1664303 | | HTATIP2 | -2.27 | ILMN_2603834 | Htatip2 | -1.31 | -1.79 |
| ILMN_1674135 | | RALGPS1 | -1.39 | ILMN_1230211 | Ralgps1 | -2.17 | -1.78 |
| ILMN_1676891 | | CDC2L6 | -1.43 | ILMN_2644664 | Cdc2l6 | -2.12 | -1.78 |
| ILMN_1662795 | | CA2 | -1.16 | ILMN_2606746 | Car2 | -2.37 | -1.77 |
| ILMN_1803197 | | RAB3IP | -1.54 | ILMN_2626114 | Rab3ip | -1.99 | -1.76 |
| ILMN_2139970 | | ALDH1A3 | -1.35 | ILMN_1237578 | Aldh1a3 | -2.16 | -1.75 |
| ILMN_2396444 | | CD14 | -1.69 | ILMN_2742075 | Cd14 | -1.81 | -1.75 |
| ILMN_1781374 | | TUFT1 | -1.49 | ILMN_2419185 | Tuft1 | -2.00 | -1.75 |
| ILMN_2142353 | | GRTP1 | -1.94 | ILMN_2737940 | Grtp1 | -1.53 | -1.74 |
| ILMN_1711888 | | COBL | -1.78 | ILMN_2951682 | Cobl | -1.68 | -1.73 |
| ILMN_1707088 | | DENND2D | -1.93 | ILMN_2657682 | Dennd2d | -1.53 | -1.73 |
| ILMN_1674498 | | C7orf46 | -1.60 | ILMN_1217670 | D330028D13Rik | -1.82 | -1.71 |
| ILMN_1804461 | | MYH14 | -1.95 | ILMN_1217519 | Myh14 | -1.46 | -1.70 |
| ILMN_1749834 | | LOC388588 | -1.75 | ILMN_2893564 | 1190007F08Rik | -1.64 | -1.69 |
| ILMN_1691572 | | TST | -1.59 | ILMN_2493175 | Tst | -1.78 | -1.69 |
| ILMN_1778650 | | VILL | -0.61 | ILMN_2846432 | Vill | -2.75 | -1.68 |
| ILMN_1683470 | | TMEM139 | -1.26 | ILMN_2600822 | Tmem139 | -2.10 | -1.68 |
| ILMN_1700583 | | ZNF750 | -0.69 | ILMN_2654571 | Zfp750 | -2.65 | -1.67 |
| ILMN_2345142 | | SULF2 | -1.24 | ILMN_1239293 | Sulf2 | -2.10 | -1.67 |
| ILMN_1735495 | | TBC1D8 | -2.58 | ILMN_1255779 | Tbc1d8 | -0.76 | -1.67 |
| ILMN_1792455 | | TMEM158 | -1.86 | ILMN_3160486 | Tmem158 | -1.48 | -1.67 |
| ILMN_1811515 | | DLG3 | -2.52 | ILMN_2993836 | Dlg3 | -0.81 | -1.67 |
| ILMN_2256953 | | CASZ1 | -0.76 | ILMN_2665095 | Casz1 | -2.57 | -1.67 |
| ILMN_2227533 | | ABHD14B | -1.74 | ILMN_3007862 | Abhd14b | -1.59 | -1.67 |
| ILMN_1705107 | | SDCBP2 | -2.15 | ILMN_2825446 | Sdcbp2 | -1.17 | -1.66 |
| ILMN_1725510 | | DHCR24 | -2.32 | ILMN_2747031 | Dhcr24 | -1.00 | -1.66 |
| ILMN_1725471 | | GK | -2.24 | ILMN_2776721 | Gyk | -1.06 | -1.65 |
| ILMN_1792168 | | GALE | -1.39 | ILMN_1237990 | Gale | -1.90 | -1.65 |
| ILMN_1706571 | | SLC35D2 | -1.78 | ILMN_1234014 | Slc35d2 | -1.51 | -1.64 |
| ILMN_2159453 | | STXBP2 | -2.34 | ILMN_2657728 | Stxbp2 | -0.94 | -1.64 |
| ILMN_1806030 | | PPL | -1.58 | ILMN_3155363 | Ppl | -1.68 | -1.63 |
| ILMN_1781761 | | ENPP4 | -2.06 | ILMN_2762380 | Enpp4 | -1.19 | -1.63 |
| ILMN_2142117 | | LYPLAL1 | -2.03 | ILMN_1248108 | Lyplal1 | -1.22 | -1.63 |
| ILMN_2360307 | | SUSD4 | -1.53 | ILMN_2677824 | Susd4 | -1.72 | -1.62 |
| ILMN_1659913 | | ISG20 | -1.99 | ILMN_2735615 | Isg20 | -1.25 | -1.62 |
| ILMN_1671777 | | FGF13 | -1.54 | ILMN_2745480 | Fgf13 | -1.70 | -1.62 |
| ILMN_1805636 | | PGAP3 | -1.87 | ILMN_2711000 | Pgap3 | -1.36 | -1.62 |
| ILMN_1661492 | | ARRDC1 | -1.42 | ILMN_1255050 | Arrdc1 | -1.81 | -1.61 |
| ILMN_1736965 | | SIGIRR | -1.43 | ILMN_2673121 | Sigirr | -1.79 | -1.61 |
| ILMN_1713688 | | DHX32 | -1.65 | ILMN_2643150 | Dhx32 | -1.55 | -1.60 |
| ILMN_1684497 | | PYROXD2 | -1.68 | ILMN_1249560 | Pyroxd2 | -1.51 | -1.60 |
| ILMN_1724148 | | ORAI1 | -2.08 | ILMN_1236368 | Orai1 | -1.08 | -1.58 |
| ILMN_2375992 | | SPINT1 | -1.27 | ILMN_1237186 | Spint1 | -1.86 | -1.57 |
| ILMN_1757872 | | PRAGMIN | -1.01 | ILMN_2731237 | D8Ertd82e | -2.09 | -1.55 |
| ILMN_2173294 | | THNSL2 | -1.88 | ILMN_2773485 | Thnsl2 | -1.22 | -1.55 |
| ILMN_1672743 | | ZNF334 | -1.69 | ILMN_2855298 | Zfp334 | -1.40 | -1.55 |
| ILMN_1733110 | | RASSF7 | -1.79 | ILMN_2778094 | Rassf7 | -1.29 | -1.54 |
| ILMN_1792726 | | TDRKH | -1.80 | ILMN_2703698 | Tdrkh | -1.23 | -1.52 |
| ILMN_1755234 | | SSH3 | -1.96 | ILMN_2650725 | Ssh3 | -1.07 | -1.51 |
| ILMN_1727605 | | FRK | -2.30 | ILMN_2615513 | Frk | -0.72 | -1.51 |
| ILMN_1680925 | | SLC9A3R1 | -1.53 | ILMN_1240256 | Slc9a3r1 | -1.49 | -1.51 |
| ILMN_1738675 | | PTPN6 | -1.65 | ILMN_1220996 | Ptpn6 | -1.36 | -1.51 |
| ILMN_1723768 | | NLRX1 | -2.02 | ILMN_2958484 | Nlrx1 | -0.98 | -1.50 |
| ILMN_1669928 | | ARHGEF16 | -1.61 | ILMN_1239332 | Arhgef16 | -1.38 | -1.49 |
| ILMN_1711994 | | TCIRG1 | -1.90 | ILMN_2643876 | Tcirg1 | -1.08 | -1.49 |
| ILMN_2199389 | | VIPR1 | -1.45 | ILMN_1231649 | Vipr1 | -1.53 | -1.49 |
| ILMN_1810875 | | SYNGR1 | -1.45 | ILMN_3101732 | Syngr1 | -1.51 | -1.48 |
| ILMN_1754179 | | AP1G2 | -1.61 | ILMN_1246903 | Ap1g2 | -1.33 | -1.47 |
| ILMN_1703370 | | ZDHHC12 | -2.08 | ILMN_2520032 | Zdhhc12 | -0.83 | -1.46 |
| ILMN_1723020 | | MAP3K1 | -1.93 | ILMN_3006611 | Map3k1 | -0.98 | -1.45 |
| ILMN_1701455 | | FBXO6 | -1.36 | ILMN_2660175 | Fbxo6 | -1.54 | -1.45 |
| ILMN_1684690 | | HDAC11 | -1.00 | ILMN_2614912 | Hdac11 | -1.90 | -1.45 |
| ILMN_1660544 | | ARRDC4 | -1.84 | ILMN_2648967 | Arrdc4 | -1.05 | -1.45 |
| ILMN_1687235 | | HPN | -1.34 | ILMN_1221781 | Hpn | -1.55 | -1.45 |
| ILMN_2043615 | | C17orf90 | -1.59 | ILMN_2680665 | 1810049H13Rik | -1.27 | -1.43 |
| ILMN_1700159 | | NIPSNAP3A | -1.94 | ILMN_2657376 | Nipsnap3a | -0.93 | -1.43 |
| ILMN_1659688 | | LGALS3BP | -1.70 | ILMN_1258526 | Lgals3bp | -1.14 | -1.42 |
| ILMN_1784380 | | DTX3L | -1.61 | ILMN_1220813 | Dtx3l | -1.23 | -1.42 |
| ILMN_1710170 | | PPAP2C | -1.46 | ILMN_1249666 | Ppap2c | -1.37 | -1.41 |
| ILMN_1695092 | | WRB | -1.27 | ILMN_2473122 | Wrb | -1.55 | -1.41 |
| ILMN_1653496 | | GLUL | -1.30 | ILMN_2644496 | Glul | -1.52 | -1.41 |
| ILMN_1753584 | | KRT8 | -1.64 | ILMN_1221157 | Krt8 | -1.15 | -1.39 |
| ILMN_1777881 | | TSPAN17 | -1.29 | ILMN_2817714 | Tspan17 | -1.49 | -1.39 |
| ILMN_2160210 | | EPCAM | -1.24 | ILMN_1240539 | Epcam | -1.52 | -1.38 |
| ILMN_1780671 | | PLEKHG3 | -1.75 | ILMN_1221920 | Plekhg3 | -1.01 | -1.38 |
| ILMN_1660837 | | CLCN3 | -1.13 | ILMN_2603918 | Clcn3 | -1.63 | -1.38 |
| ILMN_1801914 | | SH3BP2 | -1.56 | ILMN_1255766 | Sh3bp2 | -1.19 | -1.37 |
| ILMN_1811551 | | DERA | -2.01 | ILMN_1227366 | Dera | -0.74 | -1.37 |
| ILMN_1771264 | | ELL3 | -1.46 | ILMN_2627179 | Ell3 | -1.28 | -1.37 |
| ILMN_2043079 | | ILDR1 | -1.69 | ILMN_2734693 | Ildr1 | -1.05 | -1.37 |
| ILMN_1769264 | | MCCC2 | -1.88 | ILMN_1259582 | Mccc2 | -0.86 | -1.37 |
| ILMN_1803277 | | MVP | -1.89 | ILMN_2887065 | Mvp | -0.84 | -1.36 |
| ILMN_1815656 | | SERINC3 | -1.16 | ILMN_2787844 | Serinc3 | -1.56 | -1.36 |
| ILMN_1742824 | | SPATA13 | -0.89 | ILMN_2751037 | Spata13 | -1.84 | -1.36 |
| ILMN_1815626 | | DHCR7 | -1.73 | ILMN_2660471 | Dhcr7 | -0.98 | -1.36 |
| ILMN_1775703 | | TRAPPC6A | -1.52 | ILMN_2643057 | Trappc6a | -1.19 | -1.35 |
| ILMN_1796835 | | RWDD3 | -1.23 | ILMN_2922728 | Rwdd3 | -1.47 | -1.35 |
| ILMN_1764321 | | ACOT4 | -1.74 | ILMN_2622613 | Acot4 | -0.96 | -1.35 |
| ILMN_1756877 | | C14orf179 | -1.98 | ILMN_1236344 | 1700019E19Rik | -0.71 | -1.35 |
| ILMN_1730229 | | CGNL1 | -1.64 | ILMN_2736038 | Cgnl1 | -1.05 | -1.34 |
| ILMN_1772731 | | HAGH | -1.67 | ILMN_2734661 | Hagh | -1.00 | -1.33 |
| ILMN_2323944 | | FAM110A | -1.61 | ILMN_2740890 | Fam110a | -1.04 | -1.33 |
| ILMN_1700384 | | KIAA1522 | -1.43 | ILMN_2696592 | C77080 | -1.22 | -1.32 |
| ILMN_1743427 | | SCYL3 | -1.24 | ILMN_2588521 | Scyl3 | -1.39 | -1.32 |
| ILMN_1726466 | | HDHD3 | -1.26 | ILMN_2608145 | Hdhd3 | -1.38 | -1.32 |
| ILMN_1796177 | | GIPC1 | -1.66 | ILMN_1225966 | Gipc1 | -0.95 | -1.30 |
| ILMN_1717052 | | STARD10 | -1.31 | ILMN_2759762 | Stard10 | -1.27 | -1.29 |
| ILMN_1752988 | | C11orf17 | -1.62 | ILMN_2729762 | D930014E17Rik | -0.96 | -1.29 |
| ILMN_1809208 | | KIAA1543 | -1.49 | ILMN_1235207 | 2310057J16Rik | -1.08 | -1.28 |
| ILMN_1711030 | | OPLAH | -1.32 | ILMN_2662191 | Oplah | -1.25 | -1.28 |
| ILMN_1724612 | | SLC25A35 | -0.95 | ILMN_1258728 | Slc25a35 | -1.61 | -1.28 |
| ILMN_1687303 | | ACAD10 | -1.74 | ILMN_2821158 | Acad10 | -0.81 | -1.28 |
| ILMN_1697267 | | PRKCZ | -0.72 | ILMN_3158919 | Prkcz | -1.82 | -1.27 |
| ILMN_1657631 | | STAP2 | -1.19 | ILMN_2758562 | Stap2 | -1.34 | -1.26 |
| ILMN_2153373 | | LRBA | -1.43 | ILMN_2659960 | Lrba | -1.10 | -1.26 |
| ILMN_1725241 | | GSTK1 | -1.77 | ILMN_2792924 | Gstk1 | -0.76 | -1.26 |
| ILMN_1724207 | | IVD | -1.44 | ILMN_2728379 | Ivd | -1.07 | -1.26 |
| ILMN_1771126 | | RORC | -1.48 | ILMN_2760272 | Rorc | -1.03 | -1.26 |
| ILMN_1737805 | | TPCN1 | -1.76 | ILMN_1217102 | Tpcn1 | -0.75 | -1.25 |
| ILMN_1653429 | | SLC35A3 | -1.51 | ILMN_2974069 | Slc35a3 | -1.00 | -1.25 |
| ILMN_1718946 | | ADAM10 | -1.49 | ILMN_1241149 | Adam10 | -1.02 | -1.25 |
| ILMN_1809437 | | RHBDD2 | -1.72 | ILMN_2709690 | Rhbdd2 | -0.78 | -1.25 |
| ILMN_1681310 | | MFSD7 | -1.52 | ILMN_2766105 | Mfsd7a | -0.98 | -1.25 |
| ILMN_1778347 | | NUDT2 | -1.27 | ILMN_1259148 | Nudt2 | -1.21 | -1.24 |
| ILMN_1719316 | | TMED3 | -0.80 | ILMN_2650532 | Tmed3 | -1.68 | -1.24 |
| ILMN_1678922 | | HERC4 | -1.64 | ILMN_2722469 | Herc4 | -0.84 | -1.24 |
| ILMN_1737475 | | ABHD11 | -1.68 | ILMN_2453209 | Abhd11 | -0.80 | -1.24 |
| ILMN_1802628 | | PPAPDC2 | -1.08 | ILMN_2427467 | Ppapdc2 | -1.39 | -1.24 |
| ILMN_2060115 | | SORL1 | -1.52 | ILMN_1249578 | Sorl1 | -0.95 | -1.23 |
| ILMN_1678579 | | CPT2 | -1.52 | ILMN_2775122 | Cpt2 | -0.94 | -1.23 |
| ILMN_1655821 | | CAPG | -1.60 | ILMN_2773113 | Capg | -0.84 | -1.22 |
| ILMN_2381064 | | TPD52 | -1.38 | ILMN_3091288 | Tpd52 | -1.06 | -1.22 |
| ILMN_2372398 | | ALDH5A1 | -1.59 | ILMN_2664660 | Aldh5a1 | -0.85 | -1.22 |
| ILMN_1767006 | | PSMB8 | -1.24 | ILMN_1226683 | Psmb8 | -1.19 | -1.22 |
| ILMN_1753547 | | STAT5A | -1.39 | ILMN_2976191 | Stat5a | -1.03 | -1.21 |
| ILMN_1768751 | | MTA3 | -1.54 | ILMN_1259783 | Mta3 | -0.88 | -1.21 |
| ILMN_1683883 | | ACY1 | -1.71 | ILMN_2945472 | Acy1 | -0.70 | -1.20 |
| ILMN_1723092 | | CRB3 | -1.46 | ILMN_2755888 | Crb3 | -0.94 | -1.20 |
| ILMN_1799015 | | PXMP2 | -1.60 | ILMN_1219748 | Pxmp2 | -0.80 | -1.20 |
| ILMN_1653480 | | CCDC125 | -1.35 | ILMN_2693679 | Ccdc125 | -1.02 | -1.19 |
| ILMN_1797530 | | CHCHD5 | -1.29 | ILMN_2801540 | Chchd5 | -1.07 | -1.18 |
| ILMN_1703593 | | BAIAP2L1 | -1.50 | ILMN_2665609 | Baiap2l1 | -0.86 | -1.18 |
| ILMN_1683059 | | SIRT5 | -0.99 | ILMN_2711045 | Sirt5 | -1.37 | -1.18 |
| ILMN_1678729 | | SIL1 | -1.48 | ILMN_2720930 | Sil1 | -0.87 | -1.18 |
| ILMN_1749396 | | LSR | -0.86 | ILMN_2699637 | Lsr | -1.49 | -1.17 |
| ILMN_1651364 | | PCBD2 | -1.64 | ILMN_2699126 | Pcbd2 | -0.70 | -1.17 |
| ILMN_1776102 | | PSMD10 | -1.53 | ILMN_3003308 | Psmd10 | -0.80 | -1.16 |
| ILMN_1666109 | | MB | -1.50 | ILMN_2954987 | Mb | -0.82 | -1.16 |
| ILMN_1765520 | | MTIF2 | -1.41 | ILMN_1236180 | Mtif2 | -0.92 | -1.16 |
| ILMN_1729112 | | CHPT1 | -1.37 | ILMN_2740152 | Chpt1 | -0.95 | -1.16 |
| ILMN_1732071 | | HIST2H2BE | -1.07 | ILMN_1231066 | Hist2h2be | -1.25 | -1.16 |
| ILMN_1669497 | | OSBPL10 | -0.83 | ILMN_2754067 | Osbpl10 | -1.49 | -1.16 |
| ILMN_1706687 | | KLHL5 | -1.58 | ILMN_2692484 | Klhl5 | -0.72 | -1.15 |
| ILMN_2193591 | | UNC93B1 | -1.28 | ILMN_2795078 | Unc93b1 | -1.01 | -1.15 |
| ILMN_1748093 | | PAFAH1B3 | -1.42 | ILMN_2640971 | Pafah1b3 | -0.86 | -1.14 |
| ILMN_1711171 | | CREB3L4 | -1.27 | ILMN_1213549 | Creb3l4 | -1.01 | -1.14 |
| ILMN_1814526 | | ADD3 | -0.83 | ILMN_2588671 | Add3 | -1.44 | -1.14 |
| ILMN_1798108 | | C6orf211 | -1.19 | ILMN_2985579 | 1700052N19Rik | -1.08 | -1.14 |
| ILMN_1797384 | | UROS | -1.38 | ILMN_2498173 | Uros | -0.88 | -1.13 |
| ILMN_2367707 | | PKN1 | -1.19 | ILMN_2990872 | Pkn1 | -1.07 | -1.13 |
| ILMN_2415267 | | RREB1 | -1.36 | ILMN_1255511 | Rreb1 | -0.90 | -1.13 |
| ILMN_1701507 | | EHHADH | -1.31 | ILMN_2706120 | Ehhadh | -0.94 | -1.12 |
| ILMN_1705861 | | AP1M2 | -1.08 | ILMN_2925872 | Ap1m2 | -1.17 | -1.12 |
| ILMN_2363058 | | PAOX | -1.40 | ILMN_2795698 | Paox | -0.85 | -1.12 |
| ILMN_1694174 | | TRIM68 | -0.69 | ILMN_2635642 | Trim68 | -1.56 | -1.12 |
| ILMN_1767365 | | PAK1 | -1.12 | ILMN_1214850 | Pak1 | -1.12 | -1.12 |
| ILMN_1684289 | | PNPO | -1.03 | ILMN_2855792 | Pnpo | -1.21 | -1.12 |
| ILMN_1741440 | | SLC35A1 | -1.29 | ILMN_2702767 | Slc35a1 | -0.94 | -1.12 |
| ILMN_1688071 | | NAT1 | -1.54 | ILMN_2994380 | Nat2 | -0.69 | -1.12 |
| ILMN_1763080 | | QARS | -1.55 | ILMN_2989257 | Qars | -0.67 | -1.11 |
| ILMN_1755405 | | PGAP2 | -1.60 | ILMN_2665054 | Pgap2 | -0.62 | -1.11 |
| ILMN_1789405 | | C22orf25 | -1.35 | ILMN_2842999 | D16H22S680E | -0.86 | -1.11 |
| ILMN_1745807 | | TMEM62 | -1.02 | ILMN_2633670 | Tmem62 | -1.18 | -1.10 |
| ILMN_1777853 | | MBOAT2 | -1.29 | ILMN_1214848 | Mboat2 | -0.90 | -1.09 |
| ILMN_2125374 | | CMAS | -1.05 | ILMN_2683128 | Cmas | -1.14 | -1.09 |
| ILMN_1740234 | | GSTO2 | -1.33 | ILMN_2938904 | Gsto2 | -0.85 | -1.09 |
| ILMN_1804051 | | SNX8 | -1.28 | ILMN_2671671 | Snx8 | -0.90 | -1.09 |
| ILMN_1675656 | | PPFIBP2 | -0.85 | ILMN_2964841 | Ppfibp2 | -1.32 | -1.08 |
| ILMN_1792669 | | HLCS | -1.02 | ILMN_2695867 | Hlcs | -1.15 | -1.08 |
| ILMN_1700727 | | KLF6 | -1.41 | ILMN_1232041 | Klf6 | -0.75 | -1.08 |
| ILMN_1675669 | | IBTK | -1.29 | ILMN_2575324 | Ibtk | -0.87 | -1.08 |
| ILMN_2408730 | | GCC2 | -1.41 | ILMN_3003335 | Gcc2 | -0.74 | -1.08 |
| ILMN_1687351 | | ANKRA2 | -1.16 | ILMN_1243090 | Ankra2 | -0.99 | -1.07 |
| ILMN_1700202 | | TMEM135 | -1.17 | ILMN_2669991 | Tmem135 | -0.96 | -1.07 |
| ILMN_1760933 | | RER1 | -1.36 | ILMN_2935789 | Rer1 | -0.77 | -1.07 |
| ILMN_2346727 | | MTUS1 | -1.35 | ILMN_3163347 | Mtus1 | -0.78 | -1.07 |
| ILMN_1765574 | | TFAP2A | -1.36 | ILMN_2764846 | Tcfap2a | -0.77 | -1.07 |
| ILMN_1794165 | | PGD | -1.36 | ILMN_2712066 | Pgd | -0.77 | -1.07 |
| ILMN_1712634 | | TIA1 | -0.93 | ILMN_1218118 | Tia1 | -1.19 | -1.06 |
| ILMN_2395285 | | SNRNP35 | -1.25 | ILMN_2935386 | Snrnp35 | -0.87 | -1.06 |
| ILMN_1678422 | | DHX58 | -1.27 | ILMN_2915689 | Dhx58 | -0.85 | -1.06 |
| ILMN_1683277 | | KIAA0319L | -1.08 | ILMN_1237034 | AU040320 | -1.02 | -1.05 |
| ILMN_2082314 | | TOM1 | -1.37 | ILMN_2959976 | Tom1 | -0.72 | -1.05 |
| ILMN_1752582 | | RAB5B | -0.94 | ILMN_1237467 | Rab5b | -1.15 | -1.04 |
| ILMN_1811933 | | SHMT1 | -1.01 | ILMN_1238412 | Shmt1 | -1.08 | -1.04 |
| ILMN_1730612 | | DBNDD2 | -0.93 | ILMN_3026137 | Dbndd2 | -1.16 | -1.04 |
| ILMN_1711909 | | EDEM2 | -1.13 | ILMN_2669280 | Edem2 | -0.95 | -1.04 |
| ILMN_1769883 | | IDE | -1.31 | ILMN_1243499 | Ide | -0.76 | -1.03 |
| ILMN_1679600 | | ACOT8 | -1.11 | ILMN_2983587 | Acot8 | -0.96 | -1.03 |
| ILMN_2406656 | | GATA3 | -0.82 | ILMN_1248843 | Gata3 | -1.24 | -1.03 |
| ILMN_2400292 | | MAPK9 | -1.11 | ILMN_2716527 | Mapk9 | -0.95 | -1.03 |
| ILMN_1770940 | | CDH1 | -1.39 | ILMN_2628629 | Cdh1 | -0.67 | -1.03 |
| ILMN_2344204 | | PRR13 | -1.36 | ILMN_2657516 | Prr13 | -0.69 | -1.03 |
| ILMN_1796595 | | C5orf22 | -0.88 | ILMN_1234196 | 6030458C11Rik | -1.17 | -1.02 |
| ILMN_1675239 | | NDUFA7 | -1.18 | ILMN_2629044 | Ndufa7 | -0.87 | -1.02 |
| ILMN_1753498 | | COASY | -1.07 | ILMN_2741236 | Coasy | -0.98 | -1.02 |
| ILMN_2049693 | | C11orf71 | -1.14 | ILMN_2546510 | Gm5617 | -0.90 | -1.02 |
| ILMN_1765000 | | ZSCAN20 | -1.35 | ILMN_2441811 | Zscan20 | -0.69 | -1.02 |
| ILMN_1752665 | | ELMO3 | -0.90 | ILMN_1219244 | Elmo3 | -1.14 | -1.02 |
| ILMN_2323427 | | EPB41 | -1.06 | ILMN_2720813 | Epb4.1 | -0.97 | -1.02 |
| ILMN_1698231 | | RRM2B | -1.24 | ILMN_2994744 | Rrm2b | -0.79 | -1.02 |
| ILMN_1761262 | | MPI | -0.98 | ILMN_2724022 | Mpi | -1.05 | -1.01 |
| ILMN_1754912 | | GLE1 | -1.21 | ILMN_2617810 | Gle1 | -0.82 | -1.01 |
| ILMN_1706764 | | GOLPH3L | -1.27 | ILMN_1235179 | Golph3l | -0.75 | -1.01 |
| ILMN_1749180 | | ESRP1 | -1.26 | ILMN_2947559 | Esrp1 | -0.75 | -1.00 |
| ILMN_1707634 | | CMC1 | -1.16 | ILMN_2756628 | Cmc1 | -0.84 | -1.00 |
| ILMN_2098743 | | ACOT13 | -1.12 | ILMN_2961626 | Acot13 | -0.86 | -0.99 |
| ILMN_1689578 | | TLR3 | -1.04 | ILMN_2697002 | Tlr3 | -0.95 | -0.99 |
| ILMN_1810147 | | ZNF524 | -1.06 | ILMN_2438078 | Zfp524 | -0.92 | -0.99 |
| ILMN_1706598 | | ACPL2 | -0.91 | ILMN_1254634 | Acpl2 | -1.07 | -0.99 |
| ILMN_1690040 | | TM7SF2 | -1.26 | ILMN_1241333 | Tm7sf2 | -0.72 | -0.99 |
| ILMN_1756006 | | ATG2A | -1.16 | ILMN_1213779 | Atg2a | -0.81 | -0.99 |
| ILMN_1808783 | | STRBP | -0.82 | ILMN_1231849 | Strbp | -1.15 | -0.98 |
| ILMN_1659857 | | SNAP29 | -1.20 | ILMN_2730767 | Snap29 | -0.77 | -0.98 |
| ILMN_1747146 | | TSG101 | -1.10 | ILMN_2452237 | Tsg101 | -0.85 | -0.98 |
| ILMN_2150654 | | ZSWIM4 | -0.74 | ILMN_2457408 | Zswim4 | -1.21 | -0.97 |
| ILMN_1711408 | | ANXA4 | -0.92 | ILMN_2935012 | Anxa4 | -1.02 | -0.97 |
| ILMN_1771697 | | VRK3 | -1.18 | ILMN_3160881 | Vrk3 | -0.76 | -0.97 |
| ILMN_1764964 | | IFNGR2 | -1.29 | ILMN_1258300 | Ifngr2 | -0.64 | -0.97 |
| ILMN_1698365 | | NHLRC3 | -0.92 | ILMN_2438516 | Nhlrc3 | -1.01 | -0.96 |
| ILMN_1782079 | | ZFP3 | -0.94 | ILMN_1221886 | Zfp3 | -0.98 | -0.96 |
| ILMN_1681467 | | RAB11FIP4 | -0.78 | ILMN_1236245 | Rab11fip4 | -1.14 | -0.96 |
| ILMN_2361570 | | SNX14 | -1.15 | ILMN_2619249 | Snx14 | -0.77 | -0.96 |
| ILMN_1725787 | | RFX1 | -0.87 | ILMN_2708347 | Rfx1 | -1.05 | -0.96 |
| ILMN_1732489 | | SLC10A7 | -1.21 | ILMN_3162658 | Slc10a7 | -0.69 | -0.95 |
| ILMN_1814856 | | C9orf7 | -1.12 | ILMN_3149218 | 5930434B04Rik | -0.78 | -0.95 |
| ILMN_1710000 | | PEX11G | -0.70 | ILMN_2715661 | Pex11c | -1.19 | -0.95 |
| ILMN_1743806 | | MIF4GD | -1.26 | ILMN_1252144 | Mif4gd | -0.63 | -0.94 |
| ILMN_1682957 | | PACSIN3 | -0.93 | ILMN_2693815 | Pacsin3 | -0.95 | -0.94 |
| ILMN_1798311 | | MBTPS2 | -1.13 | ILMN_2596955 | Mbtps2 | -0.75 | -0.94 |
| ILMN_1885728 | | KIAA1147 | -0.98 | ILMN_2457773 | E330009J07Rik | -0.89 | -0.94 |
| ILMN_1810901 | | RNASEH2A | -1.09 | ILMN_2746721 | Rnaseh2a | -0.78 | -0.94 |
| ILMN_1700660 | | RNF135 | -0.88 | ILMN_2820379 | Rnf135 | -0.99 | -0.93 |
| ILMN_2100815 | | TMEM9B | -0.93 | ILMN_2914884 | Tmem9b | -0.93 | -0.93 |
| ILMN_1798177 | | CHURC1 | -1.07 | ILMN_2797689 | Churc1 | -0.79 | -0.93 |
| ILMN_1687785 | | PPA2 | -0.99 | ILMN_2817996 | Ppa2 | -0.86 | -0.93 |
| ILMN_1710078 | | TMEM181 | -0.75 | ILMN_1245263 | Tmem181a | -1.10 | -0.93 |
| ILMN_1771903 | | NUP37 | -1.15 | ILMN_1220943 | Nup37 | -0.70 | -0.93 |
| ILMN_1735552 | | KIF1B | -1.22 | ILMN_2670778 | Kif1b | -0.64 | -0.93 |
| ILMN_1685413 | | ALG8 | -0.83 | ILMN_1237963 | Alg8 | -1.02 | -0.93 |
| ILMN_1783337 | | DECR2 | -0.86 | ILMN_2705848 | Decr2 | -0.99 | -0.92 |
| ILMN_2380163 | | PTPRF | -0.88 | ILMN_2662690 | Ptprf | -0.96 | -0.92 |
| ILMN_1745374 | | IFI35 | -1.09 | ILMN_2625290 | Ifi35 | -0.74 | -0.92 |
| ILMN_1700549 | | ERLIN2 | -1.20 | ILMN_2816315 | Erlin2 | -0.63 | -0.91 |
| ILMN_1756878 | | SLC39A9 | -0.78 | ILMN_2616772 | Slc39a9 | -1.05 | -0.91 |
| ILMN_1710514 | | BCL3 | -1.07 | ILMN_2749717 | Bcl3 | -0.75 | -0.91 |
| ILMN_1815134 | | PI4K2B | -0.98 | ILMN_2627217 | Pi4k2b | -0.82 | -0.90 |
| ILMN_1752895 | | STX8 | -1.12 | ILMN_2754479 | Stx8 | -0.68 | -0.90 |
| ILMN_1708728 | | H2AFJ | -0.97 | ILMN_1222767 | H2afj | -0.84 | -0.90 |
| ILMN_2378670 | | SNX15 | -0.98 | ILMN_2894450 | Snx15 | -0.82 | -0.90 |
| ILMN_1757995 | | PARP2 | -0.97 | ILMN_2586462 | Parp2 | -0.83 | -0.90 |
| ILMN_1685602 | | TMEM41A | -1.12 | ILMN_1244836 | Tmem41a | -0.66 | -0.89 |
| ILMN_1769040 | | NCOA2 | -0.95 | ILMN_1256574 | Ncoa2 | -0.83 | -0.89 |
| ILMN_2181445 | | BCL2L13 | -0.99 | ILMN_1241307 | Bcl2l13 | -0.78 | -0.89 |
| ILMN_1763144 | | NEU1 | -0.96 | ILMN_2708906 | Neu1 | -0.80 | -0.88 |
| ILMN_2205935 | | SFXN1 | -1.06 | ILMN_2675569 | Sfxn1 | -0.70 | -0.88 |
| ILMN_1759915 | | ARPC1A | -1.06 | ILMN_2699700 | Arpc1a | -0.70 | -0.88 |
| ILMN_1728540 | | FUNDC1 | -1.07 | ILMN_1229913 | Fundc1 | -0.67 | -0.87 |
| ILMN_1743663 | | POMT1 | -0.71 | ILMN_2662545 | Pomt1 | -1.01 | -0.86 |
| ILMN_1753010 | | PET112L | -1.00 | ILMN_1257501 | Pet112l | -0.73 | -0.86 |
| ILMN_1776703 | | TIRAP | -0.69 | ILMN_2452219 | Tirap | -1.03 | -0.86 |
| ILMN_1757317 | | LARS | -1.04 | ILMN_2544674 | Lars | -0.67 | -0.86 |
| ILMN_1731596 | | AP3S2 | -0.86 | ILMN_1244074 | Ap3s2 | -0.84 | -0.85 |
| ILMN_1687626 | | ZDHHC24 | -0.88 | ILMN_2697127 | Zdhhc24 | -0.81 | -0.85 |
| ILMN_1767894 | | POLB | -0.87 | ILMN_2789425 | Polb | -0.81 | -0.84 |
| ILMN_1671902 | | THUMPD3 | -0.68 | ILMN_2809988 | Thumpd3 | -1.00 | -0.84 |
| ILMN_1798485 | | ATP6V1E1 | -1.01 | ILMN_2663872 | Atp6v1e1 | -0.67 | -0.84 |
| ILMN_1703791 | | ANXA7 | -0.94 | ILMN_2561575 | Anxa7 | -0.73 | -0.83 |
| ILMN_1694491 | | CCNG1 | -1.03 | ILMN_2500276 | Ccng1 | -0.63 | -0.83 |
| ILMN_1655765 | | MRPS21 | -0.98 | ILMN_2745555 | Mrps21 | -0.67 | -0.83 |
| ILMN_1657139 | | ADAT1 | -0.90 | ILMN_1256624 | Adat1 | -0.73 | -0.81 |
| ILMN_1766814 | | TK2 | -0.78 | ILMN_1214486 | Tk2 | -0.84 | -0.81 |
| ILMN_1782488 | | RNASEH2B | -0.78 | ILMN_2622209 | Rnaseh2b | -0.83 | -0.81 |
| ILMN_1762666 | | DHRS4 | -0.83 | ILMN_2658054 | Dhrs4 | -0.77 | -0.80 |
| ILMN_2079386 | | RPL22 | -0.89 | ILMN_2659229 | Rpl22 | -0.71 | -0.80 |
| ILMN_1721093 | | TAF10 | -0.84 | ILMN_2995129 | Taf10 | -0.74 | -0.79 |
| ILMN_2379931 | | PIGO | -0.83 | ILMN_2997002 | Pigo | -0.74 | -0.79 |
| ILMN_1727309 | | FAM82A2 | -0.84 | ILMN_3150536 | Fam82a2 | -0.73 | -0.79 |
| ILMN_1793966 | | BOLA1 | -0.79 | ILMN_2640140 | Bola1 | -0.78 | -0.78 |
| ILMN_1737146 | | TRAM1 | -0.79 | ILMN_2509139 | Tram1 | -0.78 | -0.78 |
| ILMN_1754211 | | DCLRE1C | -0.72 | ILMN_1240123 | Dclre1c | -0.84 | -0.78 |
| ILMN_1747577 | | ALAD | -0.82 | ILMN_2655015 | Alad | -0.73 | -0.78 |
| ILMN_1718808 | | AKAP10 | -0.62 | ILMN_2572969 | Akap10 | -0.93 | -0.78 |
| ILMN_1653718 | | ZFAND2B | -0.82 | ILMN_2659440 | Zfand2b | -0.72 | -0.77 |
| ILMN_1750092 | | SEPSECS | -0.69 | ILMN_2950270 | Sepsecs | -0.85 | -0.77 |
| ILMN_1683916 | | PEX13 | -0.68 | ILMN_1254990 | Pex13 | -0.85 | -0.77 |
| ILMN_1773716 | | MRPL9 | -0.89 | ILMN_2534975 | Mrpl9 | -0.64 | -0.76 |
| ILMN_2387175 | | WDR31 | -0.82 | ILMN_1258609 | Wdr31 | -0.69 | -0.76 |
| ILMN_2330243 | | NUDT1 | -0.85 | ILMN_2880623 | Nudt1 | -0.66 | -0.76 |
| ILMN_1783675 | | ASB8 | -0.84 | ILMN_1225252 | Asb8 | -0.67 | -0.76 |
| ILMN_1764261 | | TMEM128 | -0.89 | ILMN_2674032 | Tmem128 | -0.62 | -0.76 |
| ILMN_1758658 | | FADD | -0.75 | ILMN_2662509 | Fadd | -0.75 | -0.75 |
| ILMN_1699610 | | CCT6B | -0.75 | ILMN_2730840 | Cct6b | -0.74 | -0.74 |
| ILMN_1763198 | | STAT6 | -0.89 | ILMN_2897230 | Stat6 | -0.60 | -0.74 |
| ILMN_1751234 | | C1GALT1C1 | -0.88 | ILMN_2682271 | C1galt1c1 | -0.60 | -0.74 |
| ILMN_2361807 | | OS9 | -0.73 | ILMN_1246758 | Os9 | -0.75 | -0.74 |
| ILMN_1768197 | | ROD1 | -0.74 | ILMN_1257327 | Rod1 | -0.73 | -0.73 |
| ILMN_2370907 | | RAD51L3 | -0.68 | ILMN_2755140 | Rad51l3 | -0.78 | -0.73 |
| ILMN_1808792 | | ALKBH6 | -0.76 | ILMN_2720092 | Alkbh6 | -0.69 | -0.72 |
| ILMN_1778611 | | GBAS | -0.65 | ILMN_2703585 | Gbas | -0.78 | -0.72 |
| ILMN_1806017 | | PSME1 | -0.74 | ILMN_2986309 | Psme1 | -0.69 | -0.71 |
| ILMN_2387731 | | NDUFV3 | -0.67 | ILMN_2668977 | Ndufv3 | -0.69 | -0.68 |
| ILMN_1729294 | | RNF130 | -0.70 | ILMN_1249569 | Rnf130 | -0.64 | -0.67 |
| ILMN_2102787 | | HDAC10 | -0.65 | ILMN_2731340 | Hdac10 | -0.68 | -0.66 |
| ILMN_1801941 | | C1orf50 | -0.66 | ILMN_1249278 | AU022252 | -0.65 | -0.65 |
| ILMN_1788625 | | EXOC5 | -0.65 | ILMN_1233365 | Exoc5 | -0.63 | -0.64 |

The conserved genes between mouse and human were selected by using the nested F multiple testing adjustments with FDR<0.1 and at least 1.5 fold change. Mouse signature genes for a subset were first selected, then multiple testing adjustments were performed for the human data of these subsets of the ortholog genes. The mouse signature genes that were also significantly differentially expressed in human were defined as the conserved genes. The conserved genes represent those consistently up or down in one subpopulation across the two species.
