# Supplementary material for: Transcriptome analyses of mouse and human mammary cell subpopulations reveal multiple conserved genes and pathways
Source: Breast Cancer Res. 2010 Mar 26;12(2):R21. doi: 10.1186/bcr2560 (PMC2879567; doi:10.1186/bcr2560)
Supplement: Additional file 5 — Supplementary Table 2 containing conserved genes in the luminal progenitor subset. The table gives the 58 genes which are up-regulated and the 14 genes which are down-regulated in the luminal progenitor subset in both species. [file bcr2560-S5.DOC]

**Supplementary Table 2: Conserved genes in the luminal progenitor subset**

| **Up-regulated in luminal progenitor (LP) cells** | | | | | | |
| --- | --- | --- | --- | --- | --- | --- |
| ID human | symbol | log Fold Change | ID mouse | symbol | log Fold Change | average  log Fold  Change |
| ILMN_1728255 | LALBA | 3.76 | ILMN_2676107 | Lalba | 3.46 | 3.61 |
| ILMN_2184109 | SLC34A2 | 4.74 | ILMN_2699611 | Slc34a2 | 2.21 | 3.47 |
| ILMN_1792400 | CSN3 | 4.77 | ILMN_1227118 | Csn3 | 2.08 | 3.43 |
| ILMN_1685663 | CYP24A1 | 3.68 | ILMN_2639900 | Cyp24a1 | 3.17 | 3.42 |
| ILMN_1685387 | PIGR | 5.00 | ILMN_1225605 | Pigr | 1.61 | 3.31 |
| ILMN_2229379 | KIT | 4.13 | ILMN_1246876 | Kit | 2.13 | 3.13 |
| ILMN_1813270 | ELF5 | 3.90 | ILMN_2736380 | Elf5 | 2.27 | 3.09 |
| ILMN_1788538 | NCALD | 3.44 | ILMN_2652414 | Ncald | 2.66 | 3.05 |
| ILMN_1729801 | S100A8 | 3.12 | ILMN_2710905 | S100a8 | 2.88 | 3.00 |
| ILMN_1661733 | FOLR1 | 2.98 | ILMN_2707541 | Folr1 | 2.96 | 2.97 |
| ILMN_1741014 | SLC28A3 | 3.20 | ILMN_2634129 | Slc28a3 | 2.63 | 2.92 |
| ILMN_1732538 | LBP | 3.43 | ILMN_2771237 | Lbp | 2.02 | 2.73 |
| ILMN_1812073 | ATP6V1B1 | 2.66 | ILMN_2668432 | Atp6v1b1 | 2.78 | 2.72 |
| ILMN_1708580 | PDZK1IP1 | 3.72 | ILMN_2618935 | Pdzk1ip1 | 1.63 | 2.67 |
| ILMN_1716407 | SORBS2 | 3.63 | ILMN_1235808 | Sorbs2 | 1.44 | 2.53 |
| ILMN_1724686 | CLDN1 | 2.98 | ILMN_2870295 | Cldn1 | 2.06 | 2.52 |
| ILMN_1769388 | GJB2 | 2.89 | ILMN_2999627 | Gjb2 | 2.14 | 2.51 |
| ILMN_1681544 | RASGEF1C | 1.83 | ILMN_1231901 | Rasgef1c | 3.18 | 2.51 |
| ILMN_2308903 | WFDC3 | 1.92 | ILMN_1229131 | Wfdc3 | 2.91 | 2.42 |
| ILMN_1659960 | IL4I1 | 3.08 | ILMN_2733778 | Il4i1 | 1.60 | 2.34 |
| ILMN_1763837 | ANPEP | 2.89 | ILMN_2589651 | Anpep | 1.61 | 2.25 |
| ILMN_1762260 | C3 | 3.15 | ILMN_2759484 | C3 | 1.19 | 2.17 |
| ILMN_1736936 | FOXI1 | 1.64 | ILMN_2722232 | Foxi1 | 2.68 | 2.16 |
| ILMN_1749403 | TSPAN33 | 2.04 | ILMN_1219904 | Tspan33 | 2.24 | 2.14 |
| ILMN_1712632 | XDH | 2.31 | ILMN_1239055 | Xdh | 1.84 | 2.07 |
| ILMN_1727689 | TNFAIP2 | 2.22 | ILMN_2841289 | Tnfaip2 | 1.93 | 2.07 |
| ILMN_1734929 | BBOX1 | 2.56 | ILMN_2686029 | Bbox1 | 1.23 | 1.90 |
| ILMN_2259818 | MFI2 | 1.44 | ILMN_1254745 | Mfi2 | 2.33 | 1.88 |
| ILMN_1792744 | SLC13A2 | 0.76 | ILMN_2646369 | Slc13a2 | 2.96 | 1.86 |
| ILMN_1793517 | RASAL1 | 2.16 | ILMN_1247762 | Rasal1 | 1.52 | 1.84 |
| ILMN_1801584 | CXCR4 | 1.90 | ILMN_2630459 | Cxcr4 | 1.71 | 1.81 |
| ILMN_2380237 | C1QTNF1 | 2.34 | ILMN_2722108 | C1qtnf1 | 1.25 | 1.80 |
| ILMN_2396444 | CD14 | 1.49 | ILMN_2742075 | Cd14 | 2.05 | 1.77 |
| ILMN_1654319 | HAPLN3 | 2.10 | ILMN_1231791 | Hapln3 | 1.25 | 1.68 |
| ILMN_1769694 | ACCN2 | 1.71 | ILMN_2643241 | Accn2 | 1.62 | 1.66 |
| ILMN_1736929 | PLB1 | 0.81 | ILMN_2674163 | Plb1 | 2.50 | 1.65 |
| ILMN_1813104 | GALNTL2 | 1.90 | ILMN_1244612 | Galntl2 | 1.37 | 1.63 |
| ILMN_1660729 | ATP6V1C2 | 1.56 | ILMN_2898578 | Atp6v1c2 | 1.59 | 1.58 |
| ILMN_2139970 | ALDH1A3 | 1.29 | ILMN_1237578 | Aldh1a3 | 1.72 | 1.50 |
| ILMN_1652277 | SECTM1 | 0.91 | ILMN_2656108 | Sectm1a | 1.95 | 1.43 |
| ILMN_1741727 | QPCT | 1.83 | ILMN_2814865 | Qpct | 0.98 | 1.40 |
| ILMN_1684585 | ACSL1 | 1.32 | ILMN_2622671 | Acsl1 | 1.39 | 1.35 |
| ILMN_1680367 | C10orf90 | 1.97 | ILMN_1255809 | D7Ertd443e | 0.68 | 1.32 |
| ILMN_1667125 | RPS6KL1 | 1.18 | ILMN_2649333 | Rps6kl1 | 1.45 | 1.32 |
| ILMN_2242463 | CTSC | 1.63 | ILMN_3008858 | Ctsc | 0.95 | 1.29 |
| ILMN_1751596 | HIVEP3 | 1.24 | ILMN_2466021 | Hivep3 | 1.23 | 1.24 |
| ILMN_1801441 | RFTN2 | 1.17 | ILMN_1212653 | Rftn2 | 1.30 | 1.24 |
| ILMN_1763491 | CKMT1B | 1.12 | ILMN_2773537 | Ckmt1 | 1.33 | 1.22 |
| ILMN_1749372 | GGT5 | 1.17 | ILMN_2698271 | Ggt5 | 1.24 | 1.20 |
| ILMN_1724181 | IL15 | 1.53 | ILMN_2938704 | Il15 | 0.78 | 1.15 |
| ILMN_1711579 | CSN2 | 0.63 | ILMN_2621101 | Csn2 | 1.67 | 1.15 |
| ILMN_1772208 | CCDC88B | 1.10 | ILMN_2737463 | Ccdc88b | 1.18 | 1.14 |
| ILMN_2363450 | NOXO1 | 1.04 | ILMN_2683856 | Noxo1 | 1.23 | 1.13 |
| ILMN_2097410 | DAPP1 | 0.78 | ILMN_2595822 | Dapp1 | 1.29 | 1.03 |
| ILMN_1729417 | GNE | 1.16 | ILMN_1216440 | Gne | 0.90 | 1.03 |
| ILMN_1736103 | ITPR2 | 1.37 | ILMN_3076170 | Itpr2 | 0.67 | 1.02 |
| ILMN_2094106 | HSD17B12 | 0.82 | ILMN_1234796 | Hsd17b12 | 1.05 | 0.93 |
| ILMN_1702171 | LPCAT1 | 0.72 | ILMN_2995575 | Lpcat1 | 0.89 | 0.81 |
| **Down-regulated in luminal progenitor (LP) cells** | | | | | | |
| ID human | symbol | log Fold Change | ID mouse | symbol | log Fold Change | average  log Fold  Change |
| ILMN_1791280 | HSPB8 | -2.84 | ILMN_2944366 | Hspb8 | -1.61 | -2.23 |
| ILMN_1655405 | SCARF2 | -2.88 | ILMN_2676127 | Scarf2 | -1.49 | -2.19 |
| ILMN_1675797 | EPDR1 | -2.45 | ILMN_1225494 | Epdr1 | -1.78 | -2.11 |
| ILMN_1703913 | DST | -2.68 | ILMN_2758234 | Dst | -0.97 | -1.82 |
| ILMN_1657766 | TRIM29 | -1.78 | ILMN_1218207 | Trim29 | -1.83 | -1.81 |
| ILMN_1792679 | ITGA5 | -2.30 | ILMN_2849449 | Itga5 | -1.26 | -1.78 |
| ILMN_2061565 | PLCH2 | -1.66 | ILMN_2925433 | Plch2 | -1.47 | -1.56 |
| ILMN_1767662 | LASS6 | -2.25 | ILMN_2678580 | Lass6 | -0.82 | -1.54 |
| ILMN_1744403 | KCNIP3 | -0.93 | ILMN_2911729 | Kcnip3 | -1.76 | -1.34 |
| ILMN_1761540 | SEMA3F | -1.55 | ILMN_1248740 | Sema3f | -1.06 | -1.30 |
| ILMN_2352934 | ASPH | -1.18 | ILMN_1233759 | Asph | -1.30 | -1.24 |
| ILMN_1797277 | KIF3C | -1.19 | ILMN_2593410 | Kif3c | -0.98 | -1.09 |
| ILMN_1703891 | TBC1D9 | -1.38 | ILMN_1228165 | Tbc1d9 | -0.69 | -1.03 |
| ILMN_2278636 | CUX1 | -0.88 | ILMN_1223862 | Cux1 | -0.73 | -0.80 |

The conserved genes between mouse and human were selected by using the nested F multiple testing adjustments with FDR<0.1 and at least 1.5 fold change. Mouse signature genes for a subset were first selected, then multiple testing adjustments were performed for the human data of these subsets of the ortholog genes. The mouse signature genes that were also significantly differentially expressed in human were defined as the conserved genes. The conserved genes represent those consistently up or down in one subpopulation across the two species.
