# Supplementary material for: Transcriptome analyses of mouse and human mammary cell subpopulations reveal multiple conserved genes and pathways
Source: Breast Cancer Res. 2010 Mar 26;12(2):R21. doi: 10.1186/bcr2560 (PMC2879567; doi:10.1186/bcr2560)
Supplement: Additional file 6 — Supplementary Table 3 containing conserved genes in the mature luminal subset. The table gives the 116 genes which are up-regulated and the 99 genes which are down-regulated in the mature luminal subset in both species. [file bcr2560-S6.DOC]

**Supplementary Table 3: Conserved genes in the mature luminal subset**

| **Up-regulated in mature luminal (ML) cells** | | | | | | |
| --- | --- | --- | --- | --- | --- | --- |
| ID human | symbol | log Fold Change | ID mouse | symbol | log Fold Change | average  log Fold  Change |
| ILMN_1766650 | FOXA1 | 4.85 | ILMN_1237195 | Foxa1 | 2.90 | 3.87 |
| ILMN_2269256 | DNAJC12 | 4.44 | ILMN_1229993 | Dnajc12 | 2.57 | 3.51 |
| ILMN_1795342 | MLPH | 5.30 | ILMN_3162347 | Mlph | 1.67 | 3.48 |
| ILMN_1787266 | SPINK1 | 4.69 | ILMN_2708477 | Spink3 | 2.05 | 3.37 |
| ILMN_1779517 | RASEF | 4.55 | ILMN_2950343 | Rasef | 2.14 | 3.34 |
| ILMN_1668822 | BATF | 4.84 | ILMN_2761900 | Batf | 1.45 | 3.15 |
| ILMN_2413833 | TOX3 | 4.09 | ILMN_2434900 | Tox3 | 2.13 | 3.11 |
| ILMN_1815203 | HMGCS2 | 3.51 | ILMN_1216322 | Hmgcs2 | 2.58 | 3.05 |
| ILMN_2108735 | EEF1A2 | 5.13 | ILMN_2971559 | Eef1a2 | 0.85 | 2.99 |
| ILMN_1691641 | CITED1 | 2.90 | ILMN_1213474 | Cited1 | 3.05 | 2.98 |
| ILMN_1767129 | ABCC8 | 3.59 | ILMN_2645526 | Abcc8 | 2.23 | 2.91 |
| ILMN_1669781 | PRLR | 3.22 | ILMN_2868699 | Prlr | 2.59 | 2.90 |
| ILMN_1781400 | SLC7A2 | 3.65 | ILMN_2642349 | Slc7a2 | 2.07 | 2.86 |
| ILMN_1755649 | SLC16A5 | 2.57 | ILMN_2461172 | Slc16a5 | 2.87 | 2.72 |
| ILMN_1763666 | ALDH3B2 | 4.01 | ILMN_2746937 | Aldh3b2 | 1.18 | 2.59 |
| ILMN_2053103 | SLC40A1 | 3.58 | ILMN_2747923 | Slc40a1 | 1.42 | 2.50 |
| ILMN_1666392 | WNT4 | 2.20 | ILMN_2889641 | Wnt4 | 2.80 | 2.50 |
| ILMN_2161330 | SPDEF | 2.79 | ILMN_1258162 | Spdef | 2.17 | 2.48 |
| ILMN_1697460 | REEP6 | 2.98 | ILMN_2661650 | Reep6 | 1.95 | 2.46 |
| ILMN_1727589 | SULT2B1 | 2.90 | ILMN_2686353 | Sult2b1 | 1.94 | 2.42 |
| ILMN_1811014 | PGR | 2.22 | ILMN_2657452 | Pgr | 2.30 | 2.26 |
| ILMN_2069224 | PVALB | 1.59 | ILMN_1218223 | Pvalb | 2.89 | 2.24 |
| ILMN_1764082 | MBOAT1 | 2.03 | ILMN_1225029 | Mboat1 | 2.33 | 2.18 |
| ILMN_1673175 | TNFSF11 | 1.25 | ILMN_2505780 | Tnfsf11 | 3.07 | 2.16 |
| ILMN_1705750 | TGM2 | 3.10 | ILMN_2570977 | Tgm2 | 1.21 | 2.15 |
| ILMN_2204876 | FLVCR2 | 2.85 | ILMN_1248190 | Mfsd7c | 1.41 | 2.13 |
| ILMN_1810915 | FAAH | 1.51 | ILMN_2657980 | Faah | 2.73 | 2.12 |
| ILMN_2382505 | SLC22A18 | 1.96 | ILMN_3152079 | Slc22a18 | 2.17 | 2.07 |
| ILMN_1678535 | ESR1 | 2.39 | ILMN_2726412 | Esr1 | 1.69 | 2.04 |
| ILMN_1711894 | MYB | 2.44 | ILMN_2683910 | Myb | 1.59 | 2.01 |
| ILMN_1669114 | WNK4 | 2.03 | ILMN_2518744 | Wnk4 | 1.92 | 1.98 |
| ILMN_2366192 | FGL1 | 2.94 | ILMN_1242370 | Fgl1 | 0.86 | 1.90 |
| ILMN_1654013 | C17orf28 | 2.11 | ILMN_3055445 | C630004H02Rik | 1.66 | 1.89 |
| ILMN_1670870 | ALCAM | 1.87 | ILMN_2606804 | Alcam | 1.83 | 1.85 |
| ILMN_1813100 | KIAA1244 | 1.66 | ILMN_2781181 | D10Bwg1379e | 2.00 | 1.83 |
| ILMN_1747546 | TSPAN1 | 1.54 | ILMN_2624180 | Tspan1 | 2.10 | 1.82 |
| ILMN_2206722 | FER1L4 | 1.27 | ILMN_2724298 | Fer1l4 | 2.28 | 1.77 |
| ILMN_1800317 | WNT5A | 2.32 | ILMN_2511913 | Wnt5a | 1.19 | 1.76 |
| ILMN_1713449 | TBX3 | 2.02 | ILMN_3139514 | Tbx3 | 1.49 | 1.75 |
| ILMN_1780671 | PLEKHG3 | 2.43 | ILMN_1221920 | Plekhg3 | 0.98 | 1.70 |
| ILMN_1707077 | SORT1 | 1.67 | ILMN_2685772 | Sort1 | 1.68 | 1.68 |
| ILMN_1670064 | C1orf210 | 1.25 | ILMN_1221084 | 2610528J11Rik | 2.01 | 1.63 |
| ILMN_1731107 | CCDC92 | 1.09 | ILMN_2598374 | Ccdc92 | 2.15 | 1.62 |
| ILMN_2274775 | SLC44A4 | 0.68 | ILMN_2970023 | Slc44a4 | 2.54 | 1.61 |
| ILMN_1716591 | SPRR1A | 1.41 | ILMN_2975250 | Sprr1a | 1.75 | 1.58 |
| ILMN_1785095 | ATP6V0E2 | 1.57 | ILMN_2832658 | Atp6v0e2 | 1.51 | 1.54 |
| ILMN_1792168 | GALE | 1.26 | ILMN_1237990 | Gale | 1.81 | 1.53 |
| ILMN_1656910 | TRIM6 | 1.51 | ILMN_1255191 | Trim6 | 1.55 | 1.53 |
| ILMN_1669881 | TSPAN13 | 2.08 | ILMN_1231147 | Tspan13 | 0.91 | 1.49 |
| ILMN_1761946 | PROM2 | 1.46 | ILMN_1246392 | Prom2 | 1.52 | 1.49 |
| ILMN_1806456 | C14orf45 | 2.30 | ILMN_2948800 | 2900006K08Rik | 0.67 | 1.49 |
| ILMN_2153495 | WNT7B | 0.91 | ILMN_2789692 | Wnt7b | 2.04 | 1.47 |
| ILMN_2154115 | PSD4 | 1.82 | ILMN_2867382 | Psd4 | 1.12 | 1.47 |
| ILMN_1655191 | CASZ1 | 1.00 | ILMN_2665095 | Casz1 | 1.92 | 1.46 |
| ILMN_2401873 | DUSP10 | 2.05 | ILMN_2678724 | Dusp10 | 0.81 | 1.43 |
| ILMN_1749044 | PVRL4 | 1.85 | ILMN_1235878 | Pvrl4 | 0.98 | 1.42 |
| ILMN_1812503 | PHKA1 | 1.89 | ILMN_2712685 | Phka1 | 0.90 | 1.39 |
| ILMN_2352090 | GPRC5C | 2.03 | ILMN_3127190 | Gprc5c | 0.74 | 1.39 |
| ILMN_1701007 | PON3 | 1.82 | ILMN_1244130 | Pon3 | 0.92 | 1.37 |
| ILMN_1684690 | HDAC11 | 1.04 | ILMN_2614912 | Hdac11 | 1.66 | 1.35 |
| ILMN_1662359 | HIST1H4K | 1.43 | ILMN_1243654 | Hist1h4k | 1.24 | 1.34 |
| ILMN_1706687 | KLHL5 | 1.66 | ILMN_2944870 | Klhl5 | 0.99 | 1.33 |
| ILMN_1741371 | TMEM8A | 1.69 | ILMN_3008238 | Tmem8 | 0.93 | 1.31 |
| ILMN_1738675 | PTPN6 | 1.38 | ILMN_1220996 | Ptpn6 | 1.23 | 1.31 |
| ILMN_1653826 | FGF13 | 0.88 | ILMN_2745480 | Fgf13 | 1.70 | 1.29 |
| ILMN_1651498 | GADD45G | 1.72 | ILMN_2744890 | Gadd45g | 0.84 | 1.28 |
| ILMN_1652602 | C11orf35 | 1.20 | ILMN_1242629 | 1600016N20Rik | 1.28 | 1.24 |
| ILMN_1774229 | SLC7A4 | 0.69 | ILMN_2639360 | Slc7a4 | 1.77 | 1.23 |
| ILMN_1768505 | IL13RA1 | 1.20 | ILMN_2668696 | Il13ra1 | 1.20 | 1.20 |
| ILMN_1709032 | FYCO1 | 1.43 | ILMN_2544597 | Fyco1 | 0.96 | 1.20 |
| ILMN_1654946 | ZSCAN18 | 1.06 | ILMN_2531903 | Zscan18 | 1.31 | 1.18 |
| ILMN_1662306 | RABL3 | 1.48 | ILMN_1216900 | Rabl3 | 0.87 | 1.18 |
| ILMN_1779997 | MEIS3 | 1.54 | ILMN_3126848 | Meis3 | 0.80 | 1.17 |
| ILMN_1755721 | FAM63A | 1.36 | ILMN_2630827 | Fam63a | 0.98 | 1.17 |
| ILMN_1694268 | HES6 | 1.10 | ILMN_1214126 | Hes6 | 1.22 | 1.16 |
| ILMN_2195482 | CACNB3 | 1.62 | ILMN_3153753 | Cacnb3 | 0.70 | 1.16 |
| ILMN_2347949 | G6PD | 0.90 | ILMN_2721337 | G6pdx | 1.40 | 1.15 |
| ILMN_1728662 | ALDH3B1 | 0.71 | ILMN_2645793 | Aldh3b1 | 1.57 | 1.14 |
| ILMN_1671600 | EPS8L1 | 0.85 | ILMN_2720150 | Eps8l1 | 1.43 | 1.14 |
| ILMN_1702124 | LNX2 | 1.58 | ILMN_2598990 | Lnx2 | 0.70 | 1.14 |
| ILMN_2220739 | TMCO3 | 1.16 | ILMN_2628745 | Tmco3 | 1.11 | 1.14 |
| ILMN_1772074 | C19orf51 | 1.22 | ILMN_2959253 | 6030429G01Rik | 1.04 | 1.13 |
| ILMN_1686906 | TP53INP2 | 0.94 | ILMN_2457585 | Trp53inp2 | 1.31 | 1.13 |
| ILMN_1756358 | FBXO36 | 1.10 | ILMN_2717387 | Fbxo36 | 1.14 | 1.12 |
| ILMN_1789405 | C22orf25 | 1.23 | ILMN_2768364 | D16H22S680E | 0.96 | 1.09 |
| ILMN_1740505 | SGMS1 | 0.97 | ILMN_2480682 | Sgms1 | 1.19 | 1.08 |
| ILMN_1810274 | HOXB2 | 1.35 | ILMN_2598083 | Hoxb2 | 0.82 | 1.08 |
| ILMN_1698404 | ERN1 | 0.98 | ILMN_2502860 | Ern1 | 1.17 | 1.07 |
| ILMN_1687440 | HIPK2 | 0.90 | ILMN_1233987 | Hipk2 | 1.23 | 1.07 |
| ILMN_1784292 | ANKMY2 | 1.18 | ILMN_2599514 | Ankmy2 | 0.91 | 1.05 |
| ILMN_1750205 | ZFHX2 | 0.85 | ILMN_2964785 | Zfhx2 | 1.24 | 1.04 |
| ILMN_1773567 | LAMA5 | 1.16 | ILMN_2640248 | Lama5 | 0.90 | 1.03 |
| ILMN_1679949 | SLC25A23 | 0.87 | ILMN_2675672 | Slc25a23 | 1.18 | 1.03 |
| ILMN_1813350 | HSD11B2 | 1.06 | ILMN_2731265 | Hsd11b2 | 0.99 | 1.03 |
| ILMN_1803939 | YIPF6 | 0.66 | ILMN_3159866 | Yipf6 | 1.38 | 1.02 |
| ILMN_1762606 | AQP11 | 0.81 | ILMN_1225901 | Aqp11 | 1.23 | 1.02 |
| ILMN_2353862 | LRRC48 | 1.16 | ILMN_2594644 | Lrrc48 | 0.86 | 1.01 |
| ILMN_1729487 | GMPR | 0.89 | ILMN_2602581 | Gmpr | 1.11 | 1.00 |
| ILMN_2224990 | HIST1H4J | 0.84 | ILMN_1256989 | Hist1h4j | 1.17 | 1.00 |
| ILMN_2306955 | ACPL2 | 0.93 | ILMN_1254634 | Acpl2 | 1.06 | 1.00 |
| ILMN_1702149 | CACNG4 | 0.80 | ILMN_2662214 | Cacng4 | 1.15 | 0.98 |
| ILMN_1739594 | ACOT11 | 0.92 | ILMN_1227579 | Acot11 | 1.02 | 0.97 |
| ILMN_1705685 | MEIS1 | 1.00 | ILMN_1218266 | Meis1 | 0.92 | 0.96 |
| ILMN_1670028 | LPIN2 | 1.06 | ILMN_1220680 | Lpin2 | 0.82 | 0.94 |
| ILMN_1725014 | TMPRSS6 | 0.79 | ILMN_2469310 | Tmprss6 | 1.08 | 0.93 |
| ILMN_1653861 | SCMH1 | 0.95 | ILMN_1230766 | Scmh1 | 0.85 | 0.90 |
| ILMN_1728887 | PAK4 | 0.73 | ILMN_2640570 | Pak4 | 1.05 | 0.89 |
| ILMN_1728024 | TUBG1 | 0.97 | ILMN_2420070 | Tubg1 | 0.78 | 0.88 |
| ILMN_2226955 | VOPP1 | 0.91 | ILMN_2735429 | Vopp1 | 0.83 | 0.87 |
| ILMN_1743205 | ABCA7 | 0.90 | ILMN_2896639 | Abca7 | 0.81 | 0.86 |
| ILMN_2094313 | ZDHHC1 | 0.98 | ILMN_2495299 | Zdhhc1 | 0.72 | 0.85 |
| ILMN_1807379 | WHSC1L1 | 0.70 | ILMN_2583684 | Whsc1l1 | 0.81 | 0.76 |
| ILMN_1779828 | EDEM1 | 0.82 | ILMN_1244297 | Edem1 | 0.67 | 0.75 |
| ILMN_1689438 | BTRC | 0.80 | ILMN_3100042 | Btrc | 0.62 | 0.71 |
| ILMN_1789243 | VPS33B | 0.76 | ILMN_2422410 | Vps33b | 0.63 | 0.70 |
| ILMN_1687092 | KBTBD4 | 0.69 | ILMN_2806291 | Kbtbd4 | 0.67 | 0.68 |
| **Down-regulated in mature luminal (ML) cells** | | | | | | |
| ID human | symbol | log Fold Change | ID mouse | symbol | log Fold Change | average  log Fold  Change |
| ILMN_1696434 | LAMA1 | -3.67 | ILMN_2973288 | Lama1 | -3.31 | -3.49 |
| ILMN_1736178 | AEBP1 | -3.36 | ILMN_2873822 | Aebp1 | -3.26 | -3.31 |
| ILMN_2201678 | FSTL1 | -3.33 | ILMN_2734683 | Fstl1 | -3.02 | -3.17 |
| ILMN_1657373 | LEPREL1 | -4.16 | ILMN_1249635 | Leprel1 | -2.13 | -3.14 |
| ILMN_1724994 | COL4A2 | -3.27 | ILMN_2822579 | Col4a2 | -2.78 | -3.02 |
| ILMN_1746085 | IGFBP3 | -2.68 | ILMN_1219335 | Igfbp3 | -3.24 | -2.96 |
| ILMN_1740938 | APOE | -3.19 | ILMN_1216042 | Apoe | -2.64 | -2.91 |
| ILMN_1653028 | COL4A1 | -3.02 | ILMN_2621643 | Col4a1 | -2.74 | -2.88 |
| ILMN_2082585 | SNAI2 | -2.58 | ILMN_2647563 | Snai2 | -3.16 | -2.87 |
| ILMN_1738552 | SLC1A3 | -2.76 | ILMN_2634317 | Slc1a3 | -2.92 | -2.84 |
| ILMN_2149164 | SFRP1 | -3.38 | ILMN_1231689 | Sfrp1 | -2.29 | -2.83 |
| ILMN_1687501 | MOXD1 | -2.21 | ILMN_2737390 | Moxd1 | -3.40 | -2.80 |
| ILMN_1668039 | GYPC | -3.61 | ILMN_1218358 | Gypc | -1.97 | -2.79 |
| ILMN_1784287 | TGFBR3 | -3.00 | ILMN_2789239 | Tgfbr3 | -2.55 | -2.77 |
| ILMN_1718295 | STAC2 | -2.02 | ILMN_2703563 | Stac2 | -3.48 | -2.75 |
| ILMN_1795251 | SPARCL1 | -3.52 | ILMN_1237917 | Sparcl1 | -1.93 | -2.73 |
| ILMN_1749792 | SORBS1 | -3.05 | ILMN_3027751 | Sorbs1 | -2.40 | -2.72 |
| ILMN_1705066 | BTBD11 | -2.84 | ILMN_3139253 | Btbd11 | -2.49 | -2.67 |
| ILMN_1787526 | C2orf88 | -3.06 | ILMN_2646640 | 1700019D03Rik | -2.22 | -2.64 |
| ILMN_1726030 | GPX7 | -2.48 | ILMN_2724294 | Gpx7 | -2.58 | -2.53 |
| ILMN_1655611 | TSHZ2 | -2.89 | ILMN_1243934 | Tshz2 | -2.15 | -2.52 |
| ILMN_1732151 | COL6A1 | -2.64 | ILMN_2768087 | Col6a1 | -2.37 | -2.51 |
| ILMN_1738147 | NES | -2.98 | ILMN_2703267 | Nes | -1.99 | -2.49 |
| ILMN_2146761 | FABP5 | -2.75 | ILMN_1235908 | Fabp5 | -2.19 | -2.47 |
| ILMN_1651950 | TPST1 | -2.12 | ILMN_1230765 | Tpst1 | -2.76 | -2.44 |
| ILMN_2075757 | LRIG3 | -1.78 | ILMN_1213273 | Lrig3 | -3.05 | -2.42 |
| ILMN_2326512 | CASP1 | -3.24 | ILMN_1247592 | Casp1 | -1.59 | -2.41 |
| ILMN_1730487 | CALD1 | -3.04 | ILMN_1232081 | Cald1 | -1.77 | -2.41 |
| ILMN_1783909 | COL6A2 | -2.45 | ILMN_1216661 | Col6a2 | -2.31 | -2.38 |
| ILMN_1676563 | HTRA1 | -1.79 | ILMN_2746738 | Htra1 | -2.78 | -2.28 |
| ILMN_1806403 | RASL12 | -2.04 | ILMN_2836749 | Rasl12 | -2.30 | -2.17 |
| ILMN_2319077 | FAS | -2.00 | ILMN_2902979 | Fas | -2.32 | -2.16 |
| ILMN_2052891 | PKD2 | -2.22 | ILMN_2866327 | Pkd2 | -2.05 | -2.13 |
| ILMN_1764228 | DAB2 | -2.77 | ILMN_1243329 | Dab2 | -1.45 | -2.11 |
| ILMN_1653750 | SOX10 | -2.90 | ILMN_1228105 | Sox10 | -1.28 | -2.09 |
| ILMN_1798975 | EGFR | -1.75 | ILMN_2693922 | Egfr | -2.35 | -2.05 |
| ILMN_1729216 | CRYAB | -2.87 | ILMN_2840213 | Cryab | -1.20 | -2.04 |
| ILMN_1741356 | PRICKLE1 | -2.39 | ILMN_1228245 | Prickle1 | -1.62 | -2.01 |
| ILMN_1764729 | JAG2 | -1.46 | ILMN_2626585 | Jag2 | -2.55 | -2.01 |
| ILMN_1715068 | AQP9 | -1.55 | ILMN_1214634 | Aqp9 | -2.40 | -1.98 |
| ILMN_1786444 | LPL | -2.36 | ILMN_2692723 | Lpl | -1.55 | -1.96 |
| ILMN_2129234 | TMEM47 | -1.99 | ILMN_3124787 | Tmem47 | -1.92 | -1.96 |
| ILMN_1714067 | NTRK2 | -2.55 | ILMN_3138904 | Ntrk2 | -1.34 | -1.95 |
| ILMN_2291083 | SLC6A15 | -1.03 | ILMN_1258914 | Slc6a15 | -2.84 | -1.93 |
| ILMN_1782538 | VIM | -1.61 | ILMN_2451022 | Vim | -2.22 | -1.92 |
| ILMN_1687538 | ETS1 | -2.16 | ILMN_3157483 | Ets1 | -1.65 | -1.91 |
| ILMN_1684554 | COL16A1 | -1.73 | ILMN_1248099 | Col16a1 | -2.06 | -1.89 |
| ILMN_2103761 | TLE4 | -2.63 | ILMN_2589792 | Tle4 | -1.15 | -1.89 |
| ILMN_2386291 | WTIP | -1.78 | ILMN_2424268 | Wtip | -2.00 | -1.89 |
| ILMN_1683456 | CCL7 | -1.94 | ILMN_2835117 | Ccl7 | -1.81 | -1.87 |
| ILMN_1675507 | AKAP2 | -2.06 | ILMN_1249197 | Akap2 | -1.67 | -1.87 |
| ILMN_1784749 | GAS6 | -2.23 | ILMN_2686327 | Gas6 | -1.49 | -1.86 |
| ILMN_2358560 | TIAM2 | -2.15 | ILMN_2836875 | Tiam2 | -1.55 | -1.85 |
| ILMN_1672503 | DPYSL2 | -1.67 | ILMN_2949596 | Dpysl2 | -2.00 | -1.84 |
| ILMN_1665219 | LTBP4 | -1.51 | ILMN_2829262 | Ltbp4 | -2.14 | -1.83 |
| ILMN_1752899 | BCL11A | -2.65 | ILMN_1257463 | Bcl11a | -0.88 | -1.76 |
| ILMN_1717046 | MOBKL2B | -2.26 | ILMN_2900854 | Mobkl2b | -1.24 | -1.75 |
| ILMN_1703955 | FBXO32 | -1.34 | ILMN_2873444 | Fbxo32 | -2.14 | -1.74 |
| ILMN_1680738 | C5orf13 | -2.02 | ILMN_2680054 | D0H4S114 | -1.42 | -1.72 |
| ILMN_1791847 | DAPK2 | -1.50 | ILMN_2977558 | Dapk2 | -1.88 | -1.69 |
| ILMN_1682034 | HEY2 | -2.01 | ILMN_2657207 | Hey2 | -1.36 | -1.69 |
| ILMN_1758128 | CYGB | -1.69 | ILMN_2801891 | Cygb | -1.65 | -1.67 |
| ILMN_1735502 | FAM181B | -1.77 | ILMN_1234519 | Fam181b | -1.55 | -1.66 |
| ILMN_1805448 | EPB41L2 | -1.22 | ILMN_2488835 | Epb4.1l2 | -2.09 | -1.66 |
| ILMN_1742332 | KCTD12 | -2.34 | ILMN_2736783 | Kctd12 | -0.96 | -1.65 |
| ILMN_1654586 | RASA3 | -0.88 | ILMN_2637714 | Rasa3 | -2.39 | -1.64 |
| ILMN_1751028 | SERPINH1 | -1.85 | ILMN_2777359 | Serpinh1 | -1.39 | -1.62 |
| ILMN_2138589 | MERTK | -2.21 | ILMN_2714638 | Mertk | -1.01 | -1.61 |
| ILMN_1807169 | TINAGL1 | -1.61 | ILMN_2976129 | Tinagl1 | -1.60 | -1.60 |
| ILMN_1688630 | RECK | -1.41 | ILMN_2812614 | Reck | -1.73 | -1.57 |
| ILMN_1678961 | FRMD4A | -1.86 | ILMN_1215203 | Frmd4a | -1.26 | -1.56 |
| ILMN_2225548 | ZNF521 | -1.43 | ILMN_1256012 | Zfp521 | -1.68 | -1.56 |
| ILMN_1700432 | ITPKB | -1.16 | ILMN_2438338 | Itpkb | -1.86 | -1.51 |
| ILMN_1735353 | IRX1 | -1.86 | ILMN_1218204 | Irx1 | -1.14 | -1.50 |
| ILMN_1669323 | BACE2 | -1.21 | ILMN_2720083 | Bace2 | -1.77 | -1.49 |
| ILMN_1697268 | EMILIN2 | -1.24 | ILMN_2772281 | Emilin2 | -1.72 | -1.48 |
| ILMN_1778991 | NFIB | -2.28 | ILMN_2726585 | Nfib | -0.67 | -1.47 |
| ILMN_1752510 | FAM13A | -1.98 | ILMN_1224427 | Fam13a | -0.94 | -1.46 |
| ILMN_1810093 | TSPAN2 | -0.68 | ILMN_1234698 | Tspan2 | -2.23 | -1.46 |
| ILMN_1720048 | CCL2 | -0.86 | ILMN_1245710 | Ccl2 | -1.96 | -1.41 |
| ILMN_1794038 | FAM49A | -0.73 | ILMN_2983525 | Fam49a | -1.99 | -1.36 |
| ILMN_1724480 | AXIN2 | -1.56 | ILMN_2896314 | Axin2 | -1.15 | -1.35 |
| ILMN_1695945 | MEIS2 | -1.50 | ILMN_2850391 | Meis2 | -1.19 | -1.34 |
| ILMN_1699489 | TUBB6 | -1.57 | ILMN_2718217 | Tubb6 | -1.06 | -1.31 |
| ILMN_2406304 | PDZRN3 | -1.59 | ILMN_3156010 | Pdzrn3 | -0.97 | -1.28 |
| ILMN_1683415 | CAMK2D | -1.70 | ILMN_1216561 | Camk2d | -0.84 | -1.27 |
| ILMN_1749868 | FAM171A1 | -1.55 | ILMN_3158937 | Fam171a1 | -0.97 | -1.26 |
| ILMN_2083469 | IRS2 | -1.09 | ILMN_3144164 | Irs2 | -1.43 | -1.26 |
| ILMN_2106902 | FOXN3 | -1.38 | ILMN_2844097 | Foxn3 | -1.11 | -1.25 |
| ILMN_2096784 | TFAP2C | -1.11 | ILMN_2783225 | Tcfap2c | -1.36 | -1.24 |
| ILMN_2222065 | FZD1 | -1.18 | ILMN_2683698 | Fzd1 | -1.30 | -1.24 |
| ILMN_1668996 | C1QBP | -0.72 | ILMN_1225373 | C1qbp | -1.64 | -1.18 |
| ILMN_1732555 | B4GALT6 | -0.99 | ILMN_1250019 | B4galt6 | -1.17 | -1.08 |
| ILMN_2375002 | MAP4K4 | -1.31 | ILMN_1233116 | Map4k4 | -0.68 | -1.00 |
| ILMN_1738816 | FOXO1 | -1.08 | ILMN_2656498 | Foxo1 | -0.90 | -0.99 |
| ILMN_2117171 | LMO4 | -1.01 | ILMN_2642403 | Lmo4 | -0.92 | -0.97 |
| ILMN_1798602 | PCF11 | -0.94 | ILMN_3160194 | Pcf11 | -0.98 | -0.96 |
| ILMN_2229839 | RAP2B | -1.06 | ILMN_1254179 | Rap2b | -0.83 | -0.95 |
| ILMN_1665319 | NRTN | -0.74 | ILMN_2655498 | Nrtn | -0.99 | -0.86 |

The conserved genes between mouse and human were selected by using the nested F multiple testing adjustments with FDR<0.1 and at least 1.5 fold change. Mouse signature genes for a subset were first selected, then multiple testing adjustments were performed for the human data of these subsets of the ortholog genes. The mouse signature genes that were also significantly differentially expressed in human were defined as the conserved genes. The conserved genes represent those consistently up or down in one subpopulation across the two species.
